# AMF communities associated to *Vitis vinifera* in an Italian vineyard subjected to integrated pest management at two different phenological stages

**DOI:** 10.1038/s41598-020-66067-w

**Published:** 2020-06-08

**Authors:** N. Massa, E. Bona, G. Novello, V. Todeschini, L. Boatti, F. Mignone, E. Gamalero, G. Lingua, G. Berta, P. Cesaro

**Affiliations:** 1 Università del Piemonte Orientale, Dipartimento di Scienze e Innovazione Tecnologica, Viale T. Michel 11, Alessandria, 15121 Italy; 2 Università del Piemonte Orientale, Dipartimento di Scienze e Innovazione Tecnologica, Piazza San Eusebio 5, 13100 Vercelli, Italy; 3 SmartSeq s.r.l., spin-off of the Università del Piemonte Orientale, Viale T. Michel 11, Alessandria, 15121 Italy

**Keywords:** Arbuscular mycorrhiza, Biodiversity

## Abstract

*Vitis vinifera* L. is an economically important crop that can be influenced by soil microorganisms, including arbuscular mycorrhizal fungi (AMF), that establish symbiotic associations with its roots. AMF have beneficial effects on grapevine performance improving water use efficiency and replant success. Most grapevine varieties are susceptible to various diseases, and integrated pest management (IPM) is one of the emerging approaches to perform pest control. In the present study, we examined the AMF communities present in the soil associated to the roots of *V. vinifera* cv. Pinot Noir (comparing them to those present in a soil not affected by grapevine roots), in a vineyard subjected to IPM at two different phenological stages, using 454 Roche sequencing technology. We proposed a new approach to analyze sequencing data. Most of the taxa were included in the family Glomeraceae. In particular, *Glomus* sp. *Rhizophagus* sp. and *Septoglomus viscosum* were present. The family Archeosporaceae was represented only by the genus *Archeospora* sp. Different AMF communities were found in the two soils and the importance of the phenological stage in regulating AMF biodiversity was assessed.

## Introduction

Grapevine is an important perennial crop cultivated worldwide and consumed as fresh or dried fruit or processed to produce wine, grape juice and vinegars, as well as marmalades, jellies, butter and jam. Its production in Italy achieved 8.6 millions of tons and represents 11% of the world production^1^. The moderate consumption of wine^2,3^ and the inclusion of grapes and derivate products in the diet^4^ may result in beneficial effects for human health because it can decrease risk factors associated with cardiovascular and neurodegenerative diseases, cancer and age-related cognitive decline^5^.

Grapevine is highly responsive to local conditions and to agronomic practices, leading to specific characteristics of the different kind of wines^6^. This feature corresponds to the official definition of “terroir” given by the International Organization of Vine and Wine (OIV) as: “*a concept which refers to an area in which collective knowledge of the interactions between the identifiable physical and biological environment and applied vitivinicultural practices develops, providing distinctive characteristics for the products originating from this area. “Terroir” includes specific soil, topography, climate, landscape characteristics and biodiversity features*” (Resolution OIV/Viti 333/2010). Even if climate and soil characteristics associated to the vine “terroir” have been largely studied, interest for the “microbial terroir” of vineyard^7^ has been, so far, less investigated and understood^6^.

Mutualistic plant-microbe interactions offer a novel approach to enhance agricultural productivity also reducing environmental costs, that are mainly due to the massive use of chemical fertilizers. In this context, arbuscular mycorrhizal fungi (AMF) are an important group of soil microorganisms, because they provide an increased interface between roots and soil, so improving the plant nutritional state, especially the phosphatic one^8^. At the same time, AMF provide further advantages for the plants, allowing them to better tolerate biotic and abiotic stresses^8–11^ and ameliorating fruit yield and quality^12–17^. In this respect, the reduction of the biodiversity of the AMF community can negatively impact on plant functionality^8^. Recent studies show that key ecosystem processes are affected by a loss in soil biodiversity^18^ and that land usage has a great impact on it^19^ and, therefore, on the ecosystem services provided by the soil microbiota^20^. The composition of AMF communities could also vary with the plant phenological stage, especially during flowering and ripening, as these phases are accompanied by changes in root-exudate composition^21^.

Previous reports suggest a host preference among *Vitis* and AMF^22^. It is also known that grapevines are dependent on AMF for growth and development^23,24^. In addition, native AMF in a given zone are often reported to be more effective than the non-native ones^25^; hence, the importance of better knowing the structure of native AMF communities, also for possible applications for sustainable agricultural ecosystems^26^, has grown over time. Few works are available in the literature about the study of AMF biodiversity in orchard tree plants^27,28^ or in vineyards, using a molecular approach, considering both AMF present in the soil and those associated to vine roots^22,26,29–32^.

Moreover, another important factor that can influence the composition of soil microbial community is the type of vineyard management (e.g. conventional, biological and/or integrated), as the biocide application can negatively affect soil microorganisms, including AMF^33,34^. In particular, the integrated pest management (IPM) approach contemplates the use of selective and less dangerous pesticides, distributed in lower amount and with a lower frequency in respect to a conventional plan^35^.

**Figure 1:**
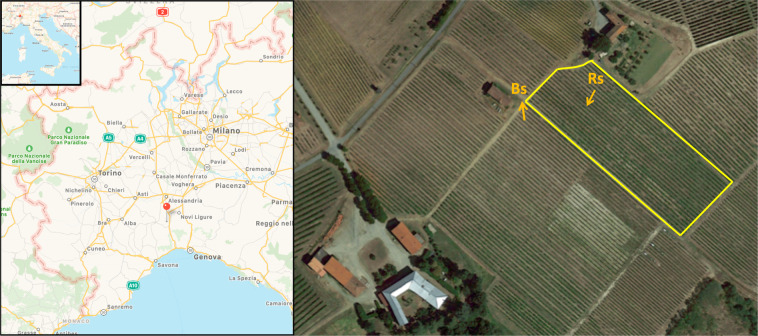
Vineyard view and sampling map. The vineyard is located in “Tenuta Cannona” at the Agrion “Fondazione per la ricerca, l’innovazione e lo sviluppo tecnologico dell’agricoltura piemontese”. GIS map of the two sampling sites: one in an area without grapevines, just ouside the borders of the vineyard (Bulk soil or Bs) and one inside the vineyard (Root associated soil or Rs). The image was produced by the authors using QGIS v. 2.10 Pisa (QGIS Development Team, 2015. Quantum GIS Geographic Information System. Open Source Geospatial Foundation Project. http://qgis.osgeo.org).

To our knowledge, no information exists about AMF biodiversity in IPM vineyards. So, this work has a dual purpose: the first one is to characterize the AMF community present in the soil associated to the roots of *V. vinifera* cv. Pinot Noir, in a vineyard, located in Piedmont (Italy) (Fig. 1) and subjected to IPM, at two different phenological stages of the plant (flowering and fruit development); the second one is to propose a new approach to study the AMF communities, particularly to decipher the identity of the “*de novo* AMF taxa”.

## Results

### Assessment of root colonization

Grapevine roots grown in Rs were colonized by AMF in both samplings, but no significant differences between the two samplings were detected. In particular, the frequencies of colonization were 97.4% ± 1.6 at the first and 99.7% ± 0.3 at the second sampling. The degrees of mycorrhizal colonization were 42.4% ± 6.4 and 37.2% ± 6.9 and the arbuscule abundances were 23.7% ± 5.6 and 20.4% ± 4.5, in the first and the second sampling, respectively.

### Taxa abundance and analysis of biodiversity

The real number of sequences obtained from each replicate for Bs and Rs, is reported in Table 1. In general, the average number of sequences obtained was about 9,000. The number of sequences was then normalized in order to compare the different samples.

**Table 1:** Number of sequences obtained from the different replicates of Bulk soil (Bs) and soil associated with the roots of *V. vinifera* cv. Pinot Noir (Rs) at the two sampling times (1 S = first sampling in May; 2 S = second sampling in July).

Soil sample	Replicate	number of sequences		Avarage number of sequences
		1S	2S	
Bs	1	3,773	9,176	9,094
	2	10,881	5,222	
	3	10,592	3,955	
	4	10,357	12,239	
	5	9,869	6,664	
Rs	1	7,916	8,701	8,784
	2	6,491	10,988	
	3	10,099	8,670	
	4	10,404	10,322	
	5	9,011	11,099	

**Figure 2:**
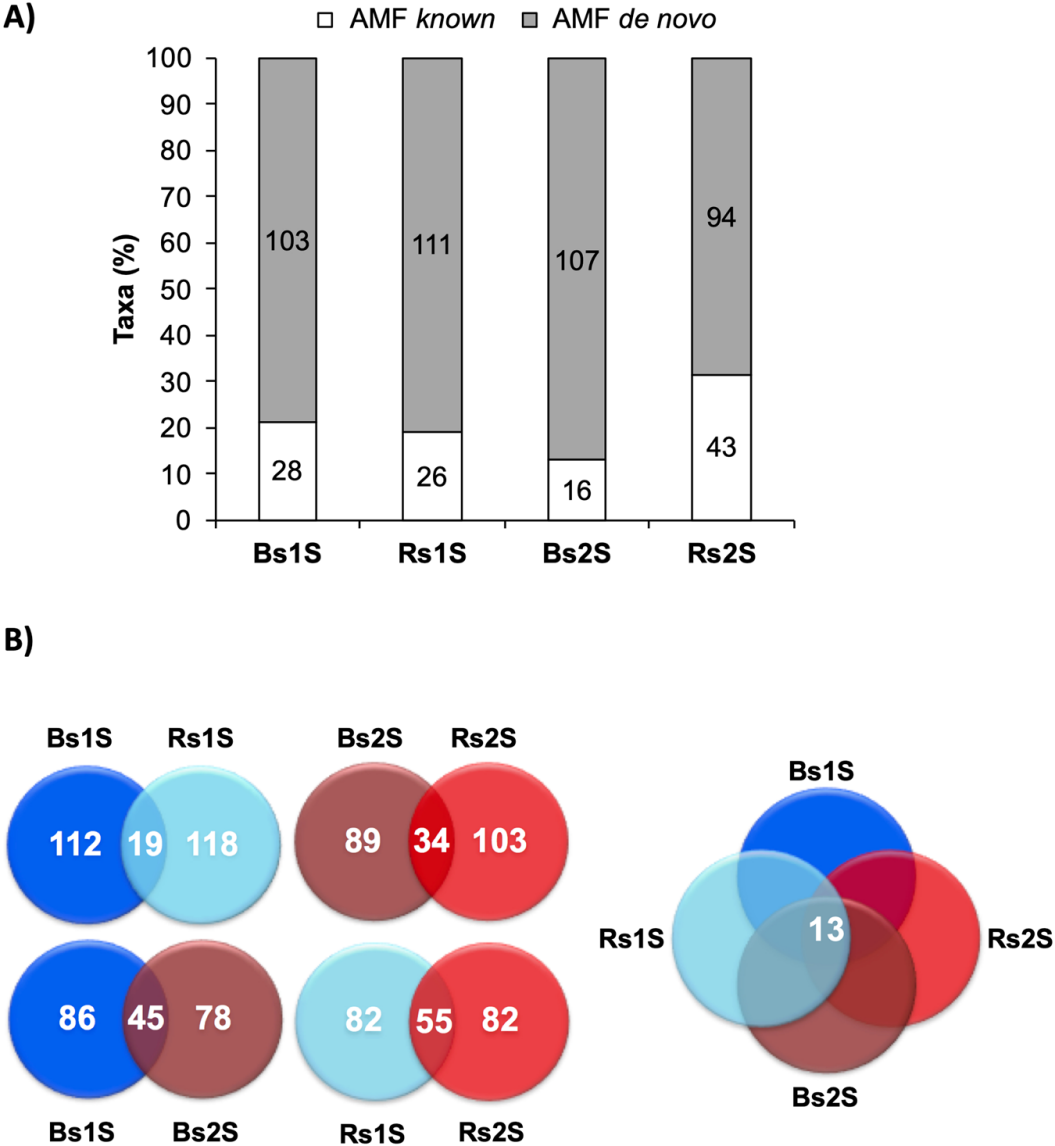
**A) Taxa richness**. Taxa obtained from Bulk soil (Bs) and the soil associated with the roots of *V. vinifera* cv. Pinot Noir (Rs) during the two sampling times (1 S = flowering and 2 S = fruit development). Bars represent the percentage, while labels inside the bars indicate the real number of taxa of “AMF known” (white) and “AMF *de novo*” (grey). **B) Venn diagrams**. Venn diagrams representing the number of taxa, that were exclusive or common to: a) Bulk soil (Bs) and the soil associated with the roots of *V. vinifera* cv. Pinot Noir (Rs) in the first (1 S) or in the second (2 S) sampling time (upper part on the left of the figure); b) the first (1 S) and the second (2 S) sampling times in Bs or in Rs (lower part on the left of the figure); c) the four soil samples (Bs1S, Rs1S, Bs2S, Rs2S - on the right of the figure). The Venn diagrams were drawn using the freely available Venny version 2.1 software (http://bioinfogp.cnb.csic.es/tools/venny).

The rarefaction curves (Fig. S1), reaching a plateau, show that the number of obtained sequences was sufficient to deeply describe these soil samples. A total of 528 taxa (359 univocal), were obtained from the two soils at the two sampling times, including 113 “AMF known” and 415 “*de novo*” ones (Tables S1 and S2). In Fig. 2A the distribution of taxa obtained for each sample is represented. In the case of Bs, 131 and 123 taxa were obtained in the first and the second sampling time, respectively. In particular, a decreased number of ″AMF known″ taxa (from 28 to 16) occurred in Bs2S sample if compared to Bs1S (factor time), while the number of ″AMF *de novo*″ taxa was similar (103 and 107, respectively). The total number of sequences in Rs samples was the same in both samplings (137) despite an increase of ″AMF known″ taxa (from 26 to 43) in Rs2S if compared to Rs1S (factor time).

Bs1S and Rs1S or Bs2S and Rs2S (factor space) shared 19 and 34 taxa, respectively. Bs contained the same 45 taxa in both sampling times, while, the number of taxa common in the two samplings was 55 in Rs. Finally, thirteen taxa were common to all samples (Fig. 2B).

**Figure 3:**
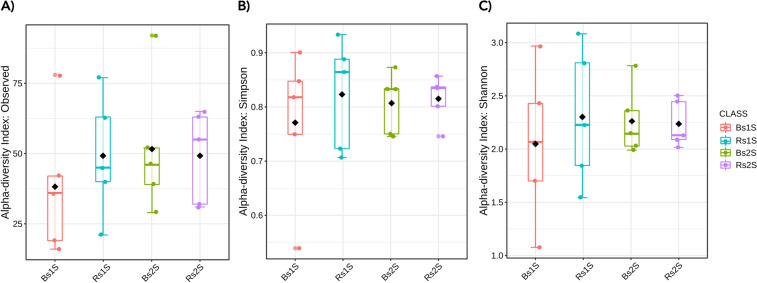
Alpha diversity indices. **A) Number of observed AMF species** (p-value 0.7073), **B) Simpson index** (p-value 0.94376) and **C) Shannon’s Index** (p-value 0.92697) of biodiversity detected in Bulk soil (Bs) and in soil associated with the roots of *V. vinifera* cv. Pinot Noir (Rs) at the two sampling times (1 S = flowering and 2 S = fruit development). Alpha diversity analysis was performed using the phyloseq package of MicrobiomeAnalyst, a free available on-line software (https://www.microbiomeanalyst.ca).

The calculation of observed species, Simpson and Shannon indices was performed to compare AMF diversity (Fig. 3). For all indices, in both sampling times, the median values were not statistically significant different in the two type of soil.

**Figure 4:**
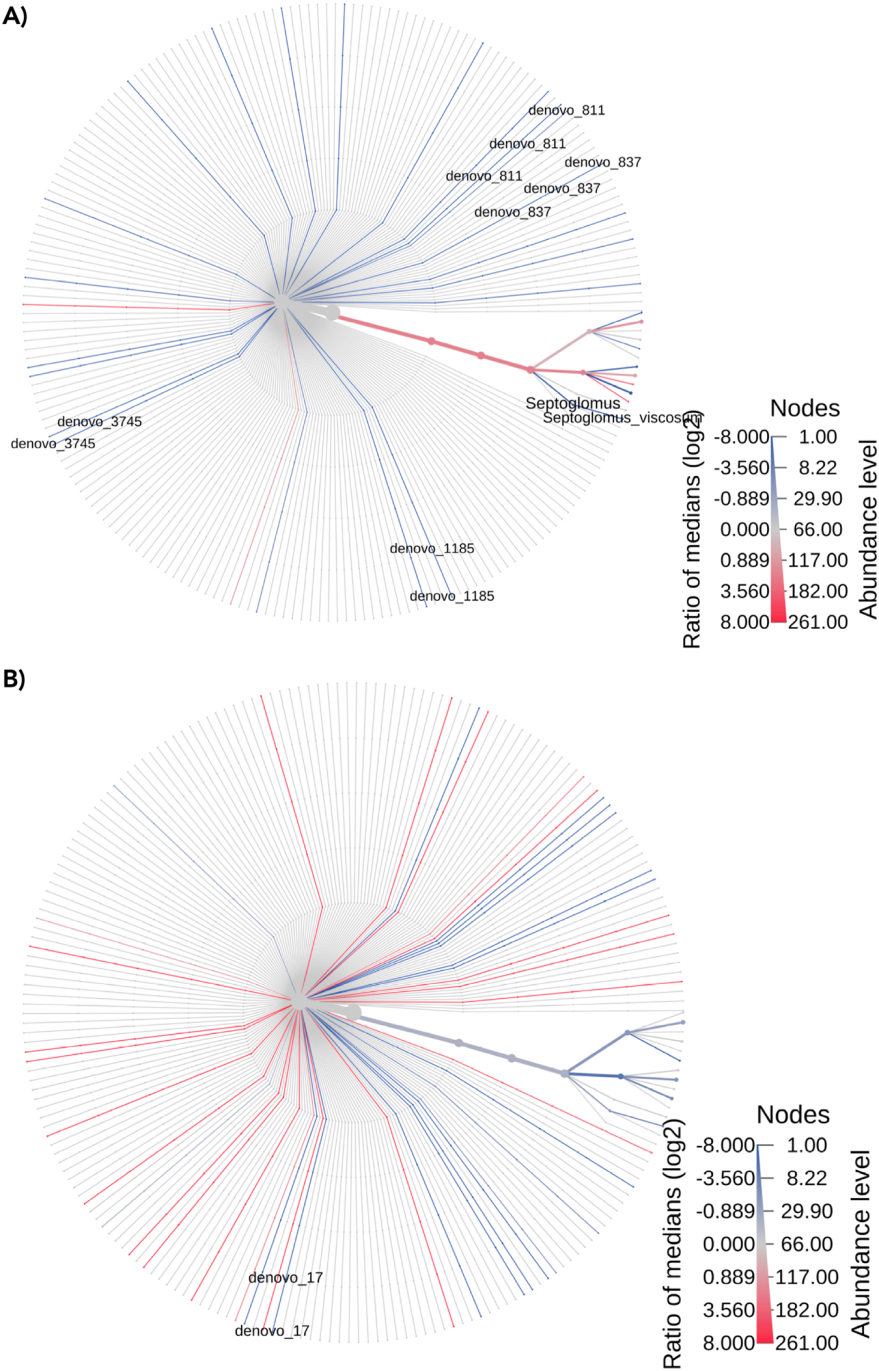
Heat tree of factor Time. Heat Trees report the effect of the sampling time on hierarchical structure of taxonomic classifications (median abundance, non-parameter Wilcoxon Rank Sum test). (**A**) Bs1 vs Bs2; (**B**) Rs1 vs Rs2. Heat tree analysis was performed using R metacoder package of MicrobiomeAnalyst, a free available on-line software (https://www.microbiomeanalyst.ca).

**Figure 5:**
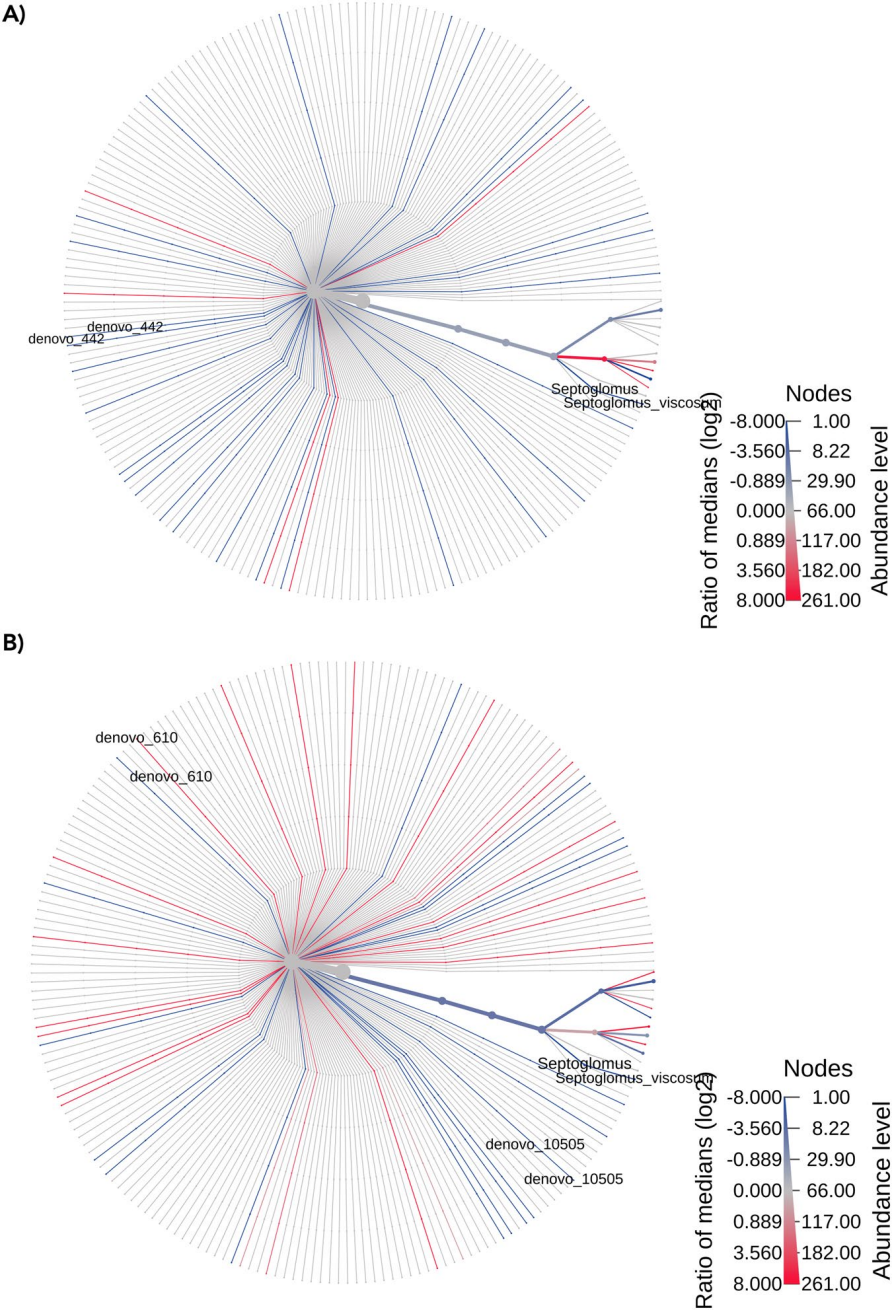
Heat tree of factor Space. Heat Trees report the effect of the presence of *V. vinifera* roots on hierarchical structure of taxonomic classifications (median abundance, non-parameter Wilcoxon Rank Sum test). **A)** Bs1 vs Rs1; **B)** Bs2 vs Rs2. Heat tree analysis was performed using R metacoder package of MicrobiomeAnalyst, a free available on-line software (https://www.microbiomeanalyst.ca).

The time and space effect on the AMF community are represented in the heat trees (Figs. 4 and 5). In particular, Fig. 4A (time effect in Bulk soil) displays the increase (blue lines) of *Septoglomus viscosum*, of *de novo_*811, *de novo_*837, *de novo_*1185 and *de novo_*3745 taxa in Bs2S in respect to Bs1S. Figure 4B (time effect in the soil associated to *V. vinifera* roots) shows the increase of the AMF community in the Rs2S samples in respect to Rs1S, particularly of the *de novo*_17 taxa. Heat trees presented in Fig. 5A (space effect) displays the increase of *Septoglomus viscosum* and of *de novo_*442 taxa in Rs1S in respect to Bs1S, while Fig. 5B displays the increase of *Septoglomus viscosum* and of *de novo_*10505 taxa and the decrease of *de novo_*610 in Rs2S in respect to Bs2S. The table reporting the statistical details of these results are showed in Table S3.

**Figure 6:**
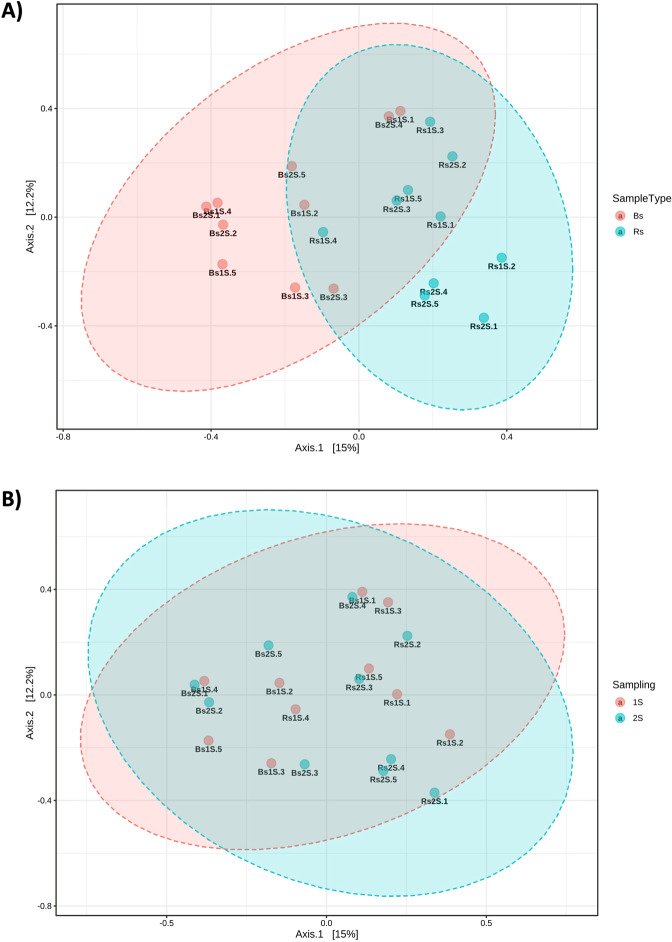
Principal Coordinates Analysis (PCoA). Comparison by PCoA of the ecological distance (with the Bray–Curtis distance based method) of the different compartments. **(A)** Soil type effect (Bs = Bulk soil, Rs = soil associated with the roots of *V. vinifera* cv. Pinot Noir), PERMANOVA F-value: 2.3313; R-squared: 0.11467; p-value <0.001. **(B)** sampling time effect (1 S = flowering and 2 S = fruit development), PERMANOVA F-value: 0.75825; R-squared: 0.040422; p-value <0.845. Beta diversity analysis was performed using the phyloseq package of MicrobiomeAnalyst, a free available on-line software (https://www.microbiomeanalyst.ca).

Data analyzed by PCoA underlined a different structure between the two soils (space effect) (PERMANOVA p-value <0.001); Bs and Rs samples were clearly separated along the x-axis (Fig. 6A). PCoA results also revealed that time (Fig. 6B) is not a statistical significant parameter determining the difference between the AMF community analyzed.

**Figure 7:**
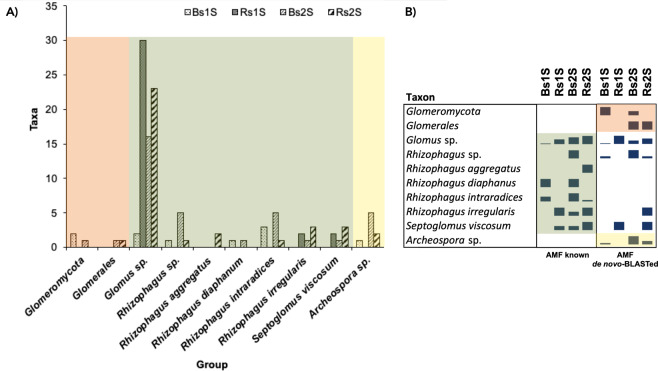
Taxa classification. (**A**) Number of sequences belonging to the different taxa - obtained grouping ″AMF *de novo*-BLASTed″ taxa with the ″AMF known″ ones, on the basis of the group to which they belonged - in the different compartments (Bs = Bulk soil, Rs = soil associated with the roots of *V. vinifera* cv. Pinot Noir) at the two sampling times (1 S = flowering and 2 S = fruit development). (**B**) Relative abundance of taxa, obtained considering separately “AMF known” and “AMF *de novo*-BLASTed”, belonging to each group, in Bulk soil (Bs) and in the soil associated with the roots of *V. vinifera* cv. Pinot Noir (Rs) at the two sampling times (1 S = flowering and 2 S = fruit development).

To give a name to the ″AMF *de novo*″ taxa, they were BLASTed against NCBI database (Table S2). Then, these ″*de novo*-BLASTed″ taxa were grouped with the ″AMF known″ ones, according to the belonging taxon. Two taxa corresponded to higher AMF classification levels (subphylum and order; Fig. 7A - orange area on the left). Many taxa were included in the family Glomeraceae (Fig. 7A - central green area); *Glomus* sp. was the most abundant taxon (30 taxa in Rs1S). All the other remaining taxa of the family Glomeraceae belonged to the genus *Rhizophagus*, with the exception of *Septoglomus viscosum*. All the taxa belonging to the family Archeosporaceae were included in only one genus (*Archeospora* sp.; Fig. 7A - yellow area on the right).

Most of the taxa were detected in both soils, with the exception of unknown Glomeromycota and *Rhizophagus diaphanus* that were present only in Bs in the two sampling times.  Moreover, *Rhizophagus aggregatus* was found only in the Rs in the second sampling time (Fig. 7A).

However, considering the “AMF *de novo*” taxon identification by NCBI, the obtained results did not give new information about the occurrence of taxa, if compared to the knowledge acquired with “AMF known” taxa, with the exception of the genus *Archeospora* (Fig. 7B). The “AMF known” taxa belonged only to the family Glomeraceae (Fig. 7B). All the identified “AMF *de novo*” taxa corresponded to uncultured AMF, except five: one *de novo Glomeromycota* sp., two *de novo Glomus* sp., one *de novo Rizophagus irregularis* and one *de novo Septoglomus viscosum* (Table S2).

**Figure 8:**
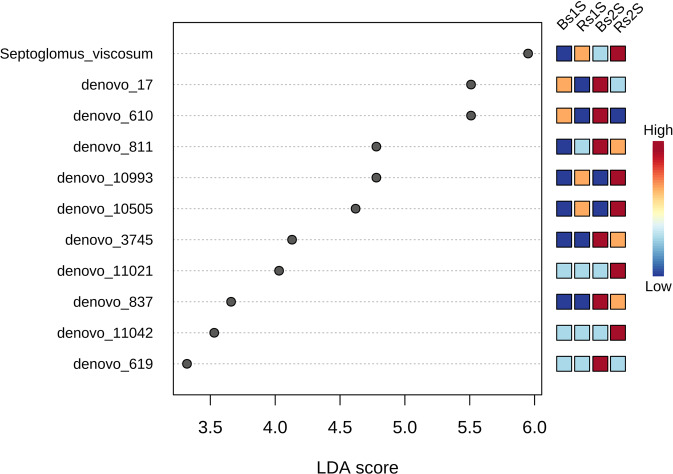
Linear discriminant analysis Effect Size (LEfSe). LEfSe results using non-parametric factorial Kruskal-Wallis (KW) sum-rank test. Adjusted p-value cutoff = 0.05 and LDA score = 1.0; *de novo_*17 = Uncultured *Archeospora* (LDA score 5.51); *de novo_*610 = Uncultured *Rhizophagus* (LDA score 5.51); *de novo_*811 = Uncultured *Rhizophagus* (LDA score 4.78); *de novo_*10993 = Uncultured *Glomus* (LDA score 4.78); *de novo_*10505 = *Septoglomus viscosum* (LDA score 4.62); *de novo_*3745 = Uncultured *Archeospora* (LDA score 4.13); *de novo_*11021 = Uncultured *Glomus* (LDA score 4.03); *de novo_*837 = Uncultured *Glomus* (LDA score 3.66); *de novo_*11042 = Uncultured *Glomus* (LDA score 3.53), *de novo_*619 = Uncultured *Glomus* (LDA score 3.32). LEfSe analysis was performed with MicrobiomeAnalyst, a free available on-line software (https://www.microbiomeanalyst.ca).

The Linear discriminant analysis Effect Size (LEfSe) results, presented in Fig. 8 and Table S4, showed the 11 taxa that better explained the differences in the analyzed AMF community. In particular, *Septoglomus viscosum* showed a LDA score of 5.95 (p-value 0.0037752), the highest values in Rs2S followed by Rs1S. Other important taxa present in the soil associated to *Vitis vinifera* roots (Rs) were *de novo_*811 that was Uncultured *Rhizophagus* (LDA score 4.78), *de novo_*10993 that was Uncultured *Glomus* (LDA score 4.78), *de novo_*10505 that was *Septoglomus viscosum* (LDA score 4.62), *de novo_*3745 that was Uncultured *Archeospora* (LDA score 4.13), *de novo_*11021 that was Uncultured *Glomus* (LDA score 4.03), *de novo_*837 that was Uncultured *Glomus* (LDA score 3.66), *de novo_*11042 that was Uncultured *Glomus* (LDA score 3.53). On the contrary, the three *de novo* taxa, *de novo_*17 that was Uncultured *Archeospora* (LDA score 5.51) *de novo_*610 that was Uncultured *Rhizophagus* (LDA score 5.51) *de novo_*619 that was Uncultured *Glomus* (LDA score 3.32) mostly explained the differences in Bs.

## Discussion

The AMF symbiosis is probably the most widespread beneficial interaction between plants and microorganisms; it has been shown that AMF are able to colonize grapevine roots^6^. Our interest in characterizing the AMF community associated to grapevines is justified by both economic and historical reasons. In fact, Piedmont is the second Italian region for areas dedicated to vineyards and wine production^36^ and, since 2014, the hills of the Piedmont area covering the Langhe, Roero and Monferrato have been included on the UNESCO World Heritage list (http://whc.unesco.org/en/list/1390). So, the study of the structure of AMF communities has become crucial to characterize the “terroir” of a variety largely cultivated in Piedmont such as Pinot Noir.

This work reached the two goals: 1) to characterize the biodiversity of AMF in two types of soil (Rs and Bs, that were influenced or not by the presence of grapevine roots, respectively) and at two different stages of plant growth (flowering and fruit development) in an IPM vineyard and 2) to propose a new approach to decipher the identity of the “*de novo* AMF taxa”.

In order to investigate whether the AMF present in the Rs were able to colonize *V. vinifera* roots, the mycorrhization degree were assessed and the plants resulted to be colonized by AMF, with values similar to those previously reported in the literature^24,26^, without significant differences between the two considered plant phenological stages. In fact, Schreiner^24^ reported that arbuscular colonization of *V. vinifera* cv. Pinot Noir roots increased prior to bud break in the spring, reached a high level (50–60% root length) in early summer, and remained high until after the time of leaf senescence in late fall.

Regarding pyrosequencing analysis, the resulting fragments were 700 bp long, having a larger size if compared to those obtained in other published works^22,31^. Following the criteria suggested by Lindahl and colleagues^37^ and Hart and coworkers^38^ we applied coverage ≥ 80% and similarity of sequences ≥ 97% in order to identify taxa at the species level. We found a total of 528 taxa: this result is consistent to what was observed by Holland at al.^39^ that obtained 816 taxa studying the AMF biodiversity in a Canadian area using the SSU rDNA marker. On the basis of the statistical analysis, we can assert that: 1) the AMF biodiversity was not different both in space and time; 2) the most number of observed taxa belonged to the family Glomeraceae (*Septoglomus viscosum*) in both soils and at the two phenological stages (sampling times), even if they were represented by different taxa.

The first point indicates that, at flowering time, the number of species of AMF in this vineyard was higher in the soil associated to the roots than in Bulk soil, while no differences in number were observed at fruit development stage. This last observation could be linked with the presence of herbaceous plants in Bs that, with their root exudates, can induce an increase of the number of AMF species^22^. However, the involved taxa were different between the two soils: for example, in the second sampling, only 34 taxa were common to both soils, while 89 were exclusive of Bs2S and 103 of Rs2S. Moreover, only 13 taxa were shared by the two soils at the two phenological stages.

It is noteworthy that Bs and Rs are characterized by AMF communities with different structure (also confirmed by PCoA analysis), although they have similar biodiversity indices: the Simpson index - which is based on the probability to assign two independent individuals, taken randomly from the community, to the same species – and the Shannon index - an entropy measurement that increases according to the number of species in the sample – the second index is particularly sensitive to the number of rare species in a community^40^, that in our work were represented by the taxa with a low number of sequences. Our results are in agreement with those obtained by Schreiner and Mihara^30^ in a similar study, that analyzed the seasonal effect in vineyards of different ages and where no time effect was observed; however, the second sampling time was different, because they considered the onset of ripening in the middle of September, instead of fruiting time (July) as in our work. However, it is reported in the literature that seasonality has an effect on the biodiversity of AMF associated with crops other than grapevines^41–43^. Finally, the comparison of phylogenetic distance obtained by PCoA underlined that Bs differed from Rs, confirming what previously presented.

Despite the primers used in this study were specific for AMF, and so they were designed to target also representatives of Claroideoglomeraceae, Acaulosporaceae, Gigasporaceae and Diversisporaceae, no fungal sequences belonging to these families were detected. In line with other studies that describe AMF biodiversity in vineyard soils and on grapevine roots, many fungal taxa corresponded to Glomeraceae, which is known to be the most abundant taxon in agricultural lands^44,45^, and also in vineyards^6,22,31,32^. Fungi belonging to this family have a high growth rate and a rapid recovery of hyphal network following disturbance due to the agricultural practices^6,45^. *Glomus* sp. was the most abundant taxon, all the other remaining taxa of the family Glomeraceae belonged to the genus *Rhizophagus*, with the exception of *Septoglomus viscosum*.

In agreement with Schreiner and Mihara^30^ and to Balestrini *et al*.^32^ we also found taxa belonging to the family Archeosporaceae that in this work were represented only by one genus: *Archeospora* sp. The vineyard analyzed in this work was planted 25 years ago and, in time, a selection of the AMF might have been induced by the grapevines, as proposed also by Schreiner and Mihara^30^, that reported a lower AMF biodiversity in older grapevines. This selection could be influenced also by the soil characteristics: temperature, pH, moisture content, nutrient status and in addition the host identity and diversity are known to affect AMF biodiversity^6,32^. The influence of the rootstock (in this case SO4) is another important factor that must be taken into account in the interpretation of the obtained data. Schreiner^46^, monitoring grapevine root colonization for three years, showed that little differences in the ability to form mycorrhizas among rootstocks exist. So, data obtained in this work could be generalizable to vineyards where this rootstock is present and where there are comparable situations of soil features and climate.

It is noteworthy that the AMF populations (with Glomeraceae and Archeosporaceae as prevalent taxa) found in this work, performed in an IPM vineyard, were similar to those obtained in other vineyards managed by different practices^30,32,39^. IPM management contributes to maintain biodiversity within the vineyard, as it limits the pesticide application and it employs plant cover to improve the biodiversity of soil microorganisms^28^. In fact, AMF diversity or community composition can be affected by a number of uncontrolled variables, such as soil tillage, cover crops, manure, quality and quantity of herbicides, pesticides and fertilizers^28^.

Regarding the methodological approach used in this work to study the AMF communities, we propose a new method that does not generate OTUs in the first analysis phase. We adopt the alignment of each single sequence against the reference database, in order to remove the 3% error deriving from the clusterization in groups for OTU definition. In fact, we did not chose a sequence representative of a group of multiple sequences (OTU), but, in our method, the group corresponds to a single sequence. This approach is useful to discriminate those taxa that are very similar to each other, especially in the case of fungi, which are multinucleated organisms.

Concluding, our data showed that different AMF communities were associated with the two soils (Bs and Rs) confirming the importance of the presence of host plant in regulating AMF community structure.

Moreover, we would underline that pyrosequencing is a powerful technology that provides a large number of sequences, useful to describe the biodiversity of an ecosystem. However, the specific features of the biological system under analysis should not be disregarded. In fact, when studying the biodiversity of organisms, such as AMF, represented by a low number of species, greatly increasing the depth of the analysis (high number of sequences) does not provide an added value to the real biological data.

## Material and Methods

### Soil Sampling

The experimental vineyard is located in the hills of Piedmont close to Carpeneto (AL, Italy - altitude: 286 m a.s.l., latitude: 44.683706°N and longitude: 8.6258889°E). This vineyard was managed according to IPM. In Europe, IPM is not yet regulated; however, its general principles are listed in the Annex III of Directive 2009/128/EC.

Two soils, one not affected by grapevine root presence and referred here as Bulk soil (Bs), and one associated with the roots of *V. vinifera* cv. Pinot Noir grafted onto SO4 rootstock (Rs), were analys ed. Soil samplings were performed in May (Bs1S and Rs1S) and July (Bs2S and Rs2S) 2014, corresponding to flowering and fruit development, respectively (Fig. 1). Bs was collected in a zone close, but external to the vineyard, without grapevines and partially covered with grasses. Grapevine roots entrapped in the soil cores collected close to the stem were considered for the sampling of the soil associated to the roots and the soil adhering to these roots was removed (Rs). Five samples for each soil (Bs or Rs) and for each time (1S or 2S), were collected at a depth of 30 cm, corresponding to the topsoil, after removing the surface layer (3–5 cm). For each sample, three soil cores were taken. As recommended by the Italian law (GU 179/2002) for soil characterization analysis, the three soil cores taken in the proximity of each plant were pooled and mixed to prepare one sample. Soil samples were stored at −20 °C for 1 week until DNA extraction.

The two soils were clay loam, with a neutral pH. Rs presented higher values of organic matter, N, C/N ratio, P_2_O_5_ if compared to Bs^47^. Temperature, humidity and rainfall trends are reported in Fig. S2.

As fully described in Novello *et al*.^35^, grapevines were chemically weeded and treated against *Peronospora* spp., *Oidium* spp. and *Botrytis cinerea*; moreover, two insecticide treatments were applied, following IPM guidelines. The phytosanitary treatments were carried out in the same way on both soils: even though there were no plants, the treatment was performed also in Bs in order to cover the whole area.

### Assessment of root colonization

Mycorrhizal frequency, the level of mycorrhizal colonization and the content of arbuscules were evaluated microscopically according to the method of Trouvelot *et al*.^48^. Briefly, 30 randomly chosen 1 cm long pieces of grapevine roots were cut, cleared for 90 min at 80 °C in 10% KOH, stained with 1% methyl blue in lactic acid, and mounted onto slides. Results were statistically analyzed by ANOVA followed by Fisher’s test, with significance cut-off at 0.05.

### DNA extraction and amplification

DNA was extracted from five samples both of Bulk soil (Bs) and of the soil associated with the roots of *V. vinifera* (Rs), collected during flowering (1 S) or fruiting (2 S), using the Power Soil R DNA Isolation Kit (MO BIO Laboratories, Inc., Carlsbad, CA, United States) following the manufacturer’s instructions. Then, DNA was employed as template for an hemi-nested PCR using LR1 and FLR2^44^ primers for the first amplification and LR1 and FLR4^44^ primers tagged with Multiplex Identifier sequences for 454 Pyrosequencing (Roche Diagnostics S.p.A.) for the second one. In particular, the FLR2 primer used in the first reaction is specific for fungi, while the FLR4 primer used in the second reaction is specific for Glomeromycota^49^. The reactions were performed in a Techne thermocycler (TC512, Bibby Scientific, Riozzo di Cerro al Lambro, Italy) at the following conditions: initial denaturation 94 °C for 5 min; then 94 °C for 1 min, 60 °C for 1 min, and 72 °C for 2 min or 5 min (for the first and the second amplification, respectively) for 30 cycles; finally, an elongation step at 72 °C for 5 min. Each reaction mixture (20 μl) contained five microliters of genomic DNA diluted at different levels (1:10, 1:50, 1:100 - for the first amplification) or the first PCR products diluted 1:100 and 1:500 (for the second amplification), dNTPs 500 μM, MgCl_2_ 1.5 mM, 2 μl PCR buffer 10×, 500 nM of both forward and reverse primers and 0.4 U of Taq DNA Polymerase (Thermofisher).

The products of the second PCR (size 700-bp) were used for pyrosequencing using 454 technology. DNA-carrying beads were loaded on a PicoTiter (Roche Diagnostics S.p.A.) plate and surrounded by enzyme beads (sulfurylase luciferase). The light signals were represented in flow grams and analyzed; a nucleotide sequence was determined for each read with the GS Amplicon Variant Analyzer software.

### Bioinformatic analysis

Data were analyzed using a custom bioinformatic pipeline. Raw sequence reads were demultiplexed to obtain a single file for each sample (consisting of 5 biological samples, 2 plant phenological stages and 2 soils - Bs and Rs). During this process, reads that met the following criteria were discarded: (1) read length <than 200 nucleotides, (2) average Phred quality score^50^ < than 25, (3) presence of at least one ambiguous base inside the read. Then, an alignment of each sequence was performed against our AMF LSU rDNA database, consisting of 3.803 univocal sequences downloaded from the on-line sources: EBI and SILVA databases, and http://www.amf-phylogeny.com/amphylo_species.html^51^. This database was prepared starting from all the LSU sequences (except those named “uncultured”), then, a selection was made considering only the fragments delimited by LR1 and FLR4 primers. The alignment of each sequence was performed using BLASTN^52^, and, in order to identify the taxa at species level (named “known”), two criteria were applied: coverage ≥ 80% and similarity of sequences ≥ 97% according to Lindahl *et al*.^37^ and Hart *et al*.^38^. Chimeras were also removed with these criteria. All the sequences that did not meet both the above mentioned criteria, were subsequently aligned against themselves: all the sequences with coverage ≥ 80% and similarity of sequences ≥ 97% when compared one to each other, were grouped together and each group was named “*de novo*”.

In order to perform bioinformatic analysis, a sequence database containing the results normalized at 7,000 (an average number deduced comparing the richness of sequences obtained in each sample replica) was produced. The rarefaction curves were plotted with the RAM package of R^53^ (Fig. S1).

### Taxa abundance and biodiversity analysis

Taxa abundance analysis was calculated considering only the taxa present with at least 10 sequences in one replicate in order to ensure that the identification was not due to sequencing errors and the median value of the number of sequences for each taxon was calculated. Then, to give a name to the “*de novo*” taxa, they were BLASTed against NCBI database (Table S2).

To compare the abundance and the degrees of richness and evenness of AMF in Bs and Rs, the Venn diagrams were drawn using the freely available Venny version 2.1 software (http://bioinfogp.cnb.csic.es/tools/venny)^53^.

In order to compare the two analyzed AMF communities, a further analysis by MicrobiomeAnalyst, a free available on-line software (https://www.microbiomeanalyst.ca), was carried out, as reported in Berlanas and collaborators^54^ and also suggested in Sergaki *et al*.^55^. In particular, Alpha diversity analysis was performed using the phyloseq package^56^. The results were plotted across samples and reviewed as box plots for each group or experimental factor. Further, the statistical significance of grouping based on experimental factor is also estimated using either parametric or non- parametric tests.

Heat tree method was used to compare abundance at species taxonomic level for each pair of factors (time and space) in the metadata variable. Heat Tree uses hierarchical structure of taxonomic classifications to quantitatively (median abundance) and statistically (non-parameter Wilcoxon Rank Sum test) depict taxon differences among communities. It generates a differential heat tree to show which taxa are more abundant in each treatment. Heat tree analysis was performed using R metacoder package^57^. The significant cut off used was 0.05.

Beta diversity analysis was performed using the phyloseq package^56^. Principle Coordinate Analysis (PCoA) using Bray-Curtis distance based method was applied and the statistical significance of the clustering pattern in ordination plots were evaluated using Permutational ANOVA (PERMANOVA). Moreover, LEfSe analysis using non-parametric factorial Kruskal-Wallis (KW) sum-rank test was performed. Features were considered to be significant based on their adjusted p-value. Adjusted p-value cutoff = 0.05 and LDA score = 1.0 were applied.

## Supplementary information

Supplementary information.

## Data Availability

The genomic datasets generated and/or analyzed during the current study are available in NCBI using BioProject ID: PRJNA508468 containing the following BioSamples (SAMN10525947, SAMN10525948, SAMN10525949, SAMN10525950). Project Name: AM fungal community associated to *Vitis vinifera* in a Northern Italy vineyard subjected to integrated pest management.

## References

[1] Organisation, I. 2019 Statistical Report on World Vitiviniculture. (2019).

[2] Georgiev V, Ananga A, Tsolova V (2014). Recent advances and uses of grape flavonoids as nutraceuticals. Nutrients.

[3] Artero A, Artero A, Tarín JJ, Cano A (2015). The impact of moderate wine consumption on health. Maturitas.

[4] Vislocky LM, Fernandez ML (2010). Biomedical effects ofgrape products. Nutr. Rev..

[5] Torres, N., Antolín, M. C. & Goicoechea, N. Arbuscular Mycorrhizal Symbiosis as a Promising Resource for Improving Berry Quality in Grapevines Under Changing Environments. *Front. Plant Sci*. **9**, (2018). 10.3389/fpls.2018.00897 PMC6034061 30008729

[6] Trouvelot S (2015). Arbuscular mycorrhiza symbiosis in viticulture: a review. Agron. Sustain. Dev..

[7] Gilbert JA, van der Lelie D, Zarraonaindia I (2014). Microbial terroir for wine grapes. Proc. Natl. Acad. Sci..

[8] Hodge A, Helgason T, Fitter AH (2010). Nutritional ecology of arbuscular mycorrhizal fungi. Fungal Ecol..

[9] Bona, E. *et al*. Proteomic analysis as a tool for investigating arsenic stress in *Pteris vittata* roots colonized or not by arbuscular mycorrhizal symbiosis. *J. Proteomics* **74**, (2011). 10.1016/j.jprot.2011.03.027 21457805

[10] Lingua, G. *et al*. Effects of heavy metals and arbuscular mycorrhiza on the leaf proteome of a selected poplar clone: A time course analysis. *PLoS One* **7**, (2012). 10.1371/journal.pone.0038662 PMC3383689 22761694

[11] Degola, F. *et al*. The symbiosis between *Nicotiana tabacum* and the endomycorrhizal fungus *Funneliformis mosseae* increases the plant glutathione level and decreases leaf cadmium and root arsenic contents. *Plant Physiol. Biochem*. **92**, (2015). 10.1016/j.plaphy.2015.04.001 25900420

[12] Bona, E. *et al*. AM fungi and PGP pseudomonads increase flowering, fruit production, and vitamin content in strawberry grown at low nitrogen and phosphorus levels. *Mycorrhiza* **25**, (2015). 10.1007/s00572-014-0599-y 25169060

[13] Bona E (2017). Arbuscular mycorrhizal fungi and plant growth-promoting pseudomonads improve yield, quality and nutritional value of tomato: a field study. Mycorrhiza.

[14] Berta, G. *et al*. Maize development and grain quality are differentially affected by mycorrhizal fungi and a growth-promoting pseudomonad in the field. *Mycorrhiza* **24**, (2014). 10.1007/s00572-013-0523-x 23995918

[15] Baslam M, Esteban R, García-Plazaola JI, Goicoechea N (2013). Effectiveness of arbuscular mycorrhizal fungi (AMF) for inducing the accumulation of major carotenoids, chlorophylls and tocopherol in green and red leaf lettuces. Appl. Microbiol. Biotechnol..

[16] Bona, E. *et al*. Combined bacterial and mycorrhizal inocula improve tomato quality at reduced fertilization. *Sci. Hortic. (Amsterdam)*. **234**, (2018).

[17] Todeschini V (2018). Impact of Beneficial Microorganisms on Strawberry Growth, Fruit Production, Nutritional Quality, and Volatilome. Front. Plant Sci..

[18] Wagg C, Bender SF, Widmer F, van der Heijden MGA (2014). Soil biodiversity and soil community composition determine ecosystem multifunctionality. Proc. Natl. Acad. Sci..

[19] de Vries FT (2013). Soil food web properties explain ecosystem services across European land use systems. Proc. Natl. Acad. Sci..

[20] Gianinazzi S, Gollotte A, Binet M (2010). Agroecology: the key role of arbuscular mycorrhizas in ecosystem services. Mycorrhiza.

[21] Garcia JAL, Barbas C, Probanza A, Barrientos ML, Manero FJG (2001). Low molecular weight organic acids and fatty acids in root exudates of two Lupinus cultivars at flowering and fruiting stages. Phytochem. Anal..

[22] Holland TC, Bowen P, Bogdanoff C, Hart MM (2014). How distinct are arbuscular mycorrhizal fungal communities associating with grapevines?. Biol. Fertil. Soils.

[23] Linderman RG, Davis EA (2001). Comparative response of selected grapevine rootstocks and cultivars to inoculation with different mycorrhizal fungi. Am. J. Enol. Vitic..

[24] Schreiner, R. P. Spatial and temporal variation of roots, arbuscular mycorrhizal fungi, and plant and soil nutrients in a mature Pinot Noir (*Vitis vinifera* L.) vineyard in Oregon, USA. 219–234, 10.1007/s11104-005-4895-0 (2005).

[25] Schreiner RP, Scagel CF, Baham J (2006). Nutrient uptake and distribution in a mature ‘pinot noir’ vineyard. HortScience.

[26] Likar M, Hančević K, Radić T, Regvar M (2013). Distribution and diversity of arbuscular mycorrhizal fungi in grapevines from production vineyards along the eastern Adriatic coast. Mycorrhiza.

[27] Turrini, A. *et al*. Protective green cover enhances soil respiration and native mycorrhizal potential compared with soil tillage in a high-density olive orchard in a long term study. **116**, 70–78 (2017).

[28] Turrini, A., Agnolucci, M., Palla, M., Tomé, E. & Tagliavini, M. Species diversity and community composition of native arbuscular mycorrhizal fungi in apple roots are a ff ected by site and orchard management. **116**, 42–54 (2017).

[29] Paul Schreiner R (2007). Effects of native and nonnative arbuscular mycorrhizal fungi on growth and nutrient uptake of ‘Pinot noir’ (*Vitis vinifera* L.) in two soils with contrasting levels of phosphorus. Appl. Soil Ecol..

[30] Schreiner RP, Mihara KL (2009). The diversity of arbuscular mycorrhizal fungi amplified from grapevine roots (*Vitis vinifera* L.) in Oregon vineyards is seasonally stable and influenced by soil and vine age. Mycologia.

[31] Lumini E, Orgiazzi A, Borriello R, Bonfante P, Bianciotto V (2010). Disclosing arbuscular mycorrhizal fungal biodiversity in soil through a land-use gradient using a pyrosequencing approach. Environ. Microbiol..

[32] Balestrini R, Magurno F, Walker C, Lumini E, Bianciotto V (2010). Cohorts of arbuscular mycorrhizal fungi (AMF) in *Vitis vinifera*, a typical Mediterranean fruit crop. Environ. Microbiol. Rep..

[33] Likar M, Stres B, Rusjan D, Potisek M, Regvar M (2017). Ecological and conventional viticulture gives rise to distinct fungal and bacterial microbial communities in vineyard soils. Appl. Soil Ecol..

[34] Zaller, J. G. *et al*. Herbicides in vineyards reduce grapevine root mycorrhization and alter soil microorganisms and the nutrient composition in grapevine roots, leaves, xylem sap and grape juice. 23215–23226 (2018). 10.1007/s11356-018-2422-3 PMC6096560 29862481

[35] Novello, G. *et al*. The rhizosphere bacterial microbiota of *Vitis vinifera* cv. Pinot Noir in an integrated pest management vineyard. *Front. Microbiol. ***8**, 1–11 (2017). 10.3389/fmicb.2017.01528 PMC5557794 28855895

[36] ISTAT, Istituto Superiore di Statistica. (2018).

[37] Lindahl BD (2013). Methods Fungal community analysis by high-throughput sequencing of amplified markers – a user’s guide. New Phytol..

[38] Hart M (2015). Inoculation with arbuscular mycorrhizal fungi improves the nutritional value of tomatoes. Mycorrhiza.

[39] Holland TC (2016). Evaluating the diversity of soil microbial communities in vineyards relative to adjacent native ecosystems. Appl. Soil Ecol..

[40] Doherty, J. H., Harris, C., Hartley, L. & the Ecological Society of America. TIEE is a project of the Education and Human Resources Committee of the Ecological Society of America. *Teach. Issues Exp. Ecol*. **7**, (2011).

[41] Dumbrell AJ (2011). Distinct seasonal assemblages of arbuscular mycorrhizal fungi revealed by massively parallel pyrosequencing. New Phytol..

[42] Bouamri R, Dalpé Y, Serrhini MN (2014). Effect of seasonal variation on arbuscular mycorrhizal fungi associated with date palm. Emirates J. Food Agric..

[43] Vargas-Gastélum L (2015). Impact of seasonal changes on fungal diversity of a semi-arid ecosystem revealed by 454 pyrosequencing. FEMS Microbiol. Ecol..

[44] Cesaro, P. *et al*. Preferential colonization of *Solanum tuberosum* L. roots by the fungus *Glomus intraradices* in arable soil of a potato farming area. *Appl. Environ. Microbiol.* **74**, 5776–5783 (2008). 10.1128/AEM.00719-08 PMC2547043 18676711

[45] Berruti, A. *et al*. Arbuscular mycorrhizal fungi and their value for ecosystem management. *Biodivers. - Dyn. Balanc. planet* 159–191, dx.doi.org/10.5772/58231 (2014).

[46] Schreiner RP (2003). Mycorrhizal colonization of grapevine rootstocks under field conditions. Am. J. Enol. Vitic..

[47] Bona, E. *et al*. Metaproteomic characterization of the *Vitis vinifera* rhizosphere. **95**, 1–16 (2019). 10.1093/femsec/fiy204 30307579

[48] Trouvelot, A., Kough, J. L. & Gianinazzi-Pearson, V. Mesure du taux de mycorrhization VA d’un système radiculaire. Recherche de méthodes d’estimation ayant une signification functionelle. in *Mycorrhizae: physiology and genetics* 217–221 (1986).

[49] Farmer MJ (2007). Molecular monitoring of field-inoculated AMF to evaluate persistence in sweet potato crops in China. Appl. Soil Ecol..

[50] Ewing B, Hillier L, Wendl M, Green P (1998). Base-calling of automated sequencer traces usingPhred. I. Accuracy assessment. Genome Res..

[51] Krüger M, Krüger C, Walker C, Stockinger H, Schüßler A (2012). Phylogenetic reference data for systematics and phylotaxonomy of arbuscular: Discovery Service for University of Essex. New Phytol..

[52] Altschul SF (1997). Gapped BLAST and PSI-BLAST: a new generation of protein database search programs. Nucleic Acid Res..

[53] R Core Team. R: A language and environment for statistical computing. R Foundation for Statistical Computing, Vienna, Austria. (2018).

[54] Berlanas, C. *et al*. The fungal and bacterial rhizosphere microbiome associated with grapevine rootstock genotypes in mature and young vineyards. *Front. Microbiol.* **10**, 1–16 (2019). 10.3389/fmicb.2019.01142 PMC6538693 31178845

[55] Sergaki, C., Lagunas, B., Lidbury, I., Gifford, M. L. & Schäfer, P. Challenges and approaches in microbiome. *Research: From Fundamental to Applied.* **9**, 1–12 (2018). 10.3389/fpls.2018.01205 PMC6107787 30174681

[56] McMurdie, P. J. & Holmes, S. Phyloseq: An R package for reproducible interactive analysis and graphics of microbiome census data. *PLoS One* **8**, (2013). 10.1371/journal.pone.0061217 PMC3632530 23630581

[57] Foster, Z. S. L., Sharpton, T. J. & Gru, N. J. Metacoder: An R package for visualization and manipulation of community taxonomic diversity data. 1–15, 10.5281/zenodo.158228 (2017). 10.1371/journal.pcbi.1005404 PMC5340466 28222096

