# Supplementary material for: AMF communities associated to Vitis vinifera in an Italian vineyard subjected to integrated pest management at two different phenological stages
Source: Sci Rep. 2020 Jun 8;10:9197. doi: 10.1038/s41598-020-66067-w (PMC7280190; doi:10.1038/s41598-020-66067-w)
Supplement: Supplementary file 1 — Supplementary information. [file 41598_2020_66067_MOESM1_ESM.pdf]

AMF communities associated to *Vitis vinifera* in an Italian vineyard subjected to integrated pest management at two different phenological stages

Massa N.[1], Bona E.[2], Novello G.[1], Todeschini V.[2], Boatti L.[3], Mignone F.[1,3], Gamalero E.[1], Lingua G.[1], Berta G.[1], Cesaro P.[1*]

[1] Università del Piemonte Orientale, Dipartimento di Scienze e Innovazione Tecnologica, Viale T. Michel 11, Alessandria 15121, Italy

[2] Università del Piemonte Orientale, Dipartimento di Scienze e Innovazione Tecnologica, Piazza San Eusebio 5, 13100 Vercelli, Italy

[3] SmartSeq s.r.l., spin-off of the Università del Piemonte Orientale, Viale T. Michel 11, Alessandria 15121, Italy

*** Corresponding author:**

Dipartimento di Scienze e Innovazione Tecnologica, Università del Piemonte Orientale,

Viale T. Michel 11, Alessandria 15121, Italy

Tel: 0131 360 244

Fax: 0131 360 243

E-mail: patrizia.cesaro@uniupo.it

Supplementary Information

Figure S1

Rarefaction curves. Rarefaction curves for Bulk soil (Bs) and soil associated with the roots of *V. vinifera* cv. Pinot Noir (Rs) at the two sampling times 1S (first sampling, in May during flowering time) and 2S (second sampling, in July during fruiting time).

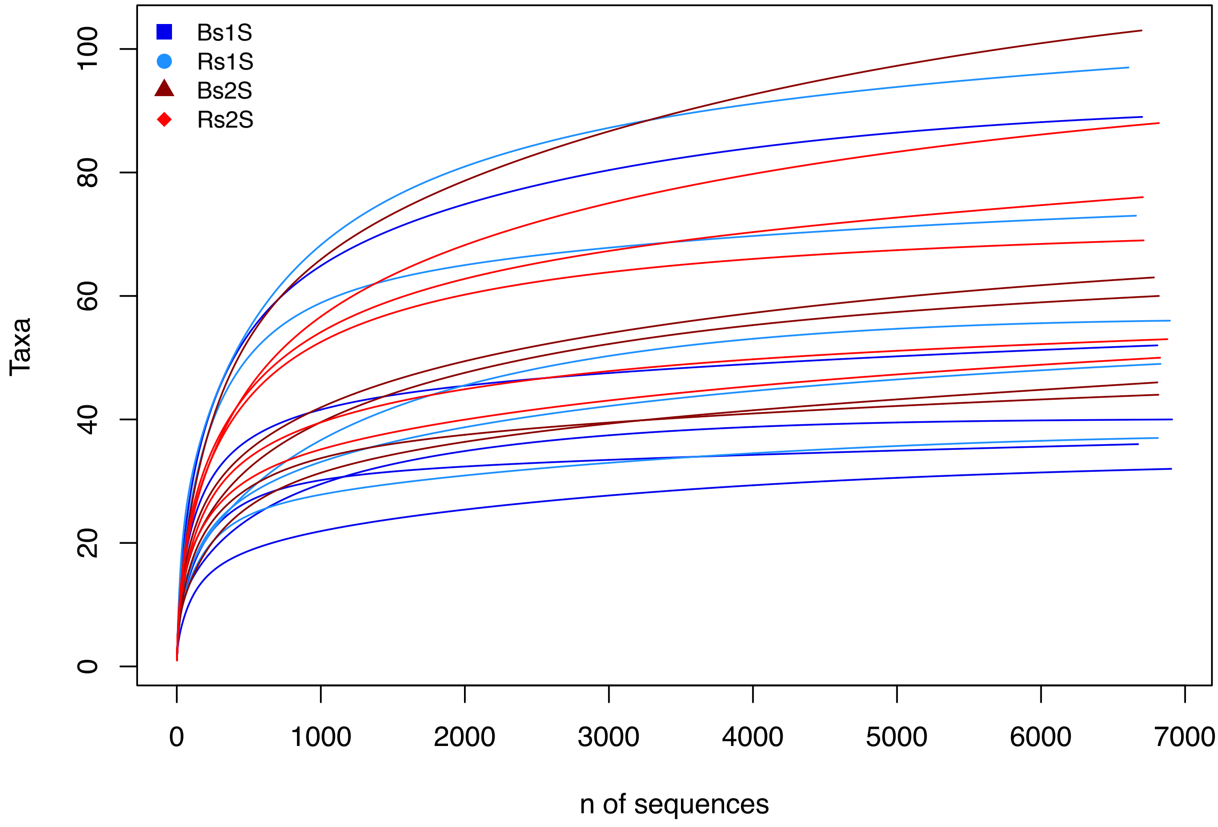

Figure S2. Climatic data. A) Mean temperature (°C), B) Mean humidity (%) and C) total rainfall (mm) in Carpeneto (AL) during the year 2014. Metereological data have been kindly provided by ARPA Piemonte.

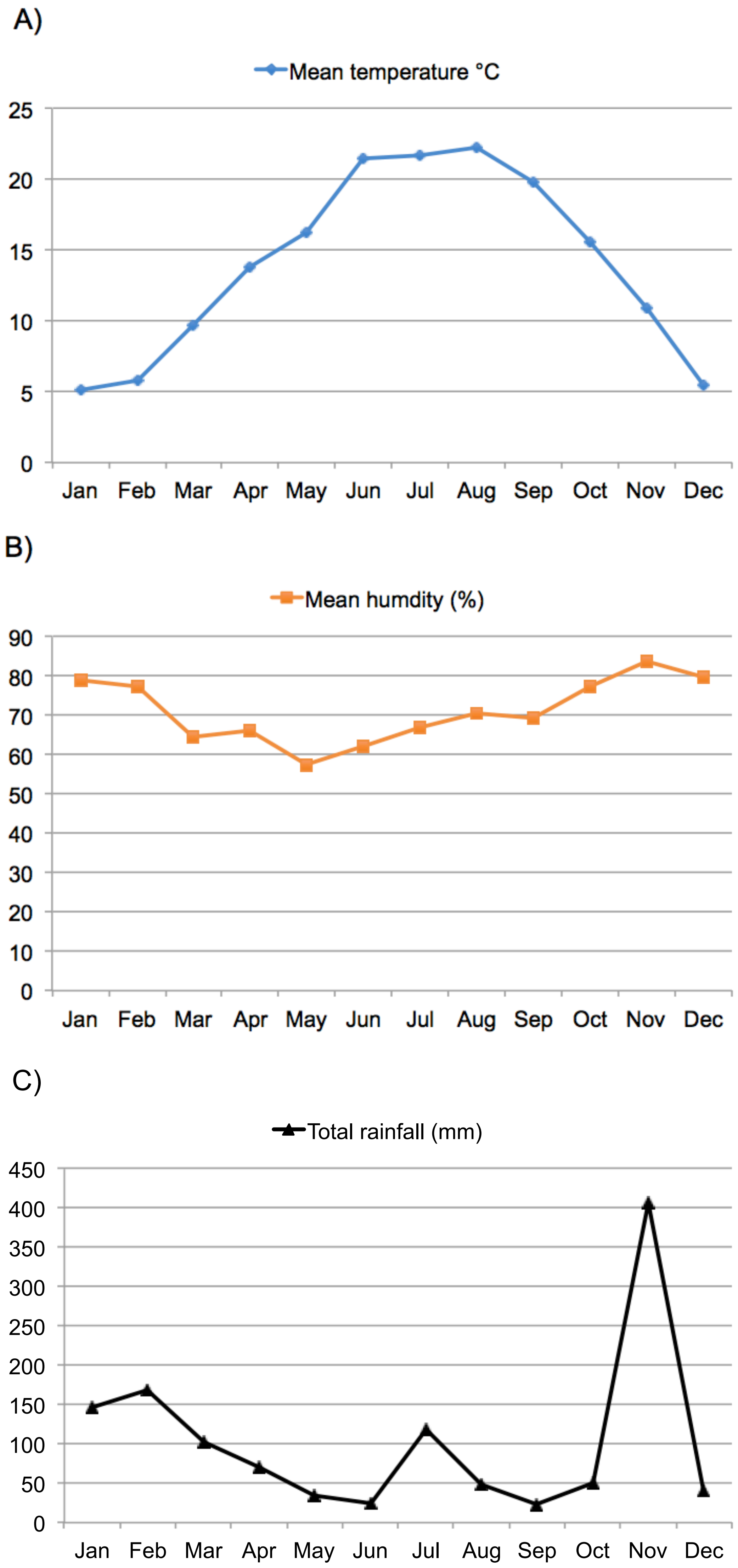
A)
Mean temperature °C
25
20
15
10
5
0
Jan Feb Mar Apr May Jun Jul Aug Sep Oct Nov Dec
B)
Mean humdity (%)
90
80
70
60
50
40
30
20
10
0
Jan Feb Mar Apr May Jun Jul Aug Sep Oct Nov Dec
C)
Total rainfall (mm)
450
400
350
300
250
200
150
100
50
0
Jan Feb Mar Apr May Jun Jul Aug Sep Oct Nov Dec

Table S1. List of IDs for "known" taxa identified by LSU rDNA database.

Taxa ID	Identification by LSU rDNA database	Description
AF145743	*Glomus geosporum* strain BEG 106 large subunit ribosomal RNA gene, partial sequence - *Funneliformis geosporum*	*Funneliformis geosporum*
AJ510241	*Glomus geosporum* partial 26S rRNA gene, isolate BEG11 - *Funneliformis geosporum*	*Funneliformis geosporum*
AJ854588	*Glomus* sp. MUCL 43203 28S rRNA gene, strain MUCL 43203, clone 5 - *Rhizophagus* sp. MUCL 43203	*Rhizophagus* sp.
AJ854601	*Glomus* sp. MUCL 43205 28S rRNA gene, strain MUCL 43205, clone 1 - *Glomus* sp. MUCL 43205	*Glomus* sp.
AJ854602	*Glomus* sp. MUCL 43205 28S rRNA gene, strain MUCL 43205, clone 2 - *Glomus* sp. MUCL 43205	*Glomus* sp.
AJ854606	*Glomus* sp. MUCL 43205 28S rRNA gene, strain MUCL 43205, clone 6 - *Glomus* sp. MUCL 43205	*Glomus* sp.
AJ854609	*Glomus* sp. MUCL 43205 28S rRNA gene, strain MUCL 43205, clone 9 - *Glomus* sp. MUCL 43205	*Glomus* sp.
AJ854611	*Glomus sp. MUCL 43205 28S rRNA gene, strain MUCL 43205, clone 11 - Glomus* sp. MUCL 43205	*Glomus* sp.
AJ854616	*Glomus* sp. MUCL 43206 28S rRNA gene, strain MUCL 43206, clone 4 - *Glomus* sp. MUCL 43206	*Glomus* sp.
AJ854618	*Glomus sp. MUCL 43206 28S rRNA gene, strain MUCL 43206, clone 6 - Glomus* sp. MUCL 43206	*Glomus* sp.
AJ854619	*Glomus sp. MUCL 43206 28S rRNA gene, strain MUCL 43206, clone 7 - Glomus* sp. MUCL 43206	*Glomus* sp.
AJ854621	*Glomus sp. MUCL 43206 28S rRNA gene, strain MUCL 43206, clone 9 - Glomus* sp. MUCL 43206	*Glomus* sp.
AJ854631	*Glomus* sp. MUCL 43207 28S rRNA gene, strain MUCL 43207, clone 9 - *Glomus* sp. MUCL 43207	*Glomus* sp.
AJ972457	*Glomus diaphanum partial 18S rRNA gene, ITS1, 5.8S rRNA gene, ITS2 and partial 28S rRNA gene, clone 1.2 - Glomus diaphanus*	*Glomus diaphanus*
AY639205	*Rhizophagus* cf. intraradices HG-2010 clone 34.1 28S large subunit ribosomal RNA gene, partial sequence - *Rhizophagus* cf. *intraradices* HG-2010	*Rhizophagus intraradices*
AY639208	*Rhizophagus* cf. intraradices HG-2010 clone 42.2 28S large subunit ribosomal RNA gene, partial sequence - *Rhizophagus* cf. *intraradices* HG-2010	*Rhizophagus intraradices*
AY639214	*Rhizophagus* cf. intraradices HG-2010 isolate 57 clone 57.7.1 28S large subunit ribosomal RNA gene, partial sequence - *Rhizophagus* cf. *intraradices* HG-2010	*Rhizophagus intraradices*
AY639216	*Rhizophagus* cf. intraradices HG-2010 isolate 58 clone 58.1.4 28S large subunit ribosomal RNA gene, partial sequence - *Rhizophagus* cf. *intraradices* HG-2010	*Rhizophagus intraradices*
AY639218	*Rhizophagus* cf. intraradices HG-2010 isolate 107 clone 107.3.1 28S large subunit ribosomal RNA gene, partial sequence - *Rhizophagus* cf. *intraradices* HG-2010	*Rhizophagus intraradices*
AY639219	*Rhizophagus* cf. intraradices HG-2010 isolate 107 clone 107.3.3 28S large subunit ribosomal RNA gene, partial sequence - *Rhizophagus* cf. *intraradices* HG-2010	*Rhizophagus intraradices*
AY639294	*Rhizophagus cf. intraradices HG-2010 clone 28.1 28S large subunit ribosomal RNA gene, partial sequence - Rhizophagus* cf. *intraradices* HG-2010	*Rhizophagus intraradices*
AY639299	*Rhizophagus cf. intraradices HG-2010 isolate 57 clone 57.6.3 28S large subunit ribosomal RNA gene, partial sequence - Rhizophagus* cf. *intraradices*	*Rhizophagus intraradices*
AY639301	*Rhizophagus cf. intraradices HG-2010 isolate 57 clone 57.7.3 28S large subunit ribosomal RNA gene, partial sequence - Rhizophagus* cf. *intraradices* HG-2010	*Rhizophagus intraradices*
DQ469114	*Glomus* cf. *diaphanum* 589 clone 589.2 28S large subunit ribosomal RNA gene, partial sequence - *Glomus* cf. *diaphanum* 589	*Glomus diaphanus*
DQ469115	*Glomus cf. diaphanum 589 clone 589.3 28S large subunit ribosomal RNA gene, partial sequence - Glomus* cf. *diaphanum* 589	*Glomus diaphanus*
DQ469120	*Rhizophagus* cf. *intraradices* HG-2010 isolate iv1 clone iv1.5 28S large subunit ribosomal RNA gene, partial sequence - *Rhizophagus* cf. *intraradices* HG-2010	*Rhizophagus intraradices*
DQ469123	*Rhizophagus* cf. *intraradices* HG-2010 isolate iv1 clone iv1.9 28S large subunit ribosomal RNA gene, partial sequence - *Rhizophagus* cf. *intraradices* HG-2010	*Rhizophagus intraradices*
Fi1cons#33	Uncultured *Rhizophagus* partial 28S RRNA gene , OTU AMF_C019Rhirr	*Rhizophagus s* p.
FR750095	*Rhizophagus sp. MUCL 43208 18S rRNA gene (partial), ITS1, 5.8S rRNA gene, ITS2 and 28S rRNA gene (partial), isolate MUCL43208, clone pHS055-47*<br>*Rhizophagus sp. MUCL 43208 - Rhizophagus* sp. MUCL 43208	*Rhizophagus* sp.
FR750111	*Rhizophagus* cf. *irregularis* MUCL 43205 18S rRNA gene (partial), ITS1, 5.8S rRNA gene, ITS2 and 28S rRNA gene (partial), isolate MUCL43205, clone pHS058-15 - *Rhizophagus* cf. *irregularis* MUCL 43205	*Rhizophagus irregularis*

Taxa ID	Identification by LSU rDNA database	Description
FR750116	*Rhizophagus cf. irregularis MUCL 43205 18S rRNA gene (partial), ITS1, 5.8S rRNA gene, ITS2 and 28S rRNA gene (partial), isolate MUCL43205, clone pHS058-6 - Rhizophagus* cf. *irregularis* MUCL 43205	*Rhizophagus irregularis*
FR750190	*Rhizophagus irregularis* 18S rRNA gene (partial), ITS1, 5.8S rRNA gene, ITS2 and 28S rRNA gene (partial), isolate Att857-12, clone pMK100-7 - *Rhizophagus irregularis*	*Rhizophagus irregularis*
FR750198	*Rhizophagus irregularis* 18S rRNA gene (partial), ITS1, 5.8S rRNA gene, ITS2 and 28S rRNA gene (partial), isolate from Poland (trap culture), clone pMK108-7 - *Rhizophagus irregularis*	*Rhizophagus irregularis*
FR750530	*Glomus macrocarpum* 18S rRNA gene (partial), ITS1, 5.8S rRNA gene, ITS2 and 28S rRNA gene (partial), specimen voucher W5288, clone pHS093-49 - *Glomus macrocarpum*	*Glomus macrocarpum*
Gintra691BEG144		*Glomus intraradices*
Gintx99640BEG141		*Glomus intraradices*
HE794038	*Glomus sp. PB genomic DNA containing 18S rRNA gene, ITS1, 5.8S rRNA gene, ITS2 and 28S rRNA gene, strain PB, clone pb1*<br>*Glomus sp. PB - Glomus* sp. PB	*Glomus* sp.
HF548860	*Septoglomus viscosum* genomic DNA containing 18S rRNA gene, ITS1, 5.8S rRNA gene, ITS2, 28S rRNA gene, strain BEG27, isolate Att179-15, clone pDS001-32 - *Septoglomus viscosum*	*Septoglomus viscosum*
HF968919	*Rhizophagus irregularis genomic DNA containing 18S rRNA gene, ITS1, 5.8S rRNA gene, ITS2 and 28S rRNA gene, strain DAOM181602, isolate spore 1, clone EI2_3_8*<br>*Rhizophagus irregularis - Rhizophagus irregularis*	*Rhizophagus irregularis*
HF968920	*Rhizophagus irregularis* genomic DNA containing 18S rRNA gene, ITS1, 5.8S rRNA gene, ITS2 and 28S rRNA gene, strain DAOM181602, isolate spore 1, clone EI2_3_9 - *Rhizophagus irregularis*	*Rhizophagus irregularis*
HF968983	*Rhizophagus irregularis partial 28S rRNA gene, strain DAOM181602, isolate spore 3, clone EL2_7_75 - Rhizophagus irregularis*	*Rhizophagus irregularis*
HF968991	*Rhizophagus irregularis* partial 28S rRNA gene, strain DAOM181602, isolate spore 2, clone EL2_5_24 - *Rhizophagus irregularis*	*Rhizophagus irregularis*
HF968992	*Rhizophagus irregularis* partial 28S rRNA gene, strain DAOM181602, isolate spore 2, clone EL2_5_59 - *Rhizophagus irregularis*	*Rhizophagus irregularis*
HG969377	*Glomus invermaium* genomic DNA containing 18S rRNA gene, ITS1, 5.8S rRNA gene, ITS2 and 28S rRNA gene, isolate Att1646, clone ECU102P9 - *Rhizophagus invermaius*	*Rhizophagus invermaius*
HG969382	*Glomus invermaium* genomic DNA containing 18S rRNA gene, ITS1, 5.8S rRNA gene, ITS2 and 28S rRNA gene, isolate Att1646, clone ECU102P12 - *Rhizophagus invermaius*	*Rhizophagus invermaius*
HG969388	*Glomus invermaium* genomic DNA containing 18S rRNA gene, ITS1, 5.8S rRNA gene, ITS2 and 28S rRNA gene, isolate Att1646, clone ECU102P30 - *Rhizophagus invermaius*	*Rhizophagus invermaius*
HG969390	*Glomus invermaium* genomic DNA containing 18S rRNA gene, ITS1, 5.8S rRNA gene, ITS2 and 28S rRNA gene, isolate Att1646, clone ECU102P13 - *Rhizophagus invermaius*	*Rhizophagus invermaius*
HM625892	*Glomus intraradices* isolate intra6 28S ribosomal RNA gene, partial sequence - *Rhizophagus intraradices*	*Rhizophagus intraradices*
JF439109	*Glomus intraradices isolate B22-11 18S ribosomal RNA gene, partial sequence; internal transcribed spacer 1, 5.8S ribosomal RNA gene, and internal transcribed spacer 2, complete sequence; and 28S ribosomal RNA gene, …- Glomus intraradices* isolate B22-11	*Glomus intraradices*
JF439138	*Glomus aggregatum isolate S08-1-13 18S ribosomal RNA gene, partial sequence; internal transcribed spacer 1, 5.8S ribosomal RNA gene, and internal transcribed spacer 2, complete sequence; and 28S ribosomal RNA gene, … - Glomus aggregatum* isolate S08-1-13	*Glomus aggregatum*
JF439161	*Glomus sp. 9 SUN-2011 isolate 08_48_1 18S ribosomal RNA gene, partial sequence; internal transcribed spacer 1, 5.8S ribosomal RNA gene, and internal transcribed spacer 2, complete sequence; and 28S ribosomal … - Glomus* sp. 9 SUN-2011 isolate 08_48_1	*Glomus* sp.
JF439162	*Glomus* sp. 7 SUN-2011 isolate 08_48_2	*Glomus* sp.
JF439169	*Glomus intraradices* isolate 08_62_4 18S ribosomal RNA gene, partial sequence; internal transcribed spacer 1, 5.8S ribosomal RNA gene, and internal transcribed spacer 2, complete sequence; and 28S ribosomal RNA gene, … - *Rhizophagus intraradices*	*Rhizophagus intraradices*
JF439175	UNVERIFIED: Glomus sp. 20 SUN-2011 isolate 08_48_10 18S ribosomal RNA gene, partial sequence; internal transcribed spacer 1, 5.8S ribosomal RNA gene, and internal transcribed spacer 2, complete sequence; and 28S … - UNVERIFIED: *Glomus* sp. 20 SUN-2011	*Glomus* sp.
JF439190	*Glomus aggregatum isolate 08_6_1 18S ribosomal RNA gene, partial sequence; internal transcribed spacer 1, 5.8S ribosomal RNA gene, and internal transcribed spacer 2, complete sequence; and 28S ribosomal RNA gene, … - Glomus aggregatum* isolate 08_6_1	*Glomus aggregatum*

Taxa ID	Identification by LSU rDNA database	Description
JN417514	*Rhizophagus irregularis* clone 1.12 18S ribosomal RNA gene, partial sequence; internal transcribed spacer 1, 5.8S ribosomal RNA gene, and internal transcribed spacer 2, complete sequence; and 28S ribosomal RNA gene, … - *Rhizophagus irregularis*	*Rhizophagus irregularis*
JN417518	*Rhizophagus irregularis* clone 2.4 18S ribosomal RNA gene, partial sequence; internal transcribed spacer 1, 5.8S ribosomal RNA gene, and internal transcribed spacer 2, complete sequence; and 28S ribosomal RNA gene, … - *Rhizophagus irregularis*	*Rhizophagus irregularis*
JQ048883	*Funneliformis caledonium* strain BEG20 clone Hsp42-16 28S ribosomal RNA (LSU) gene, partial sequence - *Funneliformis caledonium*	*Funneliformis caledonium*
JQ048895	*Funneliformis geosporum* strain BEG11 clone Hsp40-13b 28S ribosomal RNA (LSU) gene, partial sequence - *Funneliformis geosporum*	*Funneliformis geosporum*
KC182036	*Septoglomus viscosum clone HSp82-2 18S ribosomal RNA gene, partial sequence; internal transcribed spacer 1, 5.8S ribosomal RNA gene, and internal transcribed spacer 2, complete sequence; and 28S ribosomal RNA gene, … - Septoglomus viscosum* clone HSp82-2	*Septoglomus viscosum*
KC182037	*Septoglomus viscosum clone HSp82-9 18S ribosomal RNA gene, partial sequence; internal transcribed spacer 1, 5.8S ribosomal RNA gene, and internal transcribed spacer 2, complete sequence; and 28S ribosomal RNA gene, … - Septoglomus viscosum* clone HSp82-9	*Septoglomus viscosum*
KC182043	*Glomus macrocarpum* clone HSp135-1 18S ribosomal RNA gene, partial sequence; internal transcribed spacer 1, 5.8S ribosomal RNA gene, and internal transcribed spacer 2, complete sequence; and 28S ribosomal RNA gene, … - *Glomus macrocarpum*	*Glomus macrocarpum*
KF060306	*Septoglomus africanum* isolate 207-4 18S ribosomal RNA gene, partial sequence; internal transcribed spacer 1, 5.8S ribosomal RNA gene, and internal transcribed spacer 2, complete sequence; and 28S ribosomal RNA gene, … - *Septoglomus africanum*	*Septoglomus africanum*
KF060322	*Septoglomus jasnowskae* isolate 206-5 18S ribosomal RNA gene, partial sequence; internal transcribed spacer 1, 5.8S ribosomal RNA gene, and internal transcribed spacer 2, complete sequence; and 28S ribosomal RNA gene, … - *Septoglomus jasnowskae*	*Septoglomus jasnowskae*
KF154775	*Septoglomus xanthium* isolate 6 18S ribosomal RNA gene, partial sequence; internal transcribed spacer 1, 5.8S ribosomal RNA gene, and internal transcribed spacer 2, complete sequence; and 28S ribosomal RNA gene, … - *Septoglomus xanthium*	*Septoglomus xanthium*
KF836904	*Glomus sp. 7 ZHNL-2013a isolate D1_23 18S ribosomal RNA gene, partial sequence; internal transcribed spacer 1, 5.8S ribosomal RNA gene, and internal transcribed spacer 2, complete sequence; and 28S ribosomal … - Glomus* sp. 7 ZHNL-2013a isolate D1_23	*Glomus* sp.
KF836937	*Glomus* sp. 5 ZHNL-2013 isolate E2_21 18S ribosomal RNA gene, partial sequence; internal transcribed spacer 1, 5.8S ribosomal RNA gene, and internal transcribed spacer 2, complete sequence; and 28S ribosomal … - *Glomus* sp. 5 ZHNL-2013	*Glomus* sp.
KF836948	Glomus sp. 7 ZHNL-2013b isolate E4_5 18S ribosomal RNA gene, partial sequence; internal transcribed spacer 1, 5.8S ribosomal RNA gene, and internal transcribed spacer 2, complete sequence; and 28S ribosomal … - Glomus sp. 7 ZHNL-2013b	*Glomus* sp.
KF836962	*Rhizophagus intraradices* isolate E5_18 18S ribosomal RNA gene, partial sequence; internal transcribed spacer 1, 5.8S ribosomal RNA gene, and internal transcribed spacer 2, complete sequence; and 28S ribosomal RNA gene, … - *Rhizophagus intraradices*	*Rhizophagus intraradices*
KP191484	*Claroideoglomus drummondii* isolate 120-21 18S ribosomal RNA gene, partial sequence; internal transcribed spacer 1, 5.8S ribosomal RNA gene, and internal transcribed spacer 2, complete sequence; and 28S ribosomal RNA gene, … - *Claroideoglomus drummondii*	*Claroideoglomus drummondii*
MTTD1seq	Rhizophagus irregularis	*Rhizophagus irregularis*

Table S2. Identification of *de novo* taxa by BLAST analysis against NCBI database. In the last column, culturable taxa are highlighted in green.

query	subject	Bs1S		Bs2S		Rs1S		Rs2S		Description
		% coverage	bp query	% coverage	bp query	% coverage	bp query	% coverage	bp query	
de novo _1	HE858375.1	98.36	732	98.36	732	98.36	732	98.36	732	Uncultured *Glomus* partial 28S rRNA gene, clone WW1-6
de novo _10023	FM865550.1	-	-	-	-	96.59	557	-	-	*Glomus* sp. Att1192-44 18S rRNA gene (partial), ITS1, 5.8S rRNA gene, ITS2 and 28S rRNA gene (partial), isolate Att1192-44, clone pHS059-1
de novo _10028	HF968920.1	-	-	-	-	96.76	710	-	-	*Rhizophagus irregularis* genomic DNA containing 18S rRNA gene, ITS1, 5.8S rRNA gene, ITS2 and 28S rRNA gene, strain DAOM181602, isolate spore 1, clone EI2_3_9
de novo _10048	FR871359.1	-	-	-	-	94.33	723	-	-	Uncultured *Glomus* partial 28S rRNA gene, clone BRAS4-24
de novo _10067	AJ854612.1	-	-	-	-	96.53	720	96.53	720	*Glomus* sp. MUCL 43205 28S rRNA gene, strain MUCL 43205, clone 12
de novo _10069	FR750081.1	-	-	-	-	88.86	718	-	-	*Rhizophagus irregularis* 18S rRNA gene (partial), ITS1, 5.8S rRNA gene, ITS2 and 28S rRNA gene (partial), isolate MUCL43195, clone pHS037-5
de novo _10098	AJ854611.1	-	-	-	-	-	-	90.92	716	*Glomus* sp. MUCL 43205 28S rRNA gene, strain MUCL 43205, clone 11
de novo _10217	FR871359.1	-	-	-	-	92.72	728	-	-	Uncultured *Glomus* partial 28S rRNA gene, clone BRAS4-24
de novo _10288	HQ243142.1	-	-	-	-	-	-	95.82	646	Uncultured *Glomus* clone ZHwq2-350 18S ribosomal RNA gene, partial sequence; internal transcribed spacer 1, 5.8S ribosomal RNA gene, and internal transcribed spacer 2, complete sequence; and 28S
de novo _10371	KC411151.1	-	-	-	-	92.68	710	-	-	Uncultured *Glomerales* clone H19_35 large subunit ribosomal RNA gene, partial sequence
de novo _10388	FR871386.1	-	-	-	-	-	-	96.82	724	Uncultured *Glomus* partial 28S rRNA gene, clone BROS3-13
de novo _10452	KC411151.1	-	-	-	-	92.47	717	-	-	Uncultured *Glomerales* clone H19_35 large subunit ribosomal RNA gene, partial sequence
de novo _10455	KC411151.1	-	-	-	-	91.77	717	-	-	Uncultured *Glomerales* clone H19_35 large subunit ribosomal RNA gene, partial sequence
de novo _10505	KC182037.1	-	-	-	-	96.31	731	96.31	731	*Septoglomus viscosum* clone HSp82-9 18S ribosomal RNA gene, partial sequence; internal transcribed spacer 1, 5.8S ribosomal RNA gene, and internal transcribed spacer 2, complete sequence; and 28S
de novo _10559	KC182037.1	-	-	-	-	-	-	97.1	725	*Septoglomus viscosum* clone HSp82-9 18S ribosomal RNA gene, partial sequence; internal transcribed spacer 1, 5.8S ribosomal RNA gene, and internal transcribed spacer 2, complete sequence; and 28S
de novo _10699	KC411151.1	-	-	-	-	92.05	742	-	-	Uncultured *Glomerales* clone H19_35 large subunit ribosomal RNA gene, partial sequence
de novo _1071	HE775295.1	98.08	677	98.08	677	-	-	-	-	Uncultured *Rhizophagus* 18S rRNA gene (partial), ITS1, 5.8S rRNA gene, ITS2 and 28S rRNA gene (partial), tissue library 32e, clone library KRU, clone 17
de novo _10779	KC411217.1	-	-	-	-	-	-	90.23	686	Uncultured *Glomerales* clone B04_50 large subunit ribosomal RNA gene, partial sequence
de novo _1092	HG425889.1	97.86	702	-	-	-	-	-	-	Uncultured *Rhizophagus* genomic DNA containing 18S rRNA gene, ITS1, 5.8S rRNA gene, ITS2 and 28S rRNA gene, clone Olse K-X19
de novo _10993	FR871389.1	-	-	-	-	96.11	746	96.11	746	Uncultured *Glomus* partial 28S rRNA gene, clone BRAS3-30
de novo _11007	AJ854597.1	-	-	-	-	-	-	90.75	724	*Glomus* sp. MUCL 43204 28S rRNA gene, strain MUCL 43204, clone 5
de novo _11018	AJ854611.1	-	-	-	-	-	-	89.25	716	*Glomus* sp. MUCL 43205 28S rRNA gene, strain MUCL 43205, clone 11
de novo _11021	FR871386.1	-	-	-	-	-	-	94.79	710	Uncultured *Glomus* partial 28S rRNA gene, clone BROS3-13
de novo _11040	FR871344.1	-	-	-	-	-	-	97.12	728	Uncultured *Glomus* partial 28S rRNA gene, clone BROR3-28
de novo _11042	FR871386.1	-	-	-	-	-	-	94.08	709	Uncultured *Glomus* partial 28S rRNA gene, clone BROS3-13
de novo _11056	AJ854611.1	-	-	-	-	-	-	96.41	725	*Glomus* sp. MUCL 43205 28S rRNA gene, strain MUCL 43205, clone 11
de novo _11066	FR871344.1	-	-	-	-	97.11	658	97.11	658	Uncultured *Glomus* partial 28S rRNA gene, clone BROR3-28
de novo _11166	FR871386.1	-	-	-	-	-	-	93.1	710	Uncultured *Glomus* partial 28S rRNA gene, clone BROS3-13
de novo _1131	HE775295.1	97.79	678	97.79	678	-	-	-	-	Uncultured *Rhizophagus* 18S rRNA gene (partial), ITS1, 5.8S rRNA gene, ITS2 and 28S rRNA gene (partial), tissue library 32e, clone library KRU, clone 17

query	subject	Bs1S		Bs2S		Rs1S		Rs2S		Description
		% coverage	bp query	% coverage	bp query	% coverage	bp query	% coverage	bp query	
de novo _1134	HG425888.1	-	-	97.44	703	-	-	-	-	Uncultured *Rhizophagus* genomic DNA containing 18S rRNA gene, ITS1, 5.8S rRNA gene, ITS2 and 28S rRNA gene, clone JavorIX1
de novo _11388	KC411100.1	-	-	-	-	-	-	94.97	696	Uncultured *Glomerales* clone H17_30 large subunit ribosomal RNA gene, partial sequence
de novo _11395	HQ242938.1	-	-	-	-	92.31	689	-	-	Uncultured *Glomus* clone ZHwq2-146 18S ribosomal RNA gene, partial sequence; internal transcribed spacer 1, 5.8S ribosomal RNA gene, and internal transcribed spacer 2, complete sequence; and 28S ribosomal RNA gene, partial sequence
de novo _1185	KC411135.1	-	-	90.03	722	90.03	722	90.03	722	Uncultured *Glomerales* clone H19_14 large subunit ribosomal RNA gene, partial sequence
de novo _11874	HE858375.1	-	-	-	-	98.6	712	98.6	712	Uncultured *Glomus* partial 28S rRNA gene, clone WW1-6
de novo _11875	HE775307.1	-	-	-	-	-	-	98.27	577	Uncultured *Rhizophagus* 18S rRNA gene (partial), ITS1, 5.8S rRNA gene, ITS2 and 28S rRNA gene (partial), tissue library 2/1R-2/1-4/1, clone library KRU, clone 13
de novo _11892	HE858375.1	-	-	-	-	-	-	98.59	711	Uncultured *Glomus* partial 28S rRNA gene, clone WW1-6
de novo _11903	HE858375.1	-	-	-	-	98.73	711	-	-	Uncultured *Glomus* partial 28S rRNA gene, clone WW1-6
de novo _11906	KC411264.1	-	-	-	-	-	-	95.95	691	Uncultured *Glomerales* clone B08_45 large subunit ribosomal RNA gene, partial sequence
de novo _11988	HG969377.1	-	-	-	-	-	-	96.77	712	*Glomus invermaium* genomic DNA containing 18S rRNA gene, ITS1, 5.8S rRNA gene, ITS2 and 28S rRNA gene, isolate Att1646, clone ECU102P9
de novo _12	HE858375.1	98.09	733	98.09	733	98.09	733	98.09	733	Uncultured *Glomus* partial 28S rRNA gene, clone WW1-6
de novo _121	HE858375.1	-	-	-	-	99.15	708	-	-	Uncultured *Glomus* partial 28S rRNA gene, clone WW1-6
de novo _12430	HE858375.1	-	-	-	-	98.45	710	-	-	Uncultured *Glomus* partial 28S rRNA gene, clone WW1-6
de novo _12444	HE858375.1	-	-	-	-	97.36	720	-	-	Uncultured *Glomus* partial 28S rRNA gene, clone WW1-6
de novo _1245	KC410946.1	-	-	-	-	90.69	709	-	-	Uncultured *Glomerales* clone H3_24 large subunit ribosomal RNA gene, partial sequence
de novo _12983	HE858378.1	-	-	-	-	97.91	719	-	-	Uncultured *Glomus* partial 28S rRNA gene, clone WW2-5
de novo _13094	HE858378.1	-	-	-	-	97.31	707	-	-	Uncultured *Glomus* partial 28S rRNA gene, clone WW2-5
de novo _13229	KC411151.1	-	-	-	-	92.35	719	-	-	Uncultured *Glomerales* clone H19_35 large subunit ribosomal RNA gene, partial sequence
de novo _1446	HG425888.1	98.38	680	-	-	-	-	-	-	Uncultured *Rhizophagus* genomic DNA containing 18S rRNA gene, ITS1, 5.8S rRNA gene, ITS2 and 28S rRNA gene, clone JavorIX1
de novo _14551	JQ048924.1	-	-	-	-	-	-	92.7	740	*Glomus deserticola* strain BEG73 clone HSp30-8 28S ribosomal RNA (LSU) gene, partial sequence
de novo _14559	KC411100.1	-	-	-	-	-	-	94.32	687	Uncultured *Glomerales* clone H17_30 large subunit ribosomal RNA gene, partial sequence
de novo _14562	HE858378.1	-	-	-	-	-	-	98.31	712	Uncultured *Glomus* partial 28S rRNA gene, clone WW2-5
de novo _14564	KC411094.1	-	-	-	-	-	-	95.62	707	Uncultured *Glomerales* clone H17_24 large subunit ribosomal RNA gene, partial sequence
de novo _14611	HQ243142.1	-	-	-	-	-	-	94.45	649	Uncultured *Glomus* clone ZHwq2-350 18S ribosomal RNA gene, partial sequence; internal transcribed spacer 1, 5.8S ribosomal RNA gene, and internal transcribed spacer 2, complete sequence; and 28S ribosomal RNA gene, partial sequence
de novo _14640	KC411217.1	-	-	-	-	-	-	90.19	693	Uncultured *Glomerales* clone B04_50 large subunit ribosomal RNA gene, partial sequence
de novo _14670	HQ243020.1	-	-	-	-	-	-	94.59	684	Uncultured *Glomus* clone ZHwq2-228 18S ribosomal RNA gene, partial sequence; internal transcribed spacer 1, 5.8S ribosomal RNA gene, and internal transcribed spacer 2, complete sequence; and 28S ribosomal RNA gene, partial sequence
de novo _147	HE858375.1	-	-	-	-	-	-	99.15	710	Uncultured *Glomus* partial 28S rRNA gene, clone WW1-6
de novo _14766	HQ243142.1	-	-	-	-	-	-	95.29	594	Uncultured *Glomus* clone ZHwq2-350 18S ribosomal RNA gene, partial sequence; internal transcribed spacer 1, 5.8S ribosomal RNA gene, and internal transcribed spacer 2, complete sequence; and 28S ribosomal RNA gene, partial sequence

query	subject	Bs1S		Bs2S		Rs1S		Rs2S		Description
		% coverage	bp query	% coverage	bp query	% coverage	bp query	% coverage	bp query	
de novo _14777	HQ243020.1	-	-	-	-	-	-	95.1	673	Uncultured *Glomus* clone ZHwq2-228 18S ribosomal RNA gene, partial sequence; internal transcribed spacer 1, 5.8S ribosomal RNA gene, and internal transcribed spacer 2, complete sequence; and 28S ribosomal RNA gene, partial sequence
de novo _14868	KC411217.1	-	-	-	-	-	-	92.12	685	Uncultured *Glomerales* clone B04_50 large subunit ribosomal RNA gene, partial sequence
de novo _15	FR750055.1	92.5	707	-	-	92.5	707	92.5	707	*Claroideoglomus claroideum* 18S rRNA gene (partial), ITS1, 5.8S rRNA gene, ITS2 and 28S rRNA gene (partial), isolate Att1063-4, clone pCK085-1
de novo _1506	HE775295.1	98.12	691	98.12	691	-	-	-	-	Uncultured *Rhizophagus* 18S rRNA gene (partial), ITS1, 5.8S rRNA gene, ITS2 and 28S rRNA gene (partial), tissue library 32e, clone library KRU, clone 17
de novo _151	HE858375.1	-	-	-	-	98.87	711	98.87	711	Uncultured *Glomus* partial 28S rRNA gene, clone WW1-6
de novo _15710	HE858375.1	-	-	-	-	97.78	721	-	-	Uncultured *Glomus* partial 28S rRNA gene, clone WW1-6
de novo _16032	AM947928.1	-	-	-	-	97.19	676	-	-	Uncultured glomeromycete partial 25S rRNA gene, clone CSS-Ba5
de novo _16034	AM947928.1	-	-	-	-	97.63	676	-	-	Uncultured glomeromycete partial 25S rRNA gene, clone CSS-Ba5
de novo _16035	FJ461841.1	-	-	-	-	97.49	717	-	-	*Glomus macrocarpum* isolate MD124 25S ribosomal RNA gene, partial sequence
de novo _16036	AM947928.1	-	-	-	-	97.07	682	-	-	Uncultured glomeromycete partial 25S rRNA gene, clone CSS-Ba5
de novo _16039	AM947928.1	-	-	-	-	97.19	676	-	-	Uncultured glomeromycete partial 25S rRNA gene, clone CSS-Ba5
de novo _16042	AM947928.1	-	-	-	-	97.19	675	-	-	Uncultured glomeromycete partial 25S rRNA gene, clone CSS-Ba5
de novo _16053	HF970229.1	-	-	-	-	97.65	722	-	-	Uncultured Funneliformis genomic DNA containing 18S rRNA gene, ITS1, 5.8S rRNA gene, ITS2 and 28S rRNA gene, clone CS035-02
de novo _16054	AM947928.1	-	-	-	-	96.76	680	96.76	680	Uncultured glomeromycete partial 25S rRNA gene, clone CSS-Ba5
de novo _16060	AM947928.1	-	-	-	-	97.48	675	-	-	Uncultured glomeromycete partial 25S rRNA gene, clone CSS-Ba5
de novo _16065	KX758116.1	-	-	-	-	95.67	647	-	-	*Dominikia lithuanica* strain 331-2 small subunit ribosomal RNA gene, partial sequence; internal transcribed spacer 1, 5.8S ribosomal RNA gene, and internal transcribed spacer 2, complete sequence; and large subunit ribosomal RNA gene, partial sequence
de novo _16068	AM947928.1	-	-	-	-	96.89	676	-	-	Uncultured glomeromycete partial 25S rRNA gene, clone CSS-Ba5
de novo _16083	KX758116.1	-	-	-	-	95.68	717	-	-	*Dominikia lithuanica* strain 331-2 small subunit ribosomal RNA gene, partial sequence; internal transcribed spacer 1, 5.8S ribosomal RNA gene, and internal transcribed spacer 2, complete sequence; and large subunit ribosomal RNA gene, partial sequence
de novo _16094	KX758116.1	-	-	-	-	95.74	727	-	-	*Dominikia lithuanica* strain 331-2 small subunit ribosomal RNA gene, partial sequence; internal transcribed spacer 1, 5.8S ribosomal RNA gene, and internal transcribed spacer 2, complete sequence; and large subunit ribosomal RNA gene, partial sequence
de novo _16098	KX758116.1	-	-	-	-	95.64	711	-	-	*Dominikia lithuanica* strain 331-2 small subunit ribosomal RNA gene, partial sequence; internal transcribed spacer 1, 5.8S ribosomal RNA gene, and internal transcribed spacer 2, complete sequence; and large subunit ribosomal RNA gene, partial sequence
de novo _16101	KX758116.1	-	-	-	-	95.48	730	-	-	*Dominikia lithuanica* strain 331-2 small subunit ribosomal RNA gene, partial sequence; internal transcribed spacer 1, 5.8S ribosomal RNA gene, and internal transcribed spacer 2, complete sequence; and large subunit ribosomal RNA gene, partial sequence
de novo _16112	KX758116.1	-	-	-	-	95.45	593	-	-	*Dominikia lithuanica* strain 331-2 small subunit ribosomal RNA gene, partial sequence; internal transcribed spacer 1, 5.8S ribosomal RNA gene, and internal transcribed spacer 2, complete sequence; and large subunit ribosomal RNA gene, partial sequence

query	subject	Bs1S		Bs2S		Rs1S		Rs2S		Description
		% coverage	bp query	% coverage	bp query	% coverage	bp query	% coverage	bp query	
de novo _16181	KX758116.1	-	-	-	-	94.17	480	-	-	*Dominikia lithuanica* strain 331-2 small subunit ribosomal RNA gene, partial sequence; internal transcribed spacer 1, 5.8S ribosomal RNA gene, and internal transcribed spacer 2, complete sequence; and large subunit ribosomal RNA gene, partial sequence
de novo _16217	KX758116.1	-	-	-	-	94.51	674	-	-	*Dominikia lithuanica* strain 331-2 small subunit ribosomal RNA gene, partial sequence; internal transcribed spacer 1, 5.8S ribosomal RNA gene, and internal transcribed spacer 2, complete sequence; and large subunit ribosomal RNA gene, partial sequence
de novo _16220	KX758116.1	-	-	-	-	95.24	504	-	-	*Dominikia lithuanica* strain 331-2 small subunit ribosomal RNA gene, partial sequence; internal transcribed spacer 1, 5.8S ribosomal RNA gene, and internal transcribed spacer 2, complete sequence; and large subunit ribosomal RNA gene, partial sequence
de novo _16309	AM947928.1	-	-	-	-	96.45	677	-	-	Uncultured glomeromycete partial 25S rRNA gene, clone CSS-Ba5
de novo _164	HE858375.1	-	-	98.32	713	-	-	-	-	Uncultured *Glomus* partial 28S rRNA gene, clone WW1-6
de novo _167	HE858378.1	-	-	98.45	710	98.45	710	-	-	Uncultured *Glomus* partial 28S rRNA gene, clone WW2-5
de novo _17	HF970216.1	98.93	654	98.93	654	98.93	654	98.93	654	Uncultured *Archaeospora* genomic DNA containing 18S rRNA gene, ITS1, 5.8S rRNA gene, ITS2 and 28S rRNA gene, clone CS030-02
de novo _17324	EU380084.1	-	-	-	-	-	-	93.95	711	Uncultured glomeromycete from green needle grass 2_38 25S ribosomal RNA gene, partial sequence
de novo _17416	JX096582.1	-	-	-	-	-	-	98.44	706	Uncultured *Glomeromycota* clone 4_16 18S ribosomal RNA gene, partial sequence; internal transcribed spacer 1, 5.8S ribosomal RNA gene, and internal transcribed spacer 2, complete sequence; and 28S ribosomal RNA gene, partial sequence
de novo _17976	HE858375.1	-	-	-	-	98.46	712	-	-	Uncultured *Glomus* partial 28S rRNA gene, clone WW1-6
de novo _18	HE858375.1	98.46	714	98.46	714	98.46	714	98.46	714	Uncultured *Glomus* partial 28S rRNA gene, clone WW1-6
de novo _182	HE858375.1	-	-	97.21	718	97.21	718	97.21	718	Uncultured *Glomus* partial 28S rRNA gene, clone WW1-6
de novo _1853	HG425888.1	98.07	673	-	-	-	-	-	-	Uncultured *Rhizophagus* genomic DNA containing 18S rRNA gene, ITS1, 5.8S rRNA gene, ITS2 and 28S rRNA gene, clone JavorIX1
de novo _18730	HE794042.1	-	-	-	-	-	-	95.32	555	Uncultured *Glomeraceae* genomic DNA containing 18S rRNA gene, ITS1, 5.8S rRNA gene, ITS2 and 28S rRNA gene, clone pb-pi-4
de novo _18737	HE794042.1	-	-	-	-	-	-	96.42	475	Uncultured *Glomeraceae* genomic DNA containing 18S rRNA gene, ITS1, 5.8S rRNA gene, ITS2 and 28S rRNA gene, clone pb-pi-4
de novo _18752	HE794042.1	-	-	-	-	-	-	97.33	449	Uncultured *Glomeraceae* genomic DNA containing 18S rRNA gene, ITS1, 5.8S rRNA gene, ITS2 and 28S rRNA gene, clone pb-pi-4
de novo _18788	HE794042.1	-	-	-	-	-	-	97.19	463	Uncultured *Glomeraceae* genomic DNA containing 18S rRNA gene, ITS1, 5.8S rRNA gene, ITS2 and 28S rRNA gene, clone pb-pi-4
de novo _18845	HE794042.1	-	-	-	-	-	-	96.75	461	Uncultured *Glomeraceae* genomic DNA containing 18S rRNA gene, ITS1, 5.8S rRNA gene, ITS2 and 28S rRNA gene, clone pb-pi-4
de novo _1926	KF849683.1	93.84	714	93.84	714	-	-	93.84	714	Uncultured *Glomus* clone AM203 18S ribosomal RNA gene, partial sequence; internal transcribed spacer 1, 5.8S ribosomal RNA gene, and internal transcribed spacer 2, complete sequence; and 28S ribosomal RNA gene, partial sequence
de novo _2	HE858375.1	99.04	728	99.04	728	99.04	728	99.04	728	Uncultured *Glomus* partial 28S rRNA gene, clone WW1-6
de novo _2072	KC410882.1	96.05	708	-	-	-	-	-	-	Uncultured *Glomerales* clone H1_14 large subunit ribosomal RNA gene, partial sequence
de novo _2075	KC410882.1	95.77	709	-	-	-	-	-	-	Uncultured *Glomerales* clone H1_14 large subunit ribosomal RNA gene, partial sequence
de novo _2076	KC410882.1	95.76	708	-	-	-	-	-	-	Uncultured *Glomerales* clone H1_14 large subunit ribosomal RNA gene, partial sequence
de novo _2077	KC410882.1	96.02	729	-	-	-	-	-	-	Uncultured *Glomerales* clone H1_14 large subunit ribosomal RNA gene, partial sequence

query	subject	Bs1S		Bs2S		Rs1S		Rs2S		Description
		% coverage	bp query	% coverage	bp query	% coverage	bp query	% coverage	bp query	
de novo _2084	KC410882.1	95.98	697	-	-	-	-	-	-	Uncultured *Glomerales* clone H1_14 large subunit ribosomal RNA gene, partial sequence
de novo _2085	KC410882.1	95.9	707	-	-	-	-	-	-	Uncultured *Glomerales* clone H1_14 large subunit ribosomal RNA gene, partial sequence
de novo _2087	KC410882.1	95.75	706	-	-	-	-	-	-	Uncultured *Glomerales* clone H1_14 large subunit ribosomal RNA gene, partial sequence
de novo _2097	KC410882.1	95.61	729	-	-	-	-	-	-	Uncultured *Glomerales* clone H1_14 large subunit ribosomal RNA gene, partial sequence
de novo _21	HE858375.1	98.6	712	98.6	712	98.6	712	-	-	Uncultured *Glomus* partial 28S rRNA gene, clone WW1-6
de novo _2101	KC410882.1	96.08	714	-	-	-	-	-	-	Uncultured *Glomerales* clone H1_14 large subunit ribosomal RNA gene, partial sequence
de novo _2109	KC410882.1	95.9	708	-	-	-	-	-	-	Uncultured *Glomerales* clone H1_14 large subunit ribosomal RNA gene, partial sequence
de novo _2110	KC410882.1	95.49	710	-	-	-	-	-	-	Uncultured *Glomerales* clone H1_14 large subunit ribosomal RNA gene, partial sequence
de novo _2113	KC410882.1	95.63	710	-	-	-	-	-	-	Uncultured *Glomerales* clone H1_14 large subunit ribosomal RNA gene, partial sequence
de novo _2115	KC410882.1	96.15	727	-	-	-	-	-	-	Uncultured *Glomerales* clone H1_14 large subunit ribosomal RNA gene, partial sequence
de novo _2119	KC410882.1	95.76	708	-	-	-	-	-	-	Uncultured *Glomerales* clone H1_14 large subunit ribosomal RNA gene, partial sequence
de novo _212	HE858375.1	-	-	-	-	98.73	710	-	-	Uncultured *Glomus* partial 28S rRNA gene, clone WW1-6
de novo _2136	KC410882.1	95.48	708	-	-	-	-	-	-	Uncultured *Glomerales* clone H1_14 large subunit ribosomal RNA gene, partial sequence
de novo _2137	KC410882.1	95.76	708	-	-	-	-	-	-	Uncultured *Glomerales* clone H1_14 large subunit ribosomal RNA gene, partial sequence
de novo _2155	KC410882.1	95.9	708	-	-	-	-	-	-	Uncultured *Glomerales* clone H1_14 large subunit ribosomal RNA gene, partial sequence
de novo _2159	KC410882.1	95.98	697	-	-	-	-	-	-	Uncultured *Glomerales* clone H1_14 large subunit ribosomal RNA gene, partial sequence
de novo _2163	KC410882.1	95.49	710	-	-	-	-	-	-	Uncultured *Glomerales* clone H1_14 large subunit ribosomal RNA gene, partial sequence
de novo _2177	KC410882.1	95.91	709	-	-	-	-	-	-	Uncultured *Glomerales* clone H1_14 large subunit ribosomal RNA gene, partial sequence
de novo _2201	KC410882.1	95.23	713	-	-	-	-	-	-	Uncultured *Glomerales* clone H1_14 large subunit ribosomal RNA gene, partial sequence
de novo _2243	KC410882.1	95.64	711	-	-	-	-	-	-	Uncultured *Glomerales* clone H1_14 large subunit ribosomal RNA gene, partial sequence
de novo _231	HE858375.1	-	-	98.32	713	98.32	713	-	-	Uncultured *Glomus* partial 28S rRNA gene, clone WW1-6
de novo _2452	KC410882.1	95.55	697	-	-	-	-	-	-	Uncultured *Glomerales* clone H1_14 large subunit ribosomal RNA gene, partial sequence
de novo _2464	KC410882.1	95.34	708	-	-	-	-	-	-	Uncultured *Glomerales* clone H1_14 large subunit ribosomal RNA gene, partial sequence
de novo _25	HE858375.1	-	-	99.16	711	99.16	711	99.16	711	Uncultured *Glomus* partial 28S rRNA gene, clone WW1-6
de novo _2605	KC410882.1	95.68	695	-	-	-	-	-	-	Uncultured *Glomerales* clone H1_14 large subunit ribosomal RNA gene, partial sequence
de novo _263	HE858375.1	-	-	-	-	97.89	711	97.89	711	Uncultured *Glomus* partial 28S rRNA gene, clone WW1-6
de novo _2678	KC410882.1	95.63	709	-	-	-	-	-	-	Uncultured *Glomerales* clone H1_14 large subunit ribosomal RNA gene, partial sequence
de novo _274	HE858378.1	-	-	98.47	721	98.47	721	98.47	721	Uncultured *Glomus* partial 28S rRNA gene, clone WW2-5
de novo _2752	KC410882.1	95.88	655	-	-	-	-	-	-	Uncultured *Glomerales* clone H1_14 large subunit ribosomal RNA gene, partial sequence
de novo _2819	KC410882.1	94.26	732	-	-	-	-	-	-	Uncultured *Glomerales* clone H1_14 large subunit ribosomal RNA gene, partial sequence
de novo _3	HE858375.1	99.17	721	99.17	721	99.17	721	99.17	721	Uncultured *Glomus* partial 28S rRNA gene, clone WW1-6
de novo _30	HE858375.1	98.9	730	98.9	730	98.9	730	98.9	730	Uncultured *Glomus* partial 28S rRNA gene, clone WW1-6
de novo _314	HE858375.1	-	-	-	-	98.87	711	-	-	Uncultured *Glomus* partial 28S rRNA gene, clone WW1-6
de novo _316	HE858375.1	-	-	-	-	-	-	98.87	709	Uncultured *Glomus* partial 28S rRNA gene, clone WW1-6
de novo _32	HE858375.1	-	-	-	-	-	-	98.45	709	Uncultured *Glomus* partial 28S rRNA gene, clone WW1-6
de novo _357	HE858375.1	-	-	-	-	99.15	710	-	-	Uncultured *Glomus* partial 28S rRNA gene, clone WW1-6
de novo _3585	KC410882.1	94.16	668	-	-	-	-	-	-	Uncultured *Glomerales* clone H1_14 large subunit ribosomal RNA gene, partial sequence
de novo _36	HE858375.1	98.77	730	-	-	-	-	-	-	Uncultured *Glomus* partial 28S rRNA gene, clone WW1-6
de novo _3738	HF970216.1	98.89	633	98.89	633	-	-	-	-	Uncultured *Archaeospora* genomic DNA containing 18S rRNA gene, ITS1, 5.8S rRNA gene, ITS2 and 28S rRNA gene, clone CS030-02

query	subject	Bs1S		Bs2S		Rs1S		Rs2S		Description
		% coverage	bp query	% coverage	bp query	% coverage	bp query	% coverage	bp query	
de novo _3745	HF970216.1	-	-	91.24	651	-	-	-	-	Uncultured *Archaeospora* genomic DNA containing 18S rRNA gene, ITS1, 5.8S rRNA gene, ITS2 and 28S rRNA gene, clone CS030-02
de novo _38	FR750530.1	96.33	708	-	-	-	-	-	-	*Glomus macrocarpum* 18S rRNA gene (partial), ITS1, 5.8S rRNA gene, ITS2 and 28S rRNA gene (partial), specimen voucher W5288, clone pHS093-49
de novo _3815	HG425889.1	-	-	90.54	719	-	-	-	-	Uncultured *Rhizophagus* genomic DNA containing 18S rRNA gene, ITS1, 5.8S rRNA gene, ITS2 and 28S rRNA gene, clone Olse K-X19
de novo _3882	HF970216.1	-	-	91.38	650	-	-	-	-	Uncultured *Archaeospora* genomic DNA containing 18S rRNA gene, ITS1, 5.8S rRNA gene, ITS2 and 28S rRNA gene, clone CS030-02
de novo _39	HE858375.1	-	-	98.63	731	98.63	731	98.63	731	Uncultured *Glomus* partial 28S rRNA gene, clone WW1-6
de novo _3911	HG425889.1	-	-	90.79	706	-	-	-	-	Uncultured *Rhizophagus* genomic DNA containing 18S rRNA gene, ITS1, 5.8S rRNA gene, ITS2 and 28S rRNA gene, clone Olse K-X19
de novo _392	HE858375.1	-	-	-	-	97.69	736	-	-	Uncultured *Glomus* partial 28S rRNA gene, clone WW1-6
de novo _3952	HE775295.1	-	-	97.46	669	-	-	-	-	Uncultured *Rhizophagus* 18S rRNA gene (partial), ITS1, 5.8S rRNA gene, ITS2 and 28S rRNA gene (partial), tissue library 32e, clone library KRU, clone 17
de novo _397	HE858375.1	-	-	97.34	713	-	-	-	-	Uncultured *Glomus* partial 28S rRNA gene, clone WW1-6
de novo _4	HE858414.1	98.76	725	-	-	-	-	98.76	725	Uncultured *Glomus* partial 28S rRNA gene, clone FW4-6
de novo _414	HE858378.1	98.19	719	98.19	719	98.19	719	98.19	719	Uncultured *Glomus* partial 28S rRNA gene, clone WW2-5
de novo _4193	HF970216.1	-	-	98.26	633	-	-	-	-	Uncultured *Archaeospora* genomic DNA containing 18S rRNA gene, ITS1, 5.8S rRNA gene, ITS2 and 28S rRNA gene, clone CS030-02
de novo _4242	HF970216.1	-	-	90.95	652	-	-	-	-	Uncultured *Archaeospora* genomic DNA containing 18S rRNA gene, ITS1, 5.8S rRNA gene, ITS2 and 28S rRNA gene, clone CS030-02
de novo _4281	JQ029749.1	-	-	-	-	97.32	710	-	-	Uncultured *Glomus* clone 44 large subunit ribosomal RNA gene, partial sequence
de novo _442	HE858375.1	-	-	-	-	93.39	726	93.39	726	Uncultured *Glomus* partial 28S rRNA gene, clone WW1-6
de novo _4477	HG425888.1	98.69	685	-	-	-	-	-	-	Uncultured *Rhizophagus* genomic DNA containing 18S rRNA gene, ITS1, 5.8S rRNA gene, ITS2 and 28S rRNA gene, clone JavorIX1
de novo _4580	KC411147.1	-	-	-	-	-	-	94.92	708	Uncultured *Glomerales* clone H19_29 large subunit ribosomal RNA gene, partial sequence
de novo _4630	AJ854628.1	95.1	674	-	-	-	-	-	-	*Glomus* sp. MUCL 43207 28S rRNA gene, strain MUCL 43207, clone 6
de novo _4631	HE858375.1	-	-	-	-	99.15	708	-	-	Uncultured *Glomus* partial 28S rRNA gene, clone WW1-6
de novo _4632	AB369732.1	96.92	715	-	-	-	-	-	-	Uncultured *Glomus* gene for 28S ribosomal RNA, partial sequence, clone: R7-16
de novo _4634	JN937243.1	97.49	717	-	-	-	-	97.49	717	*Glomeromycota* sp. OTU3 DJMC-2012 isolate spore 1_9_2 28S ribosomal RNA gene, partial sequence
de novo _4637	AJ854588.1	94.16	702	-	-	-	-	-	-	*Glomus* sp. MUCL 43203 28S rRNA gene, strain MUCL 43203, clone 5
de novo _4642	AJ854588.1	97.48	713	-	-	-	-	97.48	713	*Glomus* sp. MUCL 43203 28S rRNA gene, strain MUCL 43203, clone 5
de novo _4649	AB369732.1	95.05	707	-	-	-	-	-	-	Uncultured *Glomus* gene for 28S ribosomal RNA, partial sequence, clone: R7-16
de novo _468	HE858375.1	-	-	-	-	99.01	710	-	-	Uncultured *Glomus* partial 28S rRNA gene, clone WW1-6
de novo _4727	AB369732.1	-	-	97.37	722	-	-	-	-	Uncultured *Glomus* gene for 28S ribosomal RNA, partial sequence, clone: R7-16
de novo _4732	JQ029748.1	-	-	97.35	717	-	-	-	-	Uncultured *Glomus* clone 18 large subunit ribosomal RNA gene, partial sequence
de novo _4734	AJ854612.1	-	-	96.59	733	-	-	-	-	*Glomus* sp. MUCL 43205 28S rRNA gene, strain MUCL 43205, clone 12
de novo _4736	JQ029748.1	-	-	88.84	717	-	-	-	-	Uncultured *Glomus* clone 18 large subunit ribosomal RNA gene, partial sequence
de novo _4749	JQ029748.1	-	-	93.37	709	-	-	-	-	Uncultured *Glomus* clone 18 large subunit ribosomal RNA gene, partial sequence

query	subject	Bs1S		Bs2S		Rs1S		Rs2S		Description
		% coverage	bp query	% coverage	bp query	% coverage	bp query	% coverage	bp query	
de novo _4750	AJ854624.1	-	-	89	718	-	-	-	-	*Glomus* sp. MUCL 43207 28S rRNA gene, strain MUCL 43207, clone 2
de novo _4751	AY639294.1	-	-	90.34	714	-	-	-	-	*Rhizophagus* cf. *intraradices* HG-2010 clone 28.1 28S large subunit ribosomal RNA gene, partial sequence
de novo _4757	KC410946.1	-	-	87.31	733	-	-	-	-	Uncultured *Glomerales* clone H3_24 large subunit ribosomal RNA gene, partial sequence
de novo _4767	KF849683.1	-	-	98.5	732	98.5	732	-	-	Uncultured *Glomus* clone AM203 18S ribosomal RNA gene, partial sequence
de novo _4768	KC410946.1	-	-	87.64	712	-	-	-	-	Uncultured *Glomerales* clone H3_24 large subunit ribosomal RNA gene, partial sequence
de novo _4788	HF970216.1	-	-	97.39	651	-	-	-	-	Uncultured *Archaeospora* genomic DNA containing 18S rRNA gene, ITS1, 5.8S rRNA gene, ITS2 and 28S rRNA gene, clone CS030-02
de novo _4809	KC410946.1	-	-	86.66	712	-	-	-	-	Uncultured *Glomerales* clone H3_24 large subunit ribosomal RNA gene, partial sequence
de novo _481	HE858378.1	-	-	-	-	98.6	712	-	-	Uncultured *Glomus* partial 28S rRNA gene, clone WW2-5
de novo _483	HE858375.1	98.34	723	-	-	98.34	723	-	-	Uncultured *Glomus* partial 28S rRNA gene, clone WW1-6
de novo _4870	KC410946.1	-	-	90.32	713	-	-	-	-	Uncultured *Glomerales* clone H3_24 large subunit ribosomal RNA gene, partial sequence
de novo _4876	KF849697.1	-	-	-	-	-	-	87.78	704	Uncultured *Glomus* clone AM217 18S ribosomal RNA gene, partial sequence; internal transcribed spacer 1, 5.8S ribosomal RNA gene, and internal transcribed spacer 2, complete sequence; and 28S ribosomal RNA gene, partial sequence
de novo _5	HQ128640.1	93.18	704	93.18	704	93.18	704	93.18	704	Uncultured *Glomeromycota* clone IIA1a32 25S ribosomal RNA gene, partial sequence
de novo _5161	HG425888.1	99.24	658	-	-	-	-	-	-	Uncultured *Rhizophagus* genomic DNA containing 18S rRNA gene, ITS1, 5.8S rRNA gene, ITS2 and 28S rRNA gene, clone JavorIX1
de novo _5162	HG425888.1	98.88	712	-	-	-	-	-	-	Uncultured *Rhizophagus* genomic DNA containing 18S rRNA gene, ITS1, 5.8S rRNA gene, ITS2 and 28S rRNA gene, clone JavorIX1
de novo _5165	HF970216.1	98.14	644	-	-	-	-	-	-	Uncultured *Archaeospora* genomic DNA containing 18S rRNA gene, ITS1, 5.8S rRNA gene, ITS2 and 28S rRNA gene, clone CS030-02
de novo _5166	HG425888.1	99.11	671	-	-	-	-	-	-	Uncultured *Rhizophagus* genomic DNA containing 18S rRNA gene, ITS1, 5.8S rRNA gene, ITS2 and 28S rRNA gene, clone JavorIX1
de novo _5174	HG969377.1	96.51	716	-	-	-	-	-	-	*Glomus invermaium* genomic DNA containing 18S rRNA gene, ITS1, 5.8S rRNA gene, ITS2 and 28S rRNA gene, isolate Att1646, clone ECU102P9
de novo _5180	HG425888.1	98.83	682	-	-	-	-	-	-	Uncultured *Rhizophagus* genomic DNA containing 18S rRNA gene, ITS1, 5.8S rRNA gene, ITS2 and 28S rRNA gene, clone JavorIX1
de novo _5181	HG425888.1	98.31	652	-	-	-	-	-	-	Uncultured *Rhizophagus* genomic DNA containing 18S rRNA gene, ITS1, 5.8S rRNA gene, ITS2 and 28S rRNA gene, clone JavorIX1
de novo _5185	HF970216.1	-	-	98.76	644	-	-	-	-	Uncultured *Archaeospora* genomic DNA containing 18S rRNA gene, ITS1, 5.8S rRNA gene, ITS2 and 28S rRNA gene, clone CS030-02
de novo _5187	HG425888.1	98.44	707	-	-	-	-	-	-	Uncultured *Rhizophagus* genomic DNA containing 18S rRNA gene, ITS1, 5.8S rRNA gene, ITS2 and 28S rRNA gene, clone JavorIX1
de novo _5216	HG425888.1	97.78	677	-	-	-	-	-	-	Uncultured *Rhizophagus* genomic DNA containing 18S rRNA gene, ITS1, 5.8S rRNA gene, ITS2 and 28S rRNA gene, clone JavorIX1
de novo _5225	JF439109.1	-	-	96.85	699	-	-	-	-	*Glomus intraradices* isolate B22-11 18S ribosomal RNA gene, partial sequence
de novo _5282	HF970216.1	97.31	632	-	-	-	-	-	-	Uncultured *Archaeospora* genomic DNA containing 18S rRNA gene, ITS1, 5.8S rRNA gene, ITS2 and 28S rRNA gene, clone CS030-02

query	subject	Bs1S		Bs2S		Rs1S		Rs2S		Description
		% coverage	bp query	% coverage	bp query	% coverage	bp query	% coverage	bp query	
de novo _5382	HG425888.1	98.15	704	-	-	-	-	-	-	Uncultured *Rhizophagus* genomic DNA containing 18S rRNA gene, ITS1, 5.8S rRNA gene, ITS2 and 28S rRNA gene, clone JavorIX1
de novo _547	HE858375.1	-	-	-	-	98.61	718	98.61	718	Uncultured *Glomus* partial 28S rRNA gene, clone WW1-6
de novo _58	HE858375.1	98.59	710	-	-	-	-	-	-	Uncultured *Glomus* partial 28S rRNA gene, clone WW1-6
de novo _580	HE858375.1	-	-	98.77	730	98.77	730	98.77	730	Uncultured *Glomus* partial 28S rRNA gene, clone WW1-6
de novo _6072	HG425888.1	98.83	681	-	-	-	-	-	-	Uncultured *Rhizophagus* genomic DNA containing 18S rRNA gene, ITS1, 5.8S rRNA gene, ITS2 and 28S rRNA gene, clone JavorIX1
de novo _609	HG425888.1	98.35	726	98.35	726	-	-	-	-	Uncultured *Rhizophagus* genomic DNA containing 18S rRNA gene, ITS1, 5.8S rRNA gene, ITS2 and 28S rRNA gene, clone JavorIX1
de novo _610	HG425888.1	99.17	720	99.17	720	-	-	-	-	Uncultured *Rhizophagus* genomic DNA containing 18S rRNA gene, ITS1, 5.8S rRNA gene, ITS2 and 28S rRNA gene, clone JavorIX1
de novo _611	HE775295.1	98.6	714	98.6	714	-	-	-	-	Uncultured *Rhizophagus* 18S rRNA gene (partial), ITS1, 5.8S rRNA gene, ITS2 and 28S rRNA gene (partial), tissue library 32e, clone library KRU, clone 17
de novo _6124	HE858395.1	-	-	97.89	710	97.89	710	-	-	Uncultured *Glomus* partial 28S rRNA gene, clone FW4-2
de novo _6125	HE858375.1	-	-	99.16	711	99.16	711	-	-	Uncultured *Glomus* partial 28S rRNA gene, clone WW1-6
de novo _6128	HE858378.1	-	-	97.76	713	97.76	713	-	-	Uncultured *Glomus* partial 28S rRNA gene, clone WW2-5
de novo _6131	HE858375.1	-	-	98.77	732	-	-	-	-	Uncultured *Glomus* partial 28S rRNA gene, clone WW1-6
de novo _6133	HE858375.1	-	-	98.89	720	98.89	720	-	-	Uncultured *Glomus* partial 28S rRNA gene, clone WW1-6
de novo _6134	HE858375.1	-	-	99.01	709	99.01	709	-	-	Uncultured *Glomus* partial 28S rRNA gene, clone WW1-6
de novo _6135	HE858375.1	-	-	98.46	712	-	-	-	-	Uncultured *Glomus* partial 28S rRNA gene, clone WW1-6
de novo _614	HG425888.1	99.17	721	99.17	721	-	-	-	-	Uncultured *Rhizophagus* genomic DNA containing 18S rRNA gene, ITS1, 5.8S rRNA gene, ITS2 and 28S rRNA gene, clone JavorIX1
de novo _6146	HM215993.1	-	-	95.02	562	-	-	-	-	Uncultured *Glomus* clone GLO_AL36-SSN45 28S ribosomal RNA gene, partial sequence
de novo _615	HG425888.1	98.76	725	98.76	725	-	-	-	-	Uncultured *Rhizophagus* genomic DNA containing 18S rRNA gene, ITS1, 5.8S rRNA gene, ITS2 and 28S rRNA gene, clone JavorIX1
de novo _6151	HE858375.1	-	-	99.16	712	-	-	-	-	Uncultured *Glomus* partial 28S rRNA gene, clone WW1-6
de novo _6155	HE858375.1	-	-	98.74	712	98.74	712	-	-	Uncultured *Glomus* partial 28S rRNA gene, clone WW1-6
de novo _6161	HE858375.1	-	-	98.88	712	-	-	-	-	Uncultured *Glomus* partial 28S rRNA gene, clone WW1-6
de novo _6171	HE858375.1	-	-	98.6	712	-	-	-	-	Uncultured *Glomus* partial 28S rRNA gene, clone WW1-6
de novo _6172	HE858375.1	-	-	98.46	714	-	-	98.46	714	Uncultured *Glomus* partial 28S rRNA gene, clone WW1-6
de novo _6175	HE858375.1	-	-	97.99	698	97.99	698	-	-	Uncultured *Glomus* partial 28S rRNA gene, clone WW1-6
de novo _6176	HF970216.1	-	-	98.45	645	-	-	-	-	Uncultured *Archaeospora* genomic DNA containing 18S rRNA gene, ITS1, 5.8S rRNA gene, ITS2 and 28S rRNA gene, clone CS030-02
de novo _6185	HM215993.1	-	-	95.64	574	-	-	-	-	Uncultured *Glomus* clone GLO_AL36-SSN45 28S ribosomal RNA gene, partial sequence
de novo _619	FR871375.1	-	-	88.82	680	-	-	-	-	Uncultured *Glomus* partial 28S rRNA gene, clone BROS2-31
de novo _6209	HE858395.1	-	-	96.86	732	-	-	-	-	Uncultured *Glomus* partial 28S rRNA gene, clone FW4-2
de novo _6229	HE858395.1	-	-	97.19	712	-	-	-	-	Uncultured *Glomus* partial 28S rRNA gene, clone FW4-2
de novo _6233	HE858378.1	-	-	98.03	710	98.03	710	-	-	Uncultured *Glomus* partial 28S rRNA gene, clone WW2-5
de novo _624	HG425888.1	98.76	725	98.76	725	-	-	-	-	Uncultured *Rhizophagus* genomic DNA containing 18S rRNA gene, ITS1, 5.8S rRNA gene, ITS2 and 28S rRNA gene, clone JavorIX1

query	subject	Bs1S		Bs2S		Rs1S		Rs2S		Description
		% coverage	bp query	% coverage	bp query	% coverage	bp query	% coverage	bp query	
de novo _627	HG425888.1	98.21	725	98.21	725	-	-	-	-	Uncultured *Rhizophagus* genomic DNA containing 18S rRNA gene, ITS1, 5.8S rRNA gene, ITS2 and 28S rRNA gene, clone JavorIX1
de novo _6276	HE858378.1	-	-	96.67	720	-	-	-	-	Uncultured *Glomus* partial 28S rRNA gene, clone WW2-5
de novo _632	HG425888.1	97.75	711	97.75	711	-	-	-	-	Uncultured *Rhizophagus* genomic DNA containing 18S rRNA gene, ITS1, 5.8S rRNA gene, ITS2 and 28S rRNA gene, clone JavorIX1
de novo _633	JQ029748.1	-	-	98.59	707	98.59	707	98.59	707	Uncultured *Glomus* clone 18 large subunit ribosomal RNA gene, partial sequence
de novo _637	JF439160.1	-	-	96.47	708	96.47	708	-	-	*Glomus intraradices* isolate 08_37_1 18S ribosomal RNA gene, partial sequence
de novo _6384	HE858375.1	-	-	97.78	720	-	-	-	-	Uncultured *Glomus* partial 28S rRNA gene, clone WW1-6
de novo _6400	HE858375.1	-	-	-	-	98.87	711	-	-	Uncultured *Glomus* partial 28S rRNA gene, clone WW1-6
de novo _6413	HE858375.1	-	-	-	-	97.94	727	-	-	Uncultured *Glomus* partial 28S rRNA gene, clone WW1-6
de novo _642	HG425888.1	98.16	706	98.16	706	-	-	-	-	Uncultured *Rhizophagus* genomic DNA containing 18S rRNA gene, ITS1, 5.8S rRNA gene, ITS2 and 28S rRNA gene, clone JavorIX1
de novo _645	HG425888.1	98.84	688	98.84	688	-	-	-	-	Uncultured *Rhizophagus* genomic DNA containing 18S rRNA gene, ITS1, 5.8S rRNA gene, ITS2 and 28S rRNA gene, clone JavorIX1
de novo _6566	HE858378.1	-	-	98.17	709	98.17	709	-	-	Uncultured *Glomus* partial 28S rRNA gene, clone WW2-5
de novo _657	HG425888.1	98.1	685	98.1	685	-	-	-	-	Uncultured *Rhizophagus* genomic DNA containing 18S rRNA gene, ITS1, 5.8S rRNA gene, ITS2 and 28S rRNA gene, clone JavorIX1
de novo _6577	HE858375.1	-	-	97.91	716	97.91	716	97.91	716	Uncultured *Glomus* partial 28S rRNA gene, clone WW1-6
de novo _6598	AY639360.1	-	-	99.53	645	-	-	-	-	Uncultured *Glomeromycota* clone 6.11 28S large subunit ribosomal RNA gene, partial sequence
de novo _667	HG425888.1	98.34	723	98.34	723	-	-	-	-	Uncultured *Rhizophagus* genomic DNA containing 18S rRNA gene, ITS1, 5.8S rRNA gene, ITS2 and 28S rRNA gene, clone JavorIX1
de novo _6735	HE858375.1	-	-	98.9	726	98.9	726	98.9	726	Uncultured *Glomus* partial 28S rRNA gene, clone WW1-6
de novo _68	HE858375.1	-	-	-	-	98.77	732	98.77	732	Uncultured *Glomus* partial 28S rRNA gene, clone WW1-6
de novo _6808	HE858378.1	-	-	-	-	98.17	709	-	-	Uncultured *Glomus* partial 28S rRNA gene, clone WW2-5
de novo _691	HG425889.1	97.6	709	97.6	709	-	-	-	-	Uncultured *Rhizophagus* genomic DNA containing 18S rRNA gene, ITS1, 5.8S rRNA gene, ITS2 and 28S rRNA gene, clone Olse K-X19
de novo _700	HG425888.1	98.89	722	-	-	-	-	-	-	Uncultured *Rhizophagus* genomic DNA containing 18S rRNA gene, ITS1, 5.8S rRNA gene, ITS2 and 28S rRNA gene, clone JavorIX1
de novo _735	JX096582.1	-	-	95.31	704	-	-	95.31	704	Uncultured *Glomeromycota* clone 4_16 18S ribosomal RNA gene, partial sequence
de novo _736	HE775323.1	-	-	-	-	98.3	707	98.3	707	Uncultured *Rhizophagus* 18S rRNA gene (partial), ITS1, 5.8S rRNA gene, ITS2 and 28S rRNA gene (partial), tissue library 3/1-4/1-6/1, clone library KRU, clone 7
de novo _754	HG425888.1	-	-	98.42	696	-	-	-	-	Uncultured *Rhizophagus* genomic DNA containing 18S rRNA gene, ITS1, 5.8S rRNA gene, ITS2 and 28S rRNA gene, clone JavorIX1
de novo _7540	HE858395.1	-	-	-	-	96.67	720	-	-	Uncultured *Glomus* partial 28S rRNA gene, clone FW4-2
de novo _7630	HE858378.1	-	-	-	-	98.17	710	-	-	Uncultured *Glomus* partial 28S rRNA gene, clone WW2-5
de novo _770	HG425888.1	98.74	714	-	-	-	-	-	-	Uncultured *Rhizophagus* genomic DNA containing 18S rRNA gene, ITS1, 5.8S rRNA gene, ITS2 and 28S rRNA gene, clone JavorIX1
de novo _772	FR871363.1	-	-	93.42	714	-	-	-	-	Uncultured *Glomus* partial 28S rRNA gene, clone BROS3-12
de novo _7787	HE858375.1	-	-	-	-	98.62	725	98.62	725	Uncultured *Glomus* partial 28S rRNA gene, clone WW1-6
de novo _780	HE858395.1	-	-	97.21	718	97.21	718	97.21	718	Uncultured *Glomus* partial 28S rRNA gene, clone FW4-2

query	subject	Bs1S		Bs2S		Rs1S		Rs2S		Description
		% coverage	bp query	% coverage	bp query	% coverage	bp query	% coverage	bp query	
de novo _797	HG425888.1	99.27	684	-	-	-	-	-	-	Uncultured *Rhizophagus* genomic DNA containing 18S rRNA gene, ITS1, 5.8S rRNA gene, ITS2 and 28S rRNA gene, clone JavorIX1
de novo _8	HE858375.1	98.9	730	98.9	730	98.9	730	98.9	730	Uncultured *Glomus* partial 28S rRNA gene, clone WW1-6
de novo _811	HG425888.1	-	-	90.47	724	90.47	724	90.47	724	Uncultured *Rhizophagus* genomic DNA containing 18S rRNA gene, ITS1, 5.8S rRNA gene, ITS2 and 28S rRNA gene, clone JavorIX1
de novo _8159	HE858378.1	98.6	713	98.6	713	98.6	713	98.6	713	Uncultured *Glomus* partial 28S rRNA gene, clone WW2-5
de novo _818	HF970216.1	98.76	645	98.76	645	-	-	98.76	645	Uncultured *Archaeospora* genomic DNA containing 18S rRNA gene, ITS1, 5.8S rRNA gene, ITS2 and 28S rRNA gene, clone CS030-02
de novo _8180	HE858375.1	98.59	711	-	-	-	-	98.59	711	Uncultured *Glomus* partial 28S rRNA gene, clone WW1-6
de novo _8183	HG425888.1	97.55	694	-	-	-	-	-	-	Uncultured *Rhizophagus* genomic DNA containing 18S rRNA gene, ITS1, 5.8S rRNA gene, ITS2 and 28S rRNA gene, clone JavorIX1
de novo _8186	HE858378.1	98.17	712	-	-	-	-	98.17	712	Uncultured *Glomus* partial 28S rRNA gene, clone WW2-5
de novo _8218	HE858378.1	-	-	-	-	-	-	97.83	737	Uncultured *Glomus* partial 28S rRNA gene, clone WW2-5
de novo _8299	HE858375.1	-	-	-	-	-	-	98.48	724	Uncultured *Glomus* partial 28S rRNA gene, clone WW1-6
de novo _8338	HG425889.1	97.48	675	-	-	-	-	-	-	Uncultured *Rhizophagus* genomic DNA containing 18S rRNA gene, ITS1, 5.8S rRNA gene, ITS2 and 28S rRNA gene, clone Olse K-X19
de novo _837	AB369732.1	-	-	88.99	690	-	-	-	-	Uncultured *Glomus* gene for 28S ribosomal RNA, partial sequence, clone: R7-16
de novo _840	HG425888.1	98.87	706	98.87	706	-	-	-	-	Uncultured *Rhizophagus* genomic DNA containing 18S rRNA gene, ITS1, 5.8S rRNA gene, ITS2 and 28S rRNA gene, clone JavorIX1
de novo _8438	HE858375.1	98.87	711	-	-	-	-	98.87	711	Uncultured *Glomus* partial 28S rRNA gene, clone WW1-6
de novo _8447	HE858375.1	98.45	711	-	-	-	-	98.45	711	Uncultured *Glomus* partial 28S rRNA gene, clone WW1-6
de novo _851	HE775295.1	-	-	98.18	716	-	-	-	-	Uncultured *Rhizophagus* 18S rRNA gene (partial), ITS1, 5.8S rRNA gene, ITS2 and 28S rRNA gene (partial), tissue library 32e, clone library KRU, clone 17
de novo _8557	KC411208.1	93.65	709	-	-	-	-	-	-	Uncultured *Glomerales* clone B04_41 large subunit ribosomal RNA gene, partial sequence
de novo _859	JF439109.1	96.24	719	96.24	719	-	-	-	-	*Glomus intraradices* isolate B22-11 18S ribosomal RNA gene, partial sequence; internal transcribed spacer 1, 5.8S ribosomal RNA gene, and internal transcribed spacer 2, complete sequence; and 28S ribosomal RNA gene, partial sequence
de novo _868	AJ854612.1	-	-	90.1	727	-	-	90.1	727	*Glomus* sp. MUCL 43205 28S rRNA gene, strain MUCL 43205, clone 12
de novo _8699	HQ242981.1	-	-	-	-	95.28	720	95.28	720	Uncultured *Glomus* clone ZHwq2-189 18S ribosomal RNA gene, partial sequence; internal transcribed spacer 1, 5.8S ribosomal RNA gene, and internal transcribed spacer 2, complete sequence; and 28S ribosomal RNA gene, partial sequence
de novo _8957	HE858375.1	-	-	98.18	715	-	-	-	-	Uncultured *Glomus* partial 28S rRNA gene, clone WW1-6
de novo _90	HE858375.1	98.77	731	98.77	731	98.77	731	98.77	731	Uncultured *Glomus* partial 28S rRNA gene, clone WW1-6
de novo _911	HG425888.1	99.27	685	-	-	-	-	-	-	Uncultured *Rhizophagus* genomic DNA containing 18S rRNA gene, ITS1, 5.8S rRNA gene, ITS2 and 28S rRNA gene, clone JavorIX1
de novo _913	HG425888.1	98.98	686	-	-	-	-	-	-	Uncultured *Rhizophagus* genomic DNA containing 18S rRNA gene, ITS1, 5.8S rRNA gene, ITS2 and 28S rRNA gene, clone JavorIX1
de novo _9391	HF970229.1	-	-	97.37	722	-	-	-	-	Uncultured *Funneliformis* genomic DNA containing 18S rRNA gene, ITS1, 5.8S rRNA gene, ITS2 and 28S rRNA gene, clone CS035-02
de novo _970	HG425888.1	-	-	98.17	657	-	-	-	-	Uncultured *Rhizophagus* genomic DNA containing 18S rRNA gene, ITS1, 5.8S rRNA gene, ITS2 and 28S rRNA gene, clone JavorIX1

query	subject	Bs1S		Bs2S		Rs1S		Rs2S		Description
		% coverage	bp query	% coverage	bp query	% coverage	bp query	% coverage	bp query	
de novo _98	HE858375.1	-	-	98.37	735	98.37	735	98.37	735	Uncultured *Glomus* partial 28S rRNA gene, clone WW1-6
de novo _9939	HE858375.1	-	-	-	-	96.59	704	-	-	Uncultured *Glomus* partial 28S rRNA gene, clone WW1-6
de novo _9942	AB670115.1	-	-	-	-	91.67	720	91.67	720	Uncultured *Glomus* gene for 28S ribosomal RNA, partial sequence, clone: IR36-9
de novo _9945	FJ461853.1	-	-	-	-	97.87	705	-	-	*Glomus viscosum* isolate BEG50 25S ribosomal RNA gene, partial sequence
de novo _9950	FR871359.1	-	-	-	-	96.46	734	-	-	Uncultured *Glomus* partial 28S rRNA gene, clone BRAS4-24
de novo _9959	KC411151.1	-	-	-	-	91.56	723	91.56	723	Uncultured *Glomerales* clone H19_35 large subunit ribosomal RNA gene, partial sequence

Table S3. Heat tree difference table reporting the results (p-values) of the non-parametric Wilcoxon Rank Sum test used to depict taxonomic dfferences between AMF communities. Significant taxa are highlighted in yellow.

"Bs1S" vs. "Bs2S"				
tax_name	log2_median_ratio	median_diff	mean_diff	wilcox_p_value
denovo_1185	-Inf	-0.000440012	-0.001117223	0.007494958
denovo_811	-4.32293906	-0.008389772	-0.011958704	0.011925234
Septoglomus_viscosum	-Inf	-0.00015015	-0.000272369	0.02536986
denovo_3745	-Inf	-0.002595024	-0.002700768	0.02536986
denovo_837	-Inf	-0.00015015	-0.000908849	0.02536986
denovo_3882	-Inf	-0.0003003	-0.000746955	0.072005658
denovo_4193	-Inf	-0.000146671	-0.000509394	0.072005658
denovo_4242	-Inf	-0.00164646	-0.001144362	0.072005658
denovo_4732	-Inf	-0.000299356	-0.034160536	0.072005658
denovo_619	-Inf	-0.000146671	-0.000418592	0.072005658
denovo_633	-Inf	-0.000600601	-0.023833961	0.072005658
denovo_98	-Inf	-0.0003003	-0.007631972	0.072005658
denovo_17	-4.212409263	-0.082027193	-0.039954764	0.095238095
denovo_10067	0	0	-0.000121619	0.179712495
denovo_1134	0	0	-0.000714699	0.179712495
denovo_2075	0	0	0.072011213	0.179712495
denovo_2077	0	0	0.018544766	0.179712495
denovo_2084	0	0	0.02226075	0.179712495
denovo_2113	0	0	0.004215465	0.179712495
denovo_2137	0	0	0.003578894	0.179712495
denovo_2163	0	0	0.002445218	0.179712495
denovo_2452	0	0	0.004758906	0.179712495
denovo_2678	0	0	0.00077899	0.179712495
denovo_3585	0	0	0.001160145	0.179712495
denovo_4281	0	0	-0.000238978	0.179712495
denovo_4580	0	0	-8.97E-05	0.179712495
denovo_4632	0	0	0.000501591	0.179712495
denovo_4750	0	0	-0.002841276	0.179712495
denovo_4767	0	0	-0.0006021	0.179712495
denovo_4788	0	0	-0.000851861	0.179712495
denovo_4870	0	0	-0.001566752	0.179712495
denovo_5162	0	0	0.00592363	0.179712495
denovo_5180	0	0	0.001343553	0.179712495
denovo_5181	0	0	0.0030481	0.179712495
denovo_5187	0	0	0.000671592	0.179712495
denovo_5216	0	0	0.000762855	0.179712495
denovo_5382	0	0	0.000698081	0.179712495
denovo_6072	0	0	0.000541297	0.179712495
denovo_6146	0	0	-0.000960017	0.179712495
denovo_6185	0	0	-0.001229721	0.179712495
denovo_637	0	0	-0.006308639	0.179712495
denovo_772	0	0	-0.002141185	0.179712495
denovo_8438	0	0	0.000691108	0.179712495
denovo_911	0	0	0.000487224	0.179712495
denovo_970	0	0	-0.002651516	0.179712495

denovo_780	-Inf	-0.000750751	-0.02119456	0.239316541
denovo_868	-Inf	-0.000440012	-0.005007683	0.239316541
Glomus_cf	-Inf	-0.000149098	-0.004818121	0.346521712
denovo_90	-Inf	-0.000146671	-0.001597228	0.346521712
Sclerocystis_sinuosa_MD12(	0	0	-8.98E-05	0.423710797
denovo_15	0	0	0.002465931	0.423710797
denovo_2115	0	0	0.002105879	0.423710797
denovo_212	0	0	-0.00012012	0.423710797
denovo_25	0	0	-0.00045045	0.423710797
denovo_32	0	0	0.000162232	0.423710797
denovo_4	0	0	0.058208955	0.423710797
denovo_4631	0	0	2.94E-05	0.423710797
denovo_4734	0	0	-0.000915891	0.423710797
denovo_481	0	0	-3.00E-05	0.423710797
denovo_4876	0	0	-9.16E-05	0.423710797
denovo_5174	0	0	0.000946854	0.423710797
denovo_5282	0	0	0.000458155	0.423710797
denovo_6124	0	0	-0.036426426	0.423710797
denovo_6125	0	0	-0.004744745	0.423710797
denovo_6128	0	0	-0.000780781	0.423710797
denovo_6131	0	0	-0.00039039	0.423710797
denovo_6133	0	0	-0.000840841	0.423710797
denovo_6134	0	0	-0.006156156	0.423710797
denovo_6135	0	0	-0.000780781	0.423710797
denovo_6151	0	0	-0.001291291	0.423710797
denovo_6155	0	0	-0.00042042	0.423710797
denovo_6171	0	0	-0.00042042	0.423710797
denovo_6172	0	0	-0.001411411	0.423710797
denovo_6175	0	0	-0.000630631	0.423710797
denovo_6233	0	0	-0.001141141	0.423710797
denovo_6276	0	0	-0.001111111	0.423710797
denovo_6384	0	0	-0.00039039	0.423710797
denovo_6400	0	0	-3.00E-05	0.423710797
denovo_6413	0	0	-9.01E-05	0.423710797
denovo_6566	0	0	-0.00042042	0.423710797
denovo_68	0	0	-3.00E-05	0.423710797
denovo_6808	0	0	-6.01E-05	0.423710797
denovo_736	0	0	-5.87E-05	0.423710797
denovo_7540	0	0	-3.00E-05	0.423710797
denovo_7630	0	0	-6.01E-05	0.423710797
denovo_8180	0	0	0.000452625	0.423710797
denovo_8186	0	0	0.000331925	0.423710797
denovo_8218	0	0	6.04E-05	0.423710797
denovo_8299	0	0	0.000211225	0.423710797
denovo_8447	0	0	0.000573325	0.423710797
denovo_8699	0	0	0.000271575	0.423710797
denovo_9391	0	0	-0.002394851	0.423710797
Glomus_aggregatum	0	0	-6.12E-05	0.440686016
denovo_147	0	0	0.000257779	0.440686016
denovo_2097	0	0	0.010473041	0.440686016
denovo_274	0	0	-0.00160937	0.440686016

denovo_6735	0	0	-0.000502506	0.440686016
denovo_735	0	0	-0.000836781	0.440686016
Glomus_intraradices_691_BI	-Inf	-0.000152648	-2.87E-05	0.48017689
denovo_12	-Inf	-0.003813435	0.000612426	0.48017689
denovo_4634	Inf	0.000152718	0.005740368	0.503812429
denovo_610	-Inf	-0.001068539	-0.035256667	0.590013887
denovo_2605	0	0	0.000753102	0.607235544
denovo_2819	0	0	0.005400054	0.607235544
denovo_30	0	0	-0.005036231	0.607235544
denovo_3738	0	0	-0.002926215	0.607235544
denovo_414	0	0	0.001589864	0.607235544
denovo_4642	0	0	0.00119791	0.607235544
denovo_6598	0	0	-0.000359882	0.607235544
denovo_691	0	0	-0.003573026	0.607235544
denovo_770	0	0	0.003322143	0.607235544
denovo_859	0	0	-0.000692996	0.607235544
denovo_913	0	0	0.000820036	0.607235544
Rhizophagus_intraradices	-Inf	-0.001951952	-0.037889942	0.655836497
denovo_8	-Inf	-0.0003003	0.008058389	0.655836497
Rhizophagus_sp	4.828631597	0.01674004	0.040115261	0.666430034
denovo_609	0	0	-0.114110445	0.724081661
denovo_611	0	0	-0.043846443	0.724081661
denovo_632	0	0	-0.007263372	0.724081661
Glomus_invermaium	0	0	0.012473509	0.797169693
denovo_3	0	0	-0.018621778	0.797169693
denovo_645	0	0	-0.00083208	0.797169693
denovo_667	0	0	0.005602776	0.797169693
Rhizophagus_diaphanus	Inf	0.000441241	0.000186035	0.823664389
denovo_1926	1.609430356	0.00061601	-0.036138785	0.829356961
denovo_5	-6.975532145	-0.038133002	0.039448055	0.832518813
Rhizophagus_cf	0.470712395	0.000977858	0.156729339	0.841269841
Glomus_sp	1.146139983	0.008353186	-0.029471472	0.841269841
Funneliformis_geosporum	0	0	-0.004178665	0.906329391
denovo_614	0	0	-0.011157981	0.906329391
denovo_627	0	0	0.015611433	0.906329391
denovo_818	0	0	-0.002423692	0.906329391
denovo_840	0	0	0.003755704	0.906329391
Rhizophagus_irregularis	-Inf	-0.000299356	0.002675349	0.91418355
denovo_1	0	0	0.017258454	1
denovo_1071	0	0	-0.003025604	1
denovo_1092	0	0	0.000604341	1
denovo_1131	0	0	0.00043749	1
denovo_1446	0	0	0.00033719	1
denovo_1506	0	0	0.000277262	1
denovo_151	0	0	0.000261988	1
denovo_16053	0	0	0.000146156	1
denovo_164	0	0	-0.000923682	1
denovo_167	0	0	-0.000653411	1
denovo_18	0	0	-0.000607499	1
denovo_182	0	0	-0.000974076	1
denovo_2	0	0	-0.003612887	1

denovo_21	0	0	-0.000992024	1
denovo_231	0	0	-0.000969243	1
denovo_36	0	0	0.001759641	1
denovo_39	0	0	-0.003416174	1
denovo_392	0	0	-2.24E-05	1
denovo_3952	0	0	-0.000416763	1
denovo_397	0	0	-0.000231953	1
denovo_442	0	0	-2.28E-05	1
denovo_4477	0	0	0.000426004	1
denovo_4727	0	0	-0.000613935	1
denovo_483	0	0	0.000266821	1
denovo_5165	0	0	0.004307686	1
denovo_5185	0	0	-0.000329817	1
denovo_58	0	0	0.000433886	1
denovo_580	0	0	-0.000887571	1
denovo_615	-Inf	-0.001938273	0.031556488	1
denovo_6176	0	0	-0.003843264	1
denovo_6209	0	0	-0.000570426	1
denovo_624	0	0	0.006794339	1
denovo_642	0	0	-0.002331496	1
denovo_657	0	0	0.001357227	1
denovo_6577	0	0	-0.000810376	1
denovo_700	-Inf	-0.000586682	0.004527249	1
denovo_754	0	0	-0.00062479	1
denovo_7787	0	0	6.73E-05	1
denovo_797	0	0	0.000966441	1
denovo_8159	0	0	0.02629559	1
denovo_851	0	0	-0.000416053	1
denovo_8957	0	0	-0.000388445	1

"Rs1S" vs. "Rs2S"

tax_name	log2_median_ratio	median_diff	mean_diff	wilcox_p_value
denovo_17	-Inf	-0.00955414	-0.019182399	0.034453637
denovo_11021	-Inf	-0.000182149	-0.002144679	0.072005658
denovo_11042	-Inf	-0.000182149	-0.000683664	0.072005658
denovo_4281	Inf	0.00014828	0.013562249	0.072005658
denovo_4767	Inf	0.000462963	0.012147217	0.072005658
denovo_818	-Inf	-0.000154512	-0.000879256	0.072005658
denovo_21	Inf	0.0014828	0.002662012	0.074639183
denovo_811	-Inf	-0.005762815	-0.007256276	0.138791738
denovo_8438	-Inf	-0.000312305	-0.00074193	0.157939311
denovo_39	Inf	0.0056	0.003174431	0.161237592
Rhizophagus_intraradices	0	0	-0.00010319	0.179712495
Septoglomus_jasnowskae	0	0	0.006627559	0.179712495
denovo_10048	0	0	0.0007734	0.179712495
denovo_10217	0	0	0.000614216	0.179712495
denovo_11040	0	0	-0.001278481	0.179712495
denovo_14551	0	0	-0.011876881	0.179712495
denovo_2163	0	0	-0.000186603	0.179712495
denovo_316	0	0	-0.00052968	0.179712495
denovo_3738	0	0	-0.000202981	0.179712495

denovo_4193	0	0	-0.000285911	0.179712495
denovo_4634	0	0	-0.002529341	0.179712495
denovo_4750	0	0	-0.000273547	0.179712495
denovo_5185	0	0	-0.000243954	0.179712495
denovo_6133	0	0	0.000509331	0.179712495
denovo_6146	0	0	-0.000342189	0.179712495
denovo_6185	0	0	-0.000378619	0.179712495
denovo_6209	0	0	0.000371989	0.179712495
denovo_637	0	0	0.016381656	0.179712495
denovo_735	0	0	-0.001945103	0.179712495
denovo_772	0	0	-0.000243954	0.179712495
denovo_8186	0	0	-0.001096161	0.179712495
denovo_837	0	0	-0.000434896	0.179712495
denovo_12	Inf	0.018109877	0.013281898	0.181220536
denovo_30	Inf	0.00044484	0.00605203	0.181220536
denovo_90	Inf	0.001521838	0.002844034	0.23586131
denovo_231	Inf	0.00029656	0.001074505	0.239316541
denovo_8447	-Inf	-0.000308404	-0.001506032	0.239316541
denovo_11056	-Inf	-0.001233616	-0.001593538	0.265205393
denovo_442	Inf	0.00064	-0.000408372	0.265205393
denovo_10505	-3.964882882	-0.009353754	-0.005081995	0.290346834
Rhizophagus_cf	-1.974839926	-0.00407062	-0.026102117	0.30952381
Septoglomus_viscosum	-3.148061011	-0.092127233	-0.134398963	0.30952381
Glomus_aggregatum	-Inf	-0.009525297	-0.003804643	0.332111978
denovo_25	Inf	8.00E-04	0.000829029	0.346521712
denovo_68	Inf	0.00112	0.000495185	0.346521712
denovo_98	Inf	0.00029656	0.000412493	0.346521712
denovo_1926	-Inf	-0.000154512	-0.001301267	0.37274807
Glomus_cf	0	0	-0.00065584	0.423710797
Glomus_invermaium	0	0	-0.048357749	0.423710797
denovo_16053	0	0	0.000833333	0.423710797
denovo_167	0	0	0.000416	0.423710797
denovo_2115	0	0	-3.08E-05	0.423710797
denovo_2452	0	0	-6.18E-05	0.423710797
denovo_2678	0	0	-3.08E-05	0.423710797
denovo_2819	0	0	-3.08E-05	0.423710797
denovo_3745	0	0	-0.000327869	0.423710797
denovo_3882	0	0	-3.64E-05	0.423710797
denovo_397	0	0	3.07E-05	0.423710797
denovo_4	0	0	-0.017409766	0.423710797
denovo_4242	0	0	-7.29E-05	0.423710797
denovo_4732	0	0	9.26E-05	0.423710797
denovo_4734	0	0	-0.000121322	0.423710797
denovo_4788	0	0	-3.03E-05	0.423710797
denovo_5165	0	0	3.20E-05	0.423710797
denovo_5174	0	0	-0.000123362	0.423710797
denovo_6124	0	0	0.008371665	0.423710797
denovo_6125	0	0	0.002272	0.423710797
denovo_6128	0	0	0.003434529	0.423710797
denovo_6131	0	0	9.60E-05	0.423710797
denovo_6135	0	0	3.20E-05	0.423710797

denovo_614	0	0	-0.000154512	0.423710797
denovo_6151	0	0	0.000256	0.423710797
denovo_6155	0	0	0.000352	0.423710797
denovo_6171	0	0	3.20E-05	0.423710797
denovo_6175	0	0	0.000521312	0.423710797
denovo_6176	0	0	-0.000145719	0.423710797
denovo_6233	0	0	0.001931923	0.423710797
denovo_6276	0	0	0.000275989	0.423710797
denovo_6384	0	0	3.20E-05	0.423710797
denovo_6400	0	0	0.000576	0.423710797
denovo_6566	0	0	0.001165287	0.423710797
denovo_6598	0	0	-3.08E-05	0.423710797
denovo_6808	0	0	0.00033732	0.423710797
denovo_7540	0	0	0.000459982	0.423710797
denovo_7630	0	0	0.003403864	0.423710797
denovo_770	0	0	0.000128	0.423710797
denovo_8180	0	0	-0.000648949	0.423710797
denovo_9391	0	0	4.63E-05	0.423710797
Rhizophagus_sp	0	0	-0.001244654	0.440686016
Glomus_intraradices_x99640	0	0	-0.017034811	0.440686016
denovo_10455	0	0	0.001272824	0.440686016
denovo_10699	0	0	0.001199719	0.440686016
denovo_12430	0	0	0.000445674	0.440686016
denovo_6134	0	0	0.001498044	0.440686016
denovo_8218	0	0	-0.000889111	0.440686016
denovo_9950	0	0	0.043022338	0.440686016
denovo_18	Inf	0.00208	0.000790277	0.450643728
denovo_6172	Inf	0.000153327	-6.55E-05	0.48017689
denovo_8	Inf	0.00029656	-0.01911664	0.48017689
denovo_8699	Inf	0.00014828	-0.034173113	0.48017689
denovo_6577	Inf	0.0007414	0.000400832	0.503812429
denovo_1	5.415938902	0.019531543	0.026333991	0.525808953
denovo_3	0.085521043	0.0002731	0.003528624	0.525808953
Glomus_sp	-1.2697744	-0.118769838	-0.033080466	0.547619048
denovo_8159	-Inf	-0.002003643	-0.003113532	0.590013887
Funneliformis_geosporum	0	0	0.076267131	0.607235544
Glomus_intraradices_691_BI	0	0	0.023532582	0.607235544
denovo_10388	0	0	-0.000577746	0.607235544
denovo_10559	0	0	-0.000842356	0.607235544
denovo_11874	0	0	9.46E-05	0.607235544
denovo_182	0	0	0.000513011	0.607235544
denovo_212	0	0	0.000274455	0.607235544
denovo_633	0	0	-0.021653904	0.607235544
denovo_868	0	0	-0.000632552	0.607235544
denovo_9959	0	0	0.046437682	0.607235544
denovo_10993	-2.949644582	-0.003228282	-0.003563561	0.666430034
denovo_414	-0.019006368	-8.07E-06	-0.004087357	0.666430034
denovo_274	-0.829256256	-0.000472847	-0.006555351	0.672335808
denovo_15710	0	0	0.000468556	0.724081661
denovo_32	0	0	-0.000958289	0.724081661
denovo_6735	-Inf	-0.000780762	0.00087912	0.746475972

denovo_10779	0	0	-0.000682577	0.797169693
denovo_263	0	0	0.000289507	0.797169693
denovo_36	0	0	-3.31E-05	0.797169693
denovo_4580	0	0	-0.021925443	0.797169693
denovo_481	0	0	0.000207912	0.797169693
denovo_4870	0	0	-3.05E-05	0.797169693
denovo_5	0	0	-0.00290074	0.797169693
denovo_547	0	0	-0.000161079	0.797169693
denovo_10069	-Inf	-0.000154202	0.000240411	0.823664389
denovo_1185	-Inf	-0.000303306	0.000478812	0.823664389
denovo_580	-1.772012541	-0.000386448	-0.002431669	0.829356961
Rhizophagus_irregularis	-2.891489623	-0.00297239	0.01907586	0.832518813
Sclerocystis_sinuosa_MD12(	0	0	-0.001243511	0.906329391
denovo_7787	0	0	-0.000837019	0.906329391
denovo_10028	0	0	0.002469488	1
denovo_10067	0	0	0.000372272	1
denovo_10098	0	0	-0.000301934	1
denovo_10288	0	0	-0.00565549	1
denovo_11066	0	0	-7.91E-06	1
denovo_11388	0	0	-0.001456724	1
denovo_11903	0	0	9.06E-05	1
denovo_121	0	0	0.00030613	1
denovo_147	0	0	-0.006385363	1
denovo_15	0	0	0.000246418	1
denovo_151	0	0	0.001049146	1
denovo_16034	0	0	0.033595603	1
denovo_16035	0	0	0.006790047	1
denovo_16036	0	0	0.062175583	1
denovo_16039	0	0	0.006496875	1
denovo_16054	0	0	0.007854175	1
denovo_16060	0	0	0.000910418	1
denovo_2	2.286577715	0.007192164	-0.001264432	1
denovo_2077	0	0	2.85E-05	1
denovo_392	0	0	0.000244799	1
denovo_4631	0	0	0.029039297	1
denovo_4642	0	0	-0.000507992	1
denovo_483	1.035117118	0.000163848	0.002767691	1
denovo_4876	0	0	-0.001990271	1
denovo_5282	0	0	-4.53E-05	1
denovo_58	0	0	-0.000216058	1
denovo_6413	0	0	0.001539619	1
denovo_736	0	0	-0.015315546	1
denovo_780	2.620304882	0.000937851	-0.002286462	1
denovo_8299	0	0	-0.000280253	1
denovo_8957	0	0	-1.18E-06	1
denovo_9942	0	0	-0.001314611	1

"Bs1S" vs. "Rs1S"

tax_name	log2_median_ratio	median_diff	mean_diff	wilcox_p_value
Septoglomus_viscosum	-Inf	-0.011714116	-0.044004453	0.02536986
denovo_442	-Inf	-0.00064	-0.001097989	0.04490859

Rhizophagus_diaphanus	Inf	0.000441241	0.001399414	0.072005658
denovo_10505	-Inf	-0.00064	-0.003183375	0.072005658
denovo_10993	-Inf	-0.00048	-0.008473043	0.072005658
denovo_25	-Inf	-8.00E-04	-0.001414996	0.072005658
denovo_4281	-Inf	-0.00014828	-0.013562249	0.072005658
denovo_4634	Inf	0.000152718	0.005917343	0.072005658
denovo_4767	-Inf	-0.000462963	-0.012147217	0.072005658
denovo_6172	-Inf	-0.000153327	-0.000304601	0.072005658
denovo_68	-Inf	-0.00112	-0.001019472	0.072005658
denovo_98	-Inf	-0.00029656	-0.002941406	0.072005658
denovo_39	-Inf	-0.0056	-0.003971897	0.074639183
denovo_3	-Inf	-0.004744958	-0.009132084	0.105997548
denovo_18	-Inf	-0.00208	-0.002080884	0.118797498
denovo_21	-Inf	-0.0014828	-0.002550218	0.118797498
denovo_90	-Inf	-0.001521838	-0.004784827	0.118797498
Rhizophagus_sp	Inf	0.017350634	0.047036156	0.157939311
denovo_274	-Inf	-0.000608735	-0.000923047	0.157939311
denovo_6577	-Inf	-0.0007414	-0.000896398	0.157939311
denovo_780	-Inf	-0.00112	-0.032975334	0.157939311
denovo_17	Inf	0.004677127	0.024865398	0.161237592
Rhizophagus_intraradices	0	0	0.064104912	0.179712495
Sclerocystis_sinuosa_MD12(	0	0	-6.24E-05	0.179712495
Septoglomus_jasnowskae	0	0	-0.006627559	0.179712495
denovo_10048	0	0	-0.0007734	0.179712495
denovo_10067	0	0	-0.001974621	0.179712495
denovo_10069	0	0	-0.00069931	0.179712495
denovo_10098	0	0	-9.29E-05	0.179712495
denovo_10217	0	0	-0.000614216	0.179712495
denovo_10455	0	0	-0.001334629	0.179712495
denovo_10699	0	0	-0.001230621	0.179712495
denovo_10779	0	0	-0.00015531	0.179712495
denovo_11056	0	0	-0.000252874	0.179712495
denovo_1131	0	0	0.002612378	0.179712495
denovo_1185	0	0	-0.001904963	0.179712495
denovo_11874	0	0	-0.000773075	0.179712495
denovo_12430	0	0	-0.000476515	0.179712495
denovo_15710	0	0	-0.000535827	0.179712495
denovo_2075	0	0	0.072011213	0.179712495
denovo_2084	0	0	0.02226075	0.179712495
denovo_2097	0	0	0.010533101	0.179712495
denovo_2113	0	0	0.004215465	0.179712495
denovo_212	0	0	-0.000366976	0.179712495
denovo_2137	0	0	0.003578894	0.179712495
denovo_2163	0	0	0.002445218	0.179712495
denovo_2452	0	0	0.004758906	0.179712495
denovo_2605	0	0	0.000873222	0.179712495
denovo_263	0	0	-0.000998837	0.179712495
denovo_2678	0	0	0.00077899	0.179712495
denovo_2819	0	0	0.005520174	0.179712495
denovo_3585	0	0	0.001160145	0.179712495
denovo_4580	0	0	-0.000216968	0.179712495

denovo_4632	0	0	0.000501591	0.179712495
denovo_5162	0	0	0.00592363	0.179712495
denovo_5180	0	0	0.001343553	0.179712495
denovo_5181	0	0	0.0030481	0.179712495
denovo_5187	0	0	0.000671592	0.179712495
denovo_5216	0	0	0.000762855	0.179712495
denovo_5382	0	0	0.000698081	0.179712495
denovo_547	0	0	-0.000671611	0.179712495
denovo_6072	0	0	0.000541297	0.179712495
denovo_609	0	0	0.017042303	0.179712495
denovo_610	0	0	0.02996602	0.179712495
denovo_611	0	0	0.000634044	0.179712495
denovo_6133	0	0	-0.000509331	0.179712495
denovo_6134	0	0	-0.001590565	0.179712495
denovo_614	0	0	0.004574123	0.179712495
denovo_615	0	0	0.041285614	0.179712495
denovo_624	0	0	0.008057098	0.179712495
denovo_627	0	0	0.016492849	0.179712495
denovo_632	0	0	0.003498514	0.179712495
denovo_637	0	0	-0.016381656	0.179712495
denovo_645	0	0	0.001514649	0.179712495
denovo_700	0	0	0.004883984	0.179712495
denovo_736	0	0	-0.015615537	0.179712495
denovo_818	0	0	0.002350389	0.179712495
denovo_840	0	0	0.004559377	0.179712495
denovo_911	0	0	0.000487224	0.179712495
denovo_913	0	0	0.000966706	0.179712495
denovo_9950	0	0	-0.043052669	0.179712495
denovo_9959	0	0	-0.057500722	0.179712495
denovo_12	-Inf	-0.018109877	-0.013538623	0.181220536
denovo_30	-Inf	-0.00044484	-0.007756933	0.181220536
denovo_231	-Inf	-0.00029656	-0.001073926	0.239316541
denovo_8699	-Inf	-0.00014828	-0.037212871	0.239316541
denovo_1926	Inf	0.00091631	0.008606951	0.265205393
denovo_1	-Inf	-0.02	-0.031391301	0.332111978
denovo_483	-Inf	-0.00032	-0.002803612	0.346521712
denovo_580	-Inf	-0.00016	-0.000938581	0.346521712
Rhizophagus_irregularis	-Inf	-0.000462963	-0.040222152	0.37274807
denovo_5	Inf	0.000305437	0.078285114	0.37274807
Glomus_sp	-2.465424919	-0.068921617	-0.128908664	0.420634921
Glomus_cf	0	0	0.001500221	0.423710797
Glomus_intraradices_x99640	0	0	-4.63E-05	0.423710797
Glomus_invermaium	0	0	0.016065974	0.423710797
denovo_10028	0	0	-0.002624	0.423710797
denovo_10288	0	0	-6.40E-05	0.423710797
denovo_10388	0	0	-0.000224	0.423710797
denovo_10559	0	0	-0.000192	0.423710797
denovo_1071	0	0	0.001357876	0.423710797
denovo_1092	0	0	0.000633675	0.423710797
denovo_11066	0	0	-0.000517425	0.423710797
denovo_11388	0	0	-0.000182621	0.423710797

denovo_11903	0	0	-0.00033732	0.423710797
denovo_121	0	0	-0.000398651	0.423710797
denovo_1446	0	0	0.000366524	0.423710797
denovo_1506	0	0	0.000754375	0.423710797
denovo_16034	0	0	-0.033657407	0.423710797
denovo_16035	0	0	-0.006851852	0.423710797
denovo_16036	0	0	-0.062453704	0.423710797
denovo_16039	0	0	-0.006527778	0.423710797
denovo_16054	0	0	-0.008472222	0.423710797
denovo_16060	0	0	-0.000972222	0.423710797
denovo_164	0	0	9.73E-05	0.423710797
denovo_2115	0	0	0.002105879	0.423710797
denovo_3738	0	0	0.001191203	0.423710797
denovo_3952	0	0	6.04E-05	0.423710797
denovo_4	0	0	0.058208955	0.423710797
denovo_4477	0	0	0.0007242	0.423710797
denovo_4727	0	0	8.82E-05	0.423710797
denovo_4732	0	0	-9.26E-05	0.423710797
denovo_481	0	0	-0.000582643	0.423710797
denovo_4870	0	0	-0.000121747	0.423710797
denovo_4876	0	0	-0.000122662	0.423710797
denovo_5174	0	0	0.000946854	0.423710797
denovo_5185	0	0	3.05E-05	0.423710797
denovo_6124	0	0	-0.008371665	0.423710797
denovo_6125	0	0	-0.002272	0.423710797
denovo_6128	0	0	-0.003434529	0.423710797
denovo_6131	0	0	-9.60E-05	0.423710797
denovo_6135	0	0	-3.20E-05	0.423710797
denovo_6151	0	0	-0.000256	0.423710797
denovo_6155	0	0	-0.000352	0.423710797
denovo_6171	0	0	-3.20E-05	0.423710797
denovo_6175	0	0	-0.000521312	0.423710797
denovo_6176	0	0	0.0001207	0.423710797
denovo_6233	0	0	-0.001931923	0.423710797
denovo_6276	0	0	-0.000275989	0.423710797
denovo_633	0	0	-0.011872	0.423710797
denovo_6384	0	0	-3.20E-05	0.423710797
denovo_6400	0	0	-0.000576	0.423710797
denovo_6413	0	0	-0.001601423	0.423710797
denovo_642	0	0	0.001599276	0.423710797
denovo_6566	0	0	-0.001165287	0.423710797
denovo_657	0	0	0.001679902	0.423710797
denovo_6598	0	0	6.04E-05	0.423710797
denovo_667	0	0	0.006819553	0.423710797
denovo_6808	0	0	-0.00033732	0.423710797
denovo_691	0	0	0.001870851	0.423710797
denovo_735	0	0	0.0001207	0.423710797
denovo_754	0	0	0.0001207	0.423710797
denovo_7540	0	0	-0.000459982	0.423710797
denovo_7630	0	0	-0.003403864	0.423710797
denovo_797	0	0	0.000995775	0.423710797

denovo_8180	0	0	0.000452625	0.423710797
denovo_8186	0	0	0.000331925	0.423710797
denovo_851	0	0	0.0001207	0.423710797
denovo_859	0	0	0.00091631	0.423710797
denovo_9391	0	0	-4.63E-05	0.423710797
denovo_9942	0	0	-0.00672	0.423710797
Glomus_aggregatum	0	0	-0.015452784	0.440686016
denovo_2077	0	0	0.018485454	0.440686016
denovo_6209	0	0	-0.000341814	0.440686016
denovo_6735	0	0	-0.002269711	0.440686016
denovo_7787	0	0	-0.00041085	0.440686016
denovo_8438	0	0	0.000660442	0.440686016
denovo_2	-Inf	-0.009046305	0.034368122	0.450643728
Rhizophagus_cf	1.338576446	0.002123634	0.171847155	0.600401848
Glomus_intraradices_691_BI	0	0	-0.024171236	0.607235544
denovo_182	0	0	-0.000686811	0.607235544
denovo_770	0	0	0.003282145	0.607235544
denovo_8	-Inf	-0.00029656	0.006788163	0.655836497
denovo_147	0	0	7.23E-05	0.797169693
denovo_32	0	0	7.57E-06	0.797169693
denovo_36	0	0	0.001574145	0.797169693
denovo_8159	0	0	0.028465669	0.797169693
Funneliformis_geosporum	0	0	-0.074447444	0.906329391
denovo_15	0	0	0.001817783	1
denovo_151	0	0	-0.005013103	1
denovo_16053	0	0	-0.000657843	1
denovo_167	0	0	-0.000318661	1
denovo_392	0	0	-0.00023998	1
denovo_397	0	0	6.67E-05	1
denovo_414	-Inf	-0.000608735	0.002071931	1
denovo_4631	0	0	-0.029164083	1
denovo_4642	0	0	0.0011604	1
denovo_5165	0	0	0.004335746	1
denovo_5282	0	0	0.000394155	1
denovo_58	0	0	0.000553371	1
denovo_811	Inf	0.000441241	-0.001371603	1
denovo_8218	0	0	-9.81E-07	1
denovo_8299	0	0	-6.48E-05	1
denovo_8447	0	0	0.000484357	1
denovo_868	0	0	-3.98E-05	1
denovo_8957	0	0	6.09E-05	1

"Bs2S" vs. "Rs2S"

tax_name	log2_median_ratio	median_diff	mean_diff	wilcox_p_value
Septoglomus_viscosum	-9.4337594	-0.103691199	-0.178131047	0.007936508
denovo_10505	-Inf	-0.009993754	-0.00826537	0.02536986
denovo_610	Inf	0.001068539	0.065222688	0.02536986
Glomus_aggregatum	-Inf	-0.009525297	-0.019196254	0.066955282
denovo_10069	-Inf	-0.000154202	-0.000458899	0.072005658
denovo_10993	-Inf	-0.003708282	-0.012036605	0.072005658
denovo_11021	-Inf	-0.000182149	-0.002144679	0.072005658

denovo_11042	-Inf	-0.000182149	-0.000683664	0.072005658
denovo_11056	-Inf	-0.001233616	-0.001846412	0.072005658
denovo_4732	Inf	0.000299356	0.034160536	0.072005658
denovo_615	Inf	0.001938273	0.009729126	0.072005658
denovo_619	Inf	0.000146671	0.000418592	0.072005658
denovo_700	Inf	0.000586682	0.000356735	0.072005658
denovo_8438	-Inf	-0.000312305	-0.000772596	0.072005658
denovo_8447	-Inf	-0.000308404	-0.001595	0.072005658
denovo_3745	Inf	0.002595024	0.002372899	0.074639183
denovo_5	Inf	0.038438438	0.035936319	0.074639183
Glomus_sp	-4.881339302	-0.196044641	-0.132517659	0.095238095
denovo_17	3.181905934	0.07715018	0.045637762	0.095238095
Rhizophagus_irregularis	-3.52052237	-0.003135997	-0.023821641	0.116073943
denovo_3882	Inf	0.0003003	0.000710525	0.157939311
denovo_4242	Inf	0.00164646	0.001071503	0.157939311
Glomus_intraradices_x99640	0	0	-0.017081107	0.179712495
Rhizophagus_diaphanus	0	0	0.001213379	0.179712495
denovo_10098	0	0	-0.000394808	0.179712495
denovo_10388	0	0	-0.000801746	0.179712495
denovo_10559	0	0	-0.001034356	0.179712495
denovo_11040	0	0	-0.001278481	0.179712495
denovo_1131	0	0	0.002174888	0.179712495
denovo_1134	0	0	0.000714699	0.179712495
denovo_14551	0	0	-0.011876881	0.179712495
denovo_15710	0	0	-6.73E-05	0.179712495
denovo_2163	0	0	-0.000186603	0.179712495
denovo_316	0	0	-0.00052968	0.179712495
denovo_32	0	0	-0.001112951	0.179712495
denovo_4281	0	0	0.000238978	0.179712495
denovo_4767	0	0	0.0006021	0.179712495
denovo_609	0	0	0.131152748	0.179712495
denovo_611	0	0	0.044480487	0.179712495
denovo_624	0	0	0.001262759	0.179712495
denovo_627	0	0	0.000881415	0.179712495
denovo_632	0	0	0.010761885	0.179712495
denovo_637	0	0	0.006308639	0.179712495
denovo_667	0	0	0.001216778	0.179712495
denovo_691	0	0	0.005443877	0.179712495
denovo_8186	0	0	-0.001096161	0.179712495
denovo_8218	0	0	-0.000950441	0.179712495
denovo_840	0	0	0.000803673	0.179712495
denovo_859	0	0	0.001609306	0.179712495
denovo_970	0	0	0.002651516	0.179712495
denovo_483	-Inf	-0.000156152	-0.000302743	0.239316541
Rhizophagus_intraradices	Inf	0.001951952	0.101891663	0.265205393
denovo_8159	-Inf	-0.002003643	-0.000943453	0.265205393
denovo_2	-Inf	-0.001854141	0.036716576	0.332111978
denovo_274	-Inf	-0.001081582	-0.005869028	0.332111978
denovo_6735	-Inf	-0.000780762	-0.000888085	0.332111978
denovo_837	Inf	0.00015015	0.000473953	0.332111978
Glomus_cf	Inf	0.000149098	0.005662502	0.346521712

denovo_414	-Inf	-0.000616808	-0.003605291	0.346521712
denovo_580	-Inf	-0.000546448	-0.002482679	0.346521712
denovo_98	Inf	0.0003003	0.005103059	0.346521712
Rhizophagus_sp	Inf	0.000610594	0.005676242	0.37274807
denovo_10028	0	0	-0.000154512	0.423710797
denovo_10288	0	0	-0.00571949	0.423710797
denovo_10455	0	0	-6.18E-05	0.423710797
denovo_10699	0	0	-3.09E-05	0.423710797
denovo_1071	0	0	0.00438348	0.423710797
denovo_10779	0	0	-0.000837887	0.423710797
denovo_1092	0	0	2.93E-05	0.423710797
denovo_11066	0	0	-0.00052534	0.423710797
denovo_11388	0	0	-0.001639344	0.423710797
denovo_11874	0	0	-0.000678489	0.423710797
denovo_11903	0	0	-0.000246723	0.423710797
denovo_121	0	0	-9.25E-05	0.423710797
denovo_12430	0	0	-3.08E-05	0.423710797
denovo_1446	0	0	2.93E-05	0.423710797
denovo_15	0	0	-0.000401731	0.423710797
denovo_1506	0	0	0.000477113	0.423710797
denovo_16034	0	0	-6.18E-05	0.423710797
denovo_16035	0	0	-6.18E-05	0.423710797
denovo_16036	0	0	-0.000278121	0.423710797
denovo_16039	0	0	-3.09E-05	0.423710797
denovo_16053	0	0	2.93E-05	0.423710797
denovo_16054	0	0	-0.000618047	0.423710797
denovo_16060	0	0	-6.18E-05	0.423710797
denovo_164	0	0	0.001021021	0.423710797
denovo_167	0	0	0.000750751	0.423710797
denovo_2077	0	0	-3.08E-05	0.423710797
denovo_2097	0	0	6.01E-05	0.423710797
denovo_2115	0	0	-3.08E-05	0.423710797
denovo_2452	0	0	-6.18E-05	0.423710797
denovo_2605	0	0	0.00012012	0.423710797
denovo_263	0	0	-0.000709329	0.423710797
denovo_2678	0	0	-3.08E-05	0.423710797
denovo_3952	0	0	0.000477113	0.423710797
denovo_397	0	0	0.000329292	0.423710797
denovo_4	0	0	-0.017409766	0.423710797
denovo_4477	0	0	0.000298196	0.423710797
denovo_4631	0	0	-0.000154202	0.423710797
denovo_4727	0	0	0.000702183	0.423710797
denovo_5165	0	0	6.01E-05	0.423710797
denovo_5174	0	0	-0.000123362	0.423710797
denovo_5282	0	0	-0.00010929	0.423710797
denovo_547	0	0	-0.000832691	0.423710797
denovo_6124	0	0	0.036426426	0.423710797
denovo_6125	0	0	0.004744745	0.423710797
denovo_6128	0	0	0.000780781	0.423710797
denovo_6131	0	0	0.00039039	0.423710797
denovo_6133	0	0	0.000840841	0.423710797

denovo_6135	0	0	0.000780781	0.423710797
denovo_6151	0	0	0.001291291	0.423710797
denovo_6155	0	0	0.00042042	0.423710797
denovo_6171	0	0	0.00042042	0.423710797
denovo_6175	0	0	0.000630631	0.423710797
denovo_6209	0	0	0.000600601	0.423710797
denovo_6233	0	0	0.001141141	0.423710797
denovo_6276	0	0	0.001111111	0.423710797
denovo_6384	0	0	0.00039039	0.423710797
denovo_6400	0	0	3.00E-05	0.423710797
denovo_642	0	0	0.003930771	0.423710797
denovo_645	0	0	0.002346729	0.423710797
denovo_6566	0	0	0.00042042	0.423710797
denovo_657	0	0	0.000322675	0.423710797
denovo_6808	0	0	6.01E-05	0.423710797
denovo_754	0	0	0.00074549	0.423710797
denovo_7540	0	0	3.00E-05	0.423710797
denovo_7630	0	0	6.01E-05	0.423710797
denovo_770	0	0	8.80E-05	0.423710797
denovo_797	0	0	2.93E-05	0.423710797
denovo_8180	0	0	-0.000648949	0.423710797
denovo_8299	0	0	-0.000556242	0.423710797
denovo_851	0	0	0.000536753	0.423710797
denovo_8699	0	0	-0.071657559	0.423710797
denovo_913	0	0	0.000146671	0.423710797
denovo_9391	0	0	0.002394851	0.423710797
denovo_9942	0	0	-0.008034611	0.423710797
denovo_9950	0	0	-3.03E-05	0.423710797
denovo_9959	0	0	-0.011063041	0.423710797
denovo_4788	0	0	0.00082153	0.440686016
denovo_481	0	0	-0.000344702	0.440686016
denovo_614	0	0	0.015577593	0.440686016
denovo_6598	0	0	0.000389391	0.440686016
denovo_736	0	0	-0.030872415	0.440686016
denovo_7787	0	0	-0.001315179	0.440686016
Glomus_intraradices_691_BI	Inf	0.000152648	-0.000610002	0.48017689
denovo_12	Inf	0.003813435	-0.00086915	0.48017689
denovo_3	-Inf	-0.004471858	0.013018318	0.48017689
denovo_8	Inf	0.0003003	-0.020386866	0.48017689
denovo_868	Inf	0.000440012	0.00433538	0.503812429
Funneliformis_geosporum	0	0	0.005998352	0.607235544
Sclerocystis_sinuosa_MD12(	0	0	-0.001216141	0.607235544
denovo_18	0	0	-0.000683108	0.607235544
denovo_30	0	0	0.003331329	0.607235544
denovo_4642	0	0	-0.000545502	0.607235544
denovo_4193	Inf	0.000146671	0.000223482	0.655836497
denovo_633	Inf	0.000600601	-0.009691944	0.655836497
denovo_811	0.615805364	0.003068199	0.003330824	0.69047619
denovo_10067	0	0	-0.001480729	0.724081661
denovo_3738	0	0	0.003914437	0.724081661
denovo_4750	0	0	0.002567729	0.724081661

denovo_4870	0	0	0.001414527	0.724081661
denovo_6146	0	0	0.000617828	0.724081661
denovo_6185	0	0	0.000851102	0.724081661
denovo_772	0	0	0.001897231	0.724081661
Glomus_invermaium	0	0	-0.044765283	0.797169693
denovo_39	0	0	0.002618708	0.797169693
denovo_4580	0	0	-0.022052742	0.797169693
denovo_5185	0	0	0.000116406	0.797169693
denovo_6577	0	0	0.000314809	0.797169693
denovo_1	-Inf	-0.000468457	-0.022315763	0.823664389
denovo_780	2.043212067	0.000568601	-0.014067235	0.832518813
denovo_1185	0.536767788	0.000136706	-0.000308928	0.83403523
Rhizophagus_cf	-1.106975875	-0.002924843	-0.0109843	1
denovo_147	0	0	-0.006570815	1
denovo_151	0	0	-0.004225945	1
denovo_182	0	0	0.000800276	1
denovo_1926	0.95868943	0.000145789	0.04344447	1
denovo_21	0	0	0.001103818	1
denovo_212	0	0	2.76E-05	1
denovo_231	0	0	0.000969822	1
denovo_25	0	0	-0.000135517	1
denovo_2819	0	0	8.93E-05	1
denovo_36	0	0	-0.000218597	1
denovo_392	0	0	2.72E-05	1
denovo_442	0	0	-0.001483581	1
denovo_4634	0	0	-0.002352365	1
denovo_4734	0	0	0.000794568	1
denovo_4876	0	0	-0.002021344	1
denovo_58	0	0	-9.66E-05	1
denovo_6134	0	0	0.006063635	1
denovo_6172	0	0	0.001041327	1
denovo_6176	0	0	0.003818244	1
denovo_6413	0	0	2.83E-05	1
denovo_68	0	0	-0.000494257	1
denovo_735	0	0	-0.000987622	1
denovo_818	-Inf	-0.000154512	0.003894824	1
denovo_8957	0	0	0.00044813	1
denovo_90	Inf	0.000146671	-0.000343565	1

Table S4. LEfSe analysis results using non-parametric factorial Kruskal-Wallis (KW) sum-rank test. Adjusted p-value cut-off=0.05 and LDA score =1.0. Significant taxa are highlighted in yellow.

	P values	FDR	Bs1S	Rs1S	Bs2S	Rs2S	LDA score
Septoglomus_viscosum	0.0037752	0.45506	0	441290	2725.4	1788200	5.95
denovo_3745	0.010582	0.45506	0	0	27021	3293.7	4.13
denovo_10505	0.013811	0.45506	0	31850	0	82887	4.62
denovo_17	0.01469	0.45506	257470	7758.9	655850	199840	5.51
denovo_619	0.018732	0.45506	0	0	4187.6	0	3.32
denovo_11042	0.018732	0.45506	0	0	0	6860.7	3.54
denovo_11021	0.018732	0.45506	0	0	0	21482	4.03
denovo_811	0.021238	0.45506	3012.2	16737	122740	89616	4.78
denovo_837	0.022761	0.45506	0	0	9090.2	4367.7	3.66
denovo_610	0.030251	0.45506	300030	0	652230	0	5.51
denovo_10993	0.047572	0.45506	0	84894	0	120400	4.78
denovo_3882	0.051023	0.45506	0	0	7473	365.97	3.57
denovo_4242	0.051023	0.45506	0	0	11444	731.93	3.76
denovo_11056	0.059326	0.45506	0	2530.6	0	18465	3.97
denovo_4767	0.07584	0.45506	0	122020	6050.3	0	4.79
denovo_4732	0.078992	0.45506	0	1352.7	341610	0	5.23
denovo_1185	0.082702	0.45506	0	26947	11174	14322	4.13
denovo_4193	0.083476	0.45506	0	0	5118.1	2872	3.41
denovo_4281	0.083476	0.45506	0	136180	2393.2	0	4.83
denovo_10069	0.090269	0.45506	0	6998.9	0	4596.6	3.54
Rhizophagus_diaphanus	0.090269	0.45506	14000	0	12134	0	3.85
denovo_442	0.091285	0.45506	985.55	12038	1208.1	16042	3.88
denovo_911	0.097217	0.45506	4875.9	0	0	0	3.39
denovo_4632	0.097217	0.45506	5017.7	0	0	0	3.4
denovo_316	0.097217	0.45506	0	0	0	5317.1	3.42
denovo_6072	0.097217	0.45506	5428.1	0	0	0	3.43
denovo_10217	0.097217	0.45506	0	6151.7	0	0	3.49

	P values	FDR	Bs1S	Rs1S	Bs2S	Rs2S	LDA score
denovo_5187	0.097217	0.45506	6716.8	0	0	0	3.53
denovo_5382	0.097217	0.45506	6991.8	0	0	0	3.54
denovo_1134	0.097217	0.45506	0	0	7147	0	3.55
denovo_5216	0.097217	0.45506	7630.4	0	0	0	3.58
denovo_10048	0.097217	0.45506	0	7749.7	0	0	3.59
denovo_3585	0.097217	0.45506	11625	0	0	0	3.76
denovo_11040	0.097217	0.45506	0	0	0	12792	3.81
denovo_5180	0.097217	0.45506	13436	0	0	0	3.83
denovo_970	0.097217	0.45506	0	0	26515	0	4.12
denovo_5181	0.097217	0.45506	30497	0	0	0	4.18
denovo_2137	0.097217	0.45506	35848	0	0	0	4.25
denovo_2113	0.097217	0.45506	42194	0	0	0	4.32
denovo_5162	0.097217	0.45506	59241	0	0	0	4.47
Septoglomus_jasnowskae	0.097217	0.45506	0	66384	0	0	4.52
denovo_14551	0.097217	0.45506	0	0	0	119300	4.78
denovo_2084	0.097217	0.45506	222800	0	0	0	5.05
denovo_2075	0.097217	0.45506	720750	0	0	0	5.56
denovo_700	0.10016	0.45506	48923	0	3574.2	0	4.39
denovo_615	0.10016	0.45506	413180	0	97324	0	5.32
Glomus_aggregatum	0.11006	0.48942	294.16	154830	909.34	193010	4.98
denovo_21	0.1464	0.57795	3942.2	29574	13893	2776.5	4.13
denovo_68	0.16167	0.57795	0	10252	302.02	5244.5	3.71
denovo_8438	0.1632	0.57795	6968.8	309.74	0	7743.1	3.59
Glomus_sp	0.16522	0.57795	352290	1643200	646950	1973000	5.91
denovo_98	0.16867	0.57795	0	29699	76323	25297	4.58
denovo_15710	0.1894	0.57795	0	5374.3	0	674.46	3.43
denovo_6146	0.1894	0.57795	0	0	9638.1	3436.9	3.68
denovo_6185	0.1894	0.57795	0	0	12340	3802.8	3.79
denovo_772	0.1894	0.57795	0	0	21412	2450.4	4.03

	**P values**	**FDR**	**Bs1S**	**Rs1S**	**Bs2S**	**Rs2S**	**LDA score**
denovo_2163	0.1894	0.57795	24486	0	0	1866.2	4.09
denovo_4750	0.1894	0.57795	0	0	28413	2736.7	4.15
denovo_632	0.1894	0.57795	35020	0	107620	0	4.73
denovo_611	0.1894	0.57795	6358.7	0	444800	0	5.35
denovo_609	0.1894	0.57795	170880	0	1311500	0	5.82
denovo_8447	0.19447	0.57795	5750.6	891.53	0	16019	3.9
denovo_840	0.1951	0.57795	45724	0	8036.7	0	4.36
denovo_627	0.1951	0.57795	164930	0	8817.6	0	4.92
denovo_39	0.19641	0.57795	985.55	40864	35337	8958.9	4.3
denovo_10098	0.19704	0.57795	0	929.04	0	3948.2	3.3
denovo_1131	0.19704	0.57795	26159	0	21749	0	4.12
denovo_624	0.19704	0.57795	80814	0	12631	0	4.61
denovo_637	0.19704	0.57795	0	163970	63086	0	4.91
denovo_5	0.20558	0.57795	800750	15411	403990	44369	5.59
Rhizophagus_intraradices	0.21157	0.57795	641060	0	1020000	1035.2	5.71
denovo_4788	0.22218	0.57795	0	0	8518.6	303.31	3.63
denovo_10699	0.22218	0.57795	0	12421	0	310.27	3.79
denovo_10455	0.22218	0.57795	0	13446	0	620.54	3.83
denovo_2452	0.22218	0.57795	47707	0	0	620.54	4.38
denovo_2097	0.22218	0.57795	105430	0	604.05	0	4.72
Glomus_intraradices_x996	0.22218	0.57795	0	676.36	0	171220	4.93
denovo_9950	0.22218	0.57795	0	431320	0	303.31	5.33
denovo_8699	0.22581	0.57795	2724	374900	0	719850	5.56
denovo_25	0.22957	0.57795	0	14247	4530.4	5861.5	3.85
Glomus_cf	0.22957	0.57795	15002	0	63183	6558.4	4.5
denovo_274	0.23251	0.57795	985.55	10274	17074	76086	4.57
denovo_633	0.24333	0.57795	0	118830	238350	335260	5.22
denovo_90	0.25086	0.57795	7227.3	55273	23231	26554	4.38
denovo_11874	0.25441	0.57795	0	7751.7	0	6787	3.59

	P values	FDR	Bs1S	Rs1S	Bs2S	Rs2S	LDA score
denovo_10388	0.25441	0.57795	0	2242.2	0	8046.1	3.6
denovo_2605	0.25441	0.57795	8757.4	0	1208.1	0	3.64
denovo_913	0.25441	0.57795	9693.6	0	1466.7	0	3.69
denovo_8186	0.25441	0.57795	3329.3	0	0	11011	3.74
denovo_859	0.25441	0.57795	9163.1	0	16093	0	3.91
denovo_691	0.25441	0.57795	18765	0	54439	0	4.43
denovo_9959	0.25441	0.57795	0	579990	0	111080	5.46
denovo_12	0.26385	0.58169	67346	203070	60532	69104	4.85
denovo_4634	0.27133	0.58169	59284	0	1769.8	25295	4.47
denovo_6735	0.27293	0.58169	985.55	23779	6019.1	14907	4.06
denovo_30	0.28178	0.58169	10841	89125	61071	27765	4.59
denovo_10779	0.28389	0.58169	0	1553.7	0	8417.2	3.62
denovo_547	0.28389	0.58169	0	6776.6	0	8329.5	3.62
denovo_6133	0.28389	0.58169	0	5103.8	8456.7	0	3.63
denovo_263	0.28389	0.58169	0	10058	0	7095.5	3.7
denovo_645	0.28389	0.58169	15178	0	23467	0	4.07
denovo_667	0.28389	0.58169	68402	0	12168	0	4.53
denovo_3	0.29079	0.59005	4219	95731	191480	60402	4.97
Rhizophagus_sp	0.3019	0.60271	471390	1352.7	70135	13372	5.37
denovo_6172	0.3028	0.60271	0	3053.7	14195	3702	3.85
Rhizophagus_irregularis	0.32521	0.63361	28862	431810	2111.5	240560	5.33
denovo_818	0.33308	0.63361	23508	0	47765	8815.5	4.38
denovo_780	0.34105	0.63361	1642.6	332910	213580	354260	5.25
denovo_18	0.34443	0.63361	7227.3	28083	13289	20068	4.02
denovo_614	0.36353	0.63361	45835	0	157320	1551.3	4.9
denovo_735	0.3664	0.63361	1210.7	0	9621.3	19539	3.99
denovo_736	0.3664	0.63361	0	156250	586.68	310530	5.19
denovo_32	0.40859	0.63361	1642.6	1559.3	0	11142	3.75
denovo_4870	0.40859	0.63361	0	1217.5	15668	1523.5	3.89

	**P values**	**FDR**	**Bs1S**	**Rs1S**	**Bs2S**	**Rs2S**	**LDA score**
denovo_8218	0.41015	0.63361	605.33	619.48	0	9546.9	3.68
denovo_2077	0.41015	0.63361	185630	594.35	0	308.5	4.97
denovo_868	0.41438	0.63361	882.48	1281.2	50959	7610.5	4.4
denovo_10067	0.42117	0.63361	0	19763	1217.9	16078	3.99
denovo_483	0.4401	0.63361	3613.7	31908	906.07	3935.7	4.19
denovo_3738	0.44792	0.63361	11912	0	41193	2034.8	4.31
denovo_6598	0.45517	0.63361	605.33	0	4223	308.5	3.32
denovo_2819	0.45517	0.63361	55368	0	1208.1	308.5	4.44
Sclerocystis_sinuosa_MD	0.46169	0.63361	0	624.68	898.07	13119	3.82
denovo_231	0.47346	0.63361	2956.6	13751	12685	2927.7	3.73
denovo_580	0.47848	0.63361	2299.6	11759	11147	35989	4.23
denovo_4580	0.48299	0.63361	0	2172.8	898.42	221420	5.04
denovo_5185	0.4953	0.63361	305.44	0	3624.3	2450.4	3.26
denovo_481	0.4953	0.63361	0	5885.1	302.02	3757.3	3.47
denovo_6209	0.4953	0.63361	302.66	3748.6	6040.5	0	3.48
denovo_6134	0.4953	0.63361	0	16056	61915	925.5	4.49
denovo_212	0.50409	0.63361	0	3704.3	1208.1	925.5	3.27
denovo_770	0.50409	0.63361	34204	1281.2	880.02	0	4.23
denovo_6577	0.51153	0.63361	907.99	9933.4	9060.7	5865	3.65
denovo_8159	0.54309	0.63361	318100	32661	54184	63848	5.15
denovo_11903	0.54873	0.63361	0	3407.2	0	2468	3.23
denovo_6808	0.54873	0.63361	0	3407.2	604.05	0	3.23
denovo_1446	0.54873	0.63361	3665.2	0	293.34	0	3.26
denovo_6131	0.54873	0.63361	0	960.92	3926.3	0	3.29
denovo_6384	0.54873	0.63361	0	320.31	3926.3	0	3.29
denovo_121	0.54873	0.63361	0	4026.6	0	925.5	3.3
denovo_6155	0.54873	0.63361	0	3523.4	4228.3	0	3.33
denovo_6171	0.54873	0.63361	0	320.31	4228.3	0	3.33
denovo_7540	0.54873	0.63361	0	4646.1	302.02	0	3.37

	P values	FDR	Bs1S	Rs1S	Bs2S	Rs2S	LDA score
denovo_3952	0.54873	0.63361	605.33	0	4771.1	0	3.38
denovo_11066	0.54873	0.63361	0	5174.3	0	5274.6	3.42
denovo_851	0.54873	0.63361	1210.7	0	5367.5	0	3.43
denovo_6400	0.54873	0.63361	0	5765.5	302.02	0	3.46
denovo_1092	0.54873	0.63361	6355.9	0	293.34	0	3.5
denovo_6175	0.54873	0.63361	0	5265.6	6342.5	0	3.5
denovo_8180	0.54873	0.63361	4540	0	0	6515.7	3.51
denovo_4727	0.54873	0.63361	882.48	0	7021.8	0	3.55
denovo_4477	0.54873	0.63361	7263.9	0	2982	0	3.56
denovo_754	0.54873	0.63361	1210.7	0	7454.9	0	3.57
denovo_1506	0.54873	0.63361	7566.6	0	4771.1	0	3.58
denovo_6135	0.54873	0.63361	0	320.31	7852.6	0	3.59
denovo_4734	0.54873	0.63361	0	0	9158.9	1213.2	3.66
denovo_5174	0.54873	0.63361	9468.5	0	0	1234	3.68
denovo_797	0.54873	0.63361	9987.9	0	293.34	0	3.7
denovo_164	0.54873	0.63361	985.55	0	10269	0	3.71
denovo_6276	0.54873	0.63361	0	2787.7	11175	0	3.75
denovo_6566	0.54873	0.63361	0	11770	4228.3	0	3.77
denovo_6151	0.54873	0.63361	0	2562.5	12987	0	3.81
denovo_16060	0.54873	0.63361	0	14204	0	620.54	3.85
denovo_11388	0.54873	0.63361	0	1826.2	0	16468	3.92
denovo_657	0.54873	0.63361	16799	0	3226.8	0	3.92
denovo_2115	0.54873	0.63361	21077	0	0	308.5	4.02
denovo_9391	0.54873	0.63361	0	676.36	23949	0	4.08
denovo_10028	0.54873	0.63361	0	26265	0	1551.3	4.12
denovo_6128	0.54873	0.63361	0	34691	7852.6	0	4.24
denovo_7630	0.54873	0.63361	0	34381	604.05	0	4.24
denovo_642	0.54873	0.63361	16041	0	39308	0	4.29
denovo_1071	0.54873	0.63361	13620	0	43835	0	4.34

	P values	FDR	Bs1S	Rs1S	Bs2S	Rs2S	LDA score
denovo_6125	0.54873	0.63361	0	22742	47720	0	4.38
denovo_10288	0.54873	0.63361	0	640.61	0	57457	4.46
denovo_9942	0.54873	0.63361	0	67265	0	80670	4.61
denovo_16039	0.54873	0.63361	0	95367	0	310.27	4.68
denovo_16035	0.54873	0.63361	0	100100	0	620.54	4.7
denovo_16054	0.54873	0.63361	0	123770	0	6205.4	4.79
denovo_6124	0.54873	0.63361	0	84559	366350	0	5.26
denovo_16034	0.54873	0.63361	0	491710	0	620.54	5.39
denovo_4	0.54873	0.63361	589360	0	0	174100	5.47
denovo_1	0.56646	0.65049	282290	595580	106760	329570	5.39
Glomus_invermaium	0.58353	0.66643	160660	0	35928	483730	5.38
denovo_2	0.6164	0.70015	510050	160520	543030	173410	5.28
denovo_7787	0.63159	0.71352	985.55	5102.3	302.02	13504	3.82
denovo_1926	0.65617	0.73731	87893	1578.4	449040	14593	5.35
denovo_414	0.71112	0.79478	27454	6609.9	11477	47672	4.31
Glomus_intraradices_691_	0.74458	0.80637	3329.3	245030	3619.6	9705.8	5.08
Rhizophagus_cf	0.76034	0.80637	1747700	29132	179050	289110	5.93
denovo_397	0.76778	0.80637	985.55	309.74	3292.9	0	3.22
denovo_5282	0.76778	0.80637	4581.6	640.61	0	1097.9	3.36
denovo_8299	0.76778	0.80637	2118.6	2787.7	0	5584.9	3.45
denovo_167	0.76778	0.80637	985.55	4164	7550.6	0	3.58
denovo_16053	0.76778	0.80637	1756.4	12175	293.34	0	3.78
denovo_6413	0.76778	0.80637	0	16048	906.07	620.54	3.9
denovo_4876	0.76778	0.80637	0	1239	915.89	21226	4.03
denovo_6176	0.76778	0.80637	1210.7	0	39867	1463.9	4.3
denovo_5165	0.76778	0.80637	43677	320.31	604.05	0	4.34
denovo_4631	0.76778	0.80637	294.16	294870	0	1542.5	5.17
denovo_8	0.78312	0.81836	109980	41144	28055	231990	5.01
denovo_36	0.88686	0.91409	18725	2771.6	898.07	3085	3.95

	**P values**	**FDR**	**Bs1S**	**Rs1S**	**Bs2S**	**Rs2S**	**LDA score**
denovo_147	0.88686	0.91409	3207.6	2477.9	598.71	66327	4.52
Funneliformis_geosporum	0.88785	0.91409	22854	1120700	64619	4654	5.75
denovo_182	0.89596	0.91792	2299.6	9150.1	12081	4010.5	3.69
denovo_4642	0.93006	0.94821	14422	2819	2442.4	7899.9	3.78
denovo_392	0.99631	0.99631	985.55	3407.2	1197.4	925.5	3.09
denovo_8957	0.99631	0.99631	907.99	297.18	4789.7	308.5	3.35
denovo_58	0.99631	0.99631	5913.3	309.74	1510.1	2468	3.45
denovo_151	0.99631	0.99631	2956.6	53585	302.02	42573	4.43
